# Final Results of the Telaprevir Access Program: FibroScan Values Predict Safety and Efficacy in Hepatitis C Patients with Advanced Fibrosis or Cirrhosis

**DOI:** 10.1371/journal.pone.0138503

**Published:** 2015-09-23

**Authors:** Antonia Lepida, Massimo Colombo, Inmaculada Fernandez, Djamal Abdurakhmanov, Paulo Abrao Ferreira, Simone I. Strasser, Petr Urbanek, Alessandra Mangia, José L. Calleja, Wafae Iraqi, Ralph DeMasi, Isabelle Lonjon-Domanec, Christophe Moreno, Heiner Wedemeyer

**Affiliations:** 1 Liver Unit, Department of Gastroenterology, Hepatopancreatology and Digestive Oncology, Erasme University Hospital, Université Libre de Bruxelles, Brussels, Belgium; 2 Department of Medicine, Division of Gastroenterology, Fondazione IRCCS Ca’ Granda Ospedale Maggiore Policlinico, Universita’ degli Studi di Milano, Milan, Italy; 3 Hospital Universitario 12 de Octubre, Sección de Aparato Digestivo, Madrid, Spain; 4 I.M. Sechenov First Moscow State Medical University, E.M. Tareev Clinic for Nephrology, Internal and Occupational Medicine, Moscow, Russia; 5 Outpatient Clinic to HIV and Viral Hepatitis Division of Infectious Disease, Federal University of São Paulo, São Paulo, Brazil; 6 AW Morrow Gastroenterology and Liver Center, Royal Prince Alfred hospital and University of Sydney, Sydney, Australia; 7 Department of Internal Medicine, First Medical Faculty, Charles University, and Central Military Hospital Prague, Prague, Czech Republic; 8 Liver Unit, IRCCS Hospital 'Casa Sollievo della Sofferenza', San Giovanni Rotondo, Italy; 9 Gastroenterology, Hospital Universitario Puerta de Hierro, Universidad Autonoma de Madrid, CIBERehd, Madrid, Spain; 10 Janssen Pharmaceuticals, Paris, France; 11 Janssen Research and development, Titusville, New Jersey, United States of America; 12 Medizinische Hochschule Hannover, Hannover, Germany; The Chinese University of Hong Kong, HONG KONG

## Abstract

**Background:**

Liver stiffness determined by transient elastography is correlated with hepatic fibrosis stage and has high accuracy for detecting severe fibrosis and cirrhosis in chronic hepatitis C patients. We evaluated the clinical value of baseline FibroScan values for the prediction of safety and efficacy of telaprevir-based therapy in patients with advanced fibrosis and cirrhosis in the telaprevir Early Access Program HEP3002.

**Methods:**

1,772 patients with HCV-1 and bridging fibrosis or cirrhosis were treated with telaprevir plus pegylated interferon-α and ribavirin (PR) for 12 weeks followed by PR alone, the total treatment duration depending on virological response and previous response type. Liver fibrosis stage was determined either by liver biopsy or by non-invasive markers. 1,282 patients (72%) had disease stage assessed by FibroScan; among those 46% were classified as Metavir F3 at baseline and 54% as F4.

**Results:**

Overall, 1,139 patients (64%) achieved a sustained virological response (SVR) by intention-to-treat analysis. Baseline FibroScan values were tested for association with SVR and the occurrence of adverse events. By univariate analysis, higher baseline FibroScan values were predictive of lower sustained virological response rates and treatment-related anemia. By multivariate analysis, FibroScan was no longer statistically significant as an independent predictor, but higher FibroScan values were correlated with the occurrence of infections and serious adverse events.

**Conclusions:**

FibroScan has a limited utility as a predictor of safety and efficacy in patients treated with telaprevir-based triple therapy. Nevertheless it can be used in association with other clinical and biological parameters to help determine patients who will benefit from the triple regiments.

**Trial Registration:**

ClinicalTrials.gov NCT01508286

## Introduction

Chronic hepatitis C virus (HCV) infection is a major public health issue as it affects approximately 180 million people worldwide [1] and is a leading cause of cirrhosis, end stage liver disease and hepatocellular carcinoma (HCC) and one of the most common indications of liver transplantation in the Western countries [1, 2]. Virus eradication arrests the progression of liver disease and diminishes the rates of liver specific and all causes of mortality [3–5].

Chronic HCV infection management has evolved over recent years from pegylated interferon-α and ribavirin (PR) to combination of direct-acting antiviral agents (DAA) with PR after approval of HCV NS3/4A serine protease inhibitors (PI), telaprevir and boceprevir [1]. Limited data are available on the safety and efficacy of these PIs in patients with advanced liver fibrosis or cirrhosis, as these patients were under-represented in Phase III clinical trials [6–9]. Results from post-hoc analysis of these trials showed an increased rate of serious adverse events (SAEs), poor tolerability and suboptimal rates of sustained virologic response (SVR) in patients with advanced hepatic fibrosis [6–12]. Few real-life studies evaluating safety and efficacy of triple therapy have been conducted on patients with advanced HCV-related liver disease [13–18]. Among them, the French multicenter early access program in treatment experienced patients with HCV-cirrhosis (ANRS CO20-CUPIC) [14] showed a high rate of severe complications and even mortality, while similar observations were made in other studies [15, 19–22]. The Early Access Program (EAP) HEP3002 was launched in 2011 in 16 nations, giving access to treatment with telaprevir to treatment-naive and experienced patients with bridging hepatic fibrosis and compensated cirrhosis due to HCV genotype 1 infection [13].

Transient elastography (TE), also known as FibroScan), is a validated non-invasive method to evaluate hepatic fibrosis in patients with chronic hepatitis C viral infection [23, 24] and has a high level of accuracy for detecting severe fibrosis and cirrhosis [25–27]. Furthermore, TE has a prognostic value in predicting severe portal hypertension, presence of oesophageal varices and occurrence of liver-related complications in patients with cirrhosis [25, 26, 28–30].

Studies of fibrosis evolution using non-invasive markers, during and after PR therapy in small cohorts of HCV-infected patients have been reported [31–41], but few studies investigated the utility of FibroScan as an SVR predictor. Among them, only two [32, 36] demonstrated that TE could provide information for the prediction of virological response prior to therapy. To our knowledge, no studies have evaluated the possible association between baseline FibroScan values and the occurrence of SAEs or liver-related complications during anti-HCV treatment, and no information regarding the use of TE in therapy guidance is available.

Here, we present the final results of EAP HEP3002 trial, in which about two thirds of the patients were initially evaluated for fibrosis stage using the FibroScan technology, representing a unique opportunity to explore FibroScan potential in the everyday clinical practice. Data from the HEP3002 trial were retrospectively analyzed to determine the clinical value of baseline FibroScan values for predicting safety and efficacy of telaprevir-based triple therapy in patients with advanced fibrosis or cirrhosis.

## Patients and Methods

### Study design

HEP3002 is an open-label EAP of HCV genotype 1 patients with advanced hepatic fibrosis or cirrhosis treated with telaprevir-based therapy (ClinicalTrials.gov NCT01508286) (S1 Protocol, S1 TREND Checklist). The program included patients from 16 centers across 181 sites in Europe, South America and Australasia and the enrolment criteria were similar to those of the REALIZE Phase III trial [9]. Patient recruitment and follow-up occurred between May 2011 and May 2014; the outline of the study has been described previously [13]. Informed written consent was obtained from each patient included in the study. The study protocol conforms to the ethical guidelines of the 1975 Declaration of Helsinki as reflected in a priori approval by the following institutions and human research committees: Therapeutic Goods Administration, Australia; Bundesamt für Sicherheit im Gesundheitswesen, Austra; Federaal Agentschap voor Geneesmiddelen en Gezondheidsproducten, Belgium; Agência Nacional de Vigilância Sanitária, Brazil; State Institute for Drug Control, Czech Republic; Bundesinstitut für Arzneimittel und Medizinprodukte, Germany, National Organization for Medicines, Greece, L’Agenzia Italiana del Farmaco, Italy; Ministry of Health, Luxembourg; Centrale Commissie voor Mensgebonden Onderzoek, The Netherlands; Medsafe, New Zealand; National Medicines Agency, Romania; Ministry of Health and Social Development of the Russian Federation, Russia; Ministry of Health, Serbia; State Institute for Drug Control, Slovakia; Agencia Española de Medicamentos y Productos Sanitarios, Spain and Swissmedic, Switzerland.

Patients were between 18 and 70 years and had documented severe liver fibrosis [Metavir F3 or Ishak 3–4 (S 3, 4)] or cirrhosis [Metavir F4 or Ishak 5, 6 (S5, 6)] due to HCV genotype 1 infection. In order to be included, patients had to have an absolute neutrophil count >1,500/mm³, platelets >90,000/mm³ and hemoglobin >12 g/dL for women and >13 g/dL for men. Key exclusion criteria included history or other evidence of decompensated liver disease, HCC, history of alcohol abuse, infection with HCV other than genotype 1 or co-infection with hepatitis B virus or human immunodeficiency virus. Both untreated patients and patients who failed to respond to a previous treatment were included, as long as they had not previously received an HCV protease or polymerase inhibitor. During the first 12 weeks of the program, all patients received 750 mg of TVR orally every 8 hours, in combination with PR. The type (α 2a versus α 2b pegylated interferon; Copegus versus Rebetol) and doses of PR were selected according to local guidelines. After 12 weeks of combination treatment, PR alone was administered for another 12 or 36 weeks depending on the virologic response to treatment and/or previous response type; detailed description of the treatment regimens has been reported [13]. Patients were assessed at weeks 4 and 12 to determine whether they met the predefined stopping rules based on virologic response. If at either time point HCV RNA levels were greater than 1,000 IU/mL, all treatment was permanently discontinued.

### Measures of disease severity and treatment efficacy

Hepatic fibrosis was assessed histologically or by non-invasive means. Liver specimens were obtained by percutaneous liver biopsy and fibrosis was staged using either the Metavir or the Ishak score. Patients who did not undergo a biopsy were evaluated by Fibrotest or FibroScan. The FibroScan cut-offs proposed by Castéra et al [42] were used to diagnose bridging fibrosis and cirrhosis. In this study the authors prospectively assessed the performance of FibroScan in HCV patients, compared to histology. The proposed cut-off values were determined from the distribution of stiffness values according to fibrosis stage for the maximum sum of sensitivity and specificity. The cut-offs for advanced fibrosis (F3 = numerous septa without cirrhosis) and cirrhosis (F4) were ≥9.5 kPa and ≥12.5 kPa respectively. The Child Pugh score system was used to define clinical status, with Child Pugh A stage indicating compensated liver disease.

The type of previous response to PR treatment was defined by standard criteria [9]. HCV RNA levels were measured using a range of assays at local investigational sites. The majority of sites used Roche COBAS TaqMan versions 1 or 2 with a lower limit of quantification (LLOQ) of 15–25 IU/mL and a lower limit of detection (LLOD) of 10 IU/mL, or Abbott RealTime assay with a LLOQ of 12 IU/mL and a LLOD of 10–12 IU/mL.

### Safety assessments

Data on safety issues were collected during the treatment period and follow-up, including laboratory assessments, physical examinations, evaluation of vital signs and the reporting of adverse events (AEs). AEs were graded by investigators using the DAIDS criteria [43] except for rash, for which protocol-specific guidance for grading and management was provided. Anemia was defined as an AE according to the following guidelines: grade 1 hemoglobin values between 10.0 and 10.9 g/dL or any decrease from baseline between 2.5 and 3.4 g/dL; grade 2 hemoglobin values between 9.0 and 9.9 g/dL or any decrease of hemoglobin between 3.5 and 4.4 g/dL; grade 3 hemoglobin values between 7.0 and 8.9 g/dL or any decrease of hemoglobin ≥4.5 g/dL; and grade 4 an hemoglobin value less than 7.0 g/dL. Anemia developed during treatment was managed by reduction of ribavirin doses according to label recommendations. Use of blood transfusions, erythropoetin (EPO) or iron-based products was allowed during the trial. Telaprevir dose reduction was prohibited, and if ribavirin dose modifications or discontinuation did not result in an improvement of hemoglobin levels, telaprevir was then discontinued. The detailed methodology of the safety assessments has been formerly described [13].

### Statistical methods

Since this program was designed to provide patients with early access to telaprevir and was not intended to evaluate a specific statistical hypothesis, all patients received open-label telaprevir in addition to PR. As such, no inferential statistical analyses were planned. Analyses were performed for descriptive purposes and were conducted using descriptive statistics to determine odds ratios along with 95% confidence intervals (CIs) by univariate and multivariate analyses.

In total, 2,034 patients were screened for the EAP, and 262 patients were excluded due to the study inclusion/exclusion criteria, withdrawal of consent or other reasons. The analysis was performed on the intent-to-treat set (N = 1,772), defined as all enrolled patients who received at least one dose of telaprevir. Patients with any major protocol deviation that plausibly affected efficacy or patients with HCV RNA under LLOQ at end-of-treatment and without any HCV RNA follow-up assessment within the week 24 follow-up visit window (and who did not relapse before) were excluded from the efficacy evaluable set.

For the analysis of safety data, FibroScan was tested for association with four adverse events: anemia (hemoglobin ≤10 g/dL), grade 1–4 infections, serious AEs and telaprevir discontinuation because of AEs. FibroScan results were divided into quartiles, and the number of events corresponding to each quartile are presented separately for the 4 endpoints.

The P values presented in these analyses were derived from the Wald Chi-square test for the univariate and multivariate predictors of SVR, the Chi-square test for the assessment of safety by Liver Stiffness Measurement and the univariate and multivariate predictors of anemia. The P values for the correlation between baseline FibroScan values and baseline laboratory parameters were derived from the Pearson Correlation Test.

Statistical analysis was performed using SAS® version 9.2 (SAS Institute Inc., Cary, NC, USA).

### End points

This study evaluated the usefulness of baseline FibroScan values for predicting SVR and the occurrence of anemia and other severe adverse events in HCV genotype 1 patients with advanced fibrosis (F3) or compensated cirrhosis (F4), treated with telaprevir-based triple therapy in the EAP.

## Results

### Patient characteristics

**Fig 1:**
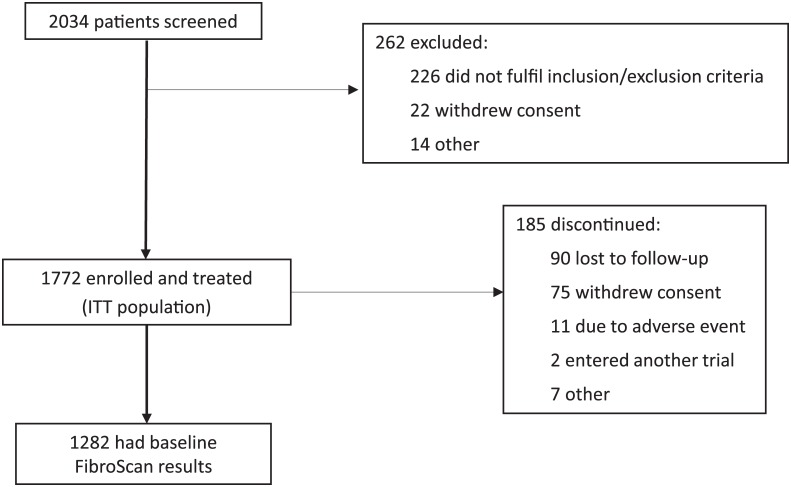
Patient disposition. Showing numbers of patients screened, enrolled and treated, and with baseline FibroScan results.

A total of 2,034 patients were screened and 1,772 were enrolled in the HEP3002 trial (Fig 1). Of these 1,772 patients, 1,282 (72%) had disease stage assessed by FibroScan and were included in the analyses. Among those who had liver fibrosis assessed by FibroScan 557 patients were classified as F3 (43%) at baseline and 724 (57%) as F4.

Demographics and baseline characteristics of the ITT population are shown in Table 1. Overall, the mean (SD) age of the patients was 53 (9.5) years; 1,121 patients (63%) were male and 1,741 (98%) were white. HCV genotype 1a was present in 379 patients (21%) and 1,172 (66%) had HCV RNA greater than or equal to 800,000 IU/mL at baseline. In total, 355 patients (20%) were treatment naive, 586 (33%) were prior treatment relapsers, 234 (13%) were prior partial responders, 495 (28%) were prior null responders and 49 (3%) had had a viral breakthrough. The response to prior therapy was unknown for one patient and 52 patients had had an unspecified previous non-response.

**Table 1:** Baseline and treatment characteristics of patients in the intent-to-treat population.

Characteristic*	Bridging fibrosis (N = 813)^†^	Cirrhosis (N = 959)	Overall (N = 1772)
Age year—mean (SD; range)	52 (10.1;22–73)	54 (8.8;19–75)	53 (9.5;19–75)
Body mass index (BMI)^‡^ —mean (SD)	26 (3.7)	27 (4.1)	27(4.0)
BMI—range	18–42	18–47	18–47
Males sex—no. (%)	494 (61)	627 (65)	1121 (63)
Race or ethnic group—no. (%)^§^			
White	801 (99)	940 (98)	1741 (98)
Black, Asian or other	12 (1)	19 (2)	31 (2)
HCV-1 subtype—no. (%)			
1a	173 (21)	206 (21)	379 (21)
1b	610 (75)	681 (71)	1291 (73)
Missing or unknown	30 (4)	72 (8)	102 (6)
HCV RNA log_10_—IU/mL^¶^	6.1±0.66	6.1±0.73	6.1±0.70
HCV RNA ≥800,000 IU/mL—no. (%)	545 (67)	627 (65)	1172 (66)
Model for End Stage Liver Disease score (range)	7.1 (6–14)	7.6 (6–20)	7.4 (6–20)
Liver stiffness measurement,—mean kPa (SD)	11.4 (2.82)	24.9 (12.66)	19.1 (11.78)
α-Fetoprotein—μg/L	8.8±11.2	16.5±25.0	13.0±20.3
Albumin—g/L	44.0±4.0	42.3±4.4	43.1±4.3
Bilirubin—μmol/L	12.7±6.4	14.7±7.1	13.7±6.8
Creatine—μmol/L	70.0±15.0	70.0±14.9	70.0±15.0
Glucose—mmol/L	5.5±1.6	5.9±2.0	5.7±1.8
Hemoglobin—g/L	150±13.8	149±13.3	150±13.5
Neutrophils–×10^9^/L	3.5±1.4	3.2±1.9	3.3±1.7
Platelets–×10^9^/L	190±57.9	154±54.4	171±58.9
Prothrombin intl. normalised ratio	1.03±0.10	1.08±0.13	1.06±0.12
IL28B genotype—no. (%)			
Missing or unknown	627 (77)	729 (76)	1356 (77)
CC	23 (3)	54 (6)	77 (4)
CT	127 (16)	129 (13)	256 (14)
TT	36 (4)	47 (5)	83 (5)
Previous type of response—no. (%)			
Total non-responders^††^	322 (40)	459 (48)	781 (44)
Prior null responder	198 (24)	297 (31)	495 (28)
Prior partial responder	103 (13)	131 (14)	234 (13)
Relapsers	288 (35)	298 (31)	586 (33)
Treatment naive	176 (22)	179 (19)	355 (20)
Viral breakthrough	26 (3)	23 (3)	49 (3)
Unknown	1 (0)	0 (0)	1 (0)

* Values are means±SD unless otherwise indicated. Percentages may not total 100 because of rounding.

^†^ Includes six F0–F2 patients.

^‡^ BMI is the weight in kilograms divided by the square of the height in meters.

^§^ Race or ethnic group was self-reported. Patients of any race could also identify themselves as Hispanic.

^¶^ Log_10_ values for HCV RNA are means±SE.

^††^ Includes prior null responders, prior partial responders and non-responders-unspecified.

Mean MELD score was 7.4 (1.4). In patients who had had liver fibrosis assessment by means of FibroScan, mean FibroScan result was 19.1 kPa. *IL28B* genotyping results were missing in the majority of patients. Of the 416 patients who had *IL28B* genotyping results, 77 (19%) had the *IL28B* genotype CC. The CT and TT genotypes were seen in 256 (62%) and 83 (20%) patients, respectively.

### Virologic response and correlation between FibroScan and SVR

In the ITT population, SVR24 was achieved by 70% (568/807) of patients with bridging fibrosis and 59% (566/959) of patients with cirrhosis (Table 2). In total, 78% (760/973) of patients who achieved eRVR went onto achieve SVR (Table 2).

**Table 2:** Efficacy outcome for each subgroup of patients with respect to fibrosis stage and to extended rapid virologic response (eRVR) in the intent-to-treat population.

n (%)	Bridging fibrosis (N = 813^†^)	Cirrhosis (N = 959)	Overall (N = 1772)
SVR	573 (70)	566 (59)	1139 (64)
Virologic failure			
Relapse	69 (9)	125 (13)	194 (11)
Viral breakthrough	79 (10)	138 (14)	217 (12)
Met a stopping rule	27 (3)	27 (3)	53 (3)
Other	1 (<1)	3 (<1)	4 (<1)
n/N (%)			
SVR in all patients who achieved eRVR,	394/468 (84)	366/505 (72)	760/973 (78)
SVR in patients who did not achieve eRVR	179/345 (52)	200/454 (44)	379/799 (47)

^†^ Includes six F0–F2 patients.

The percentage of treatment-naive patients who achieved SVR was 74% whereas, among treatment-experienced patients, 78% of treatment relapsers, 60% of partial responders, 44% of null responders achieved a cure (S1 Table).

Multiple parameters were tested for their association with SVR (Table 3). By univariate analysis, higher baseline FibroScan values, analyzed as a continuous log_10_ transformed variable, were predictive of lower SVR rates (Odds Ratio = 0.58, 95% CI = 0.46–0.74, p<0.0001). Other parameters significantly associated with a lower chance of SVR were HCV genotype 1a (p = 0.0003), baseline log_10_ alfa fetoprotein (p<0.0001), baseline log_10_ platelets (p<0.0001) and prior null response (p<0.0001). HCV genotype 1b, previous relapse or previous partial response were not predictive of SVR.

**Table 3:** Univariate and multivariate predictors of SVR.

	Univariate analysis			Multivariate analysis		
Factor	OR	95% CI	P value	OR	95% CI	P value
α-Fetoprotein (log_10_ μg/L)	0.20	0.15–0.28	<0.0001	0.28	0.19–0.39	<0.0001
Prior null response	0.29	0.22–0.37	<0.0001	0.34	0.26–0.44	<0.0001
Liver stiffness measurement >18 kPa	0.58	0.46–0.74	<0.0001	-	-	-
Genotype subtype 1a	0.60	0.45–0.79	0.0003	0.60	0.44–0.82	0.0012
Age ≤65 years	0.80	0.51–1.24	0.3114	0.60	0.37–0.98	0.0407
HCV RNA ≥800,000 UI/mL	0.83	0.64–1.07	0.1515	-	-	-
BMI (log_10_ kg/m^2^)	1.00	0.97–1.03	0.9176	-	-	-
Hemoglobin (log_10_ g/L)	1.01	1.00–1.02	0.0889	-	-	-
Initial dose of ribavirin	1.04	0.98–1.11	0.1905	-	-	-
Male sex	1.14	0.90–1.46	0.2808	-	-	-
PEG-IFN alfa 2a	1.21	0.85–1.72	0.2952	1.46	1.00–2.14	0.0522
Platelets (log_10_ n/L)	10.1	4.41–23.1	<0.0001	2.69	1.06–6.84	0.0373

All variables analysed in the univariate analysis were included in the initial multivariate model. Variables only remained in both models if they reached a significance level of the Wald chi-squared of 0.1 in the multivariate model. This analysis consisted of 1189 observations.

Multivariate analysis identified the following three independent predictors of lower SVR rates: genotype 1a (p = 0.0012), alfa fetoprotein (p<0.0001), log_10_ platelets (p = 0.0373), age (p = 0.0407) and prior null response (<0.0001). FibroScan was no longer statistically significant as an independent SVR predictor.

### Correlation between FibroScan and baseline laboratory parameters

FibroScan scores were tested for correlations with baseline laboratory parameters: platelets count, alfa fetoprotein, albumin, bilirubin and international normalized ratio (INR). All of the laboratory parameters tested were correlated with FibroScan, but the correlations were quite weak (correlation coefficients <0.4). More precisely, higher FibroScan score correlated with lower platelets and albumin and higher alfa fetoprotein, bilirubin and INR with a p value <0.0001 for each comparison.

### Safety

AEs observed in this study were consistent with telaprevir-based therapy known side effects, namely anemia (observed in 56% of patients), rash (30%), and pruritus (16%). As previously described, the most common serious side effect was, by far, anemia (26%). Overall, 12% of patients discontinued due to AEs, 4% due to rash and 3% due to anemia (Table 4).

**Table 4:** Reasons for discontinuation of telaprevir and incidence of the most common AEs and Grade 3–4 AEs during the telaprevir phase in the intent-to-treat population.

n (%)	Patients with bridging fibrosis*(N = 813)	Patients with cirrhosis (N = 959)	All patients(N = 1772)
Patients with at least one AE†	662 (81)	804 (84)	1466 (83)
Anaemia‡	435 (54)	562 (59)	997 (56)
Rash‡	230 (28)	305 (32)	535 (30)
Pruritus‡	114 (14)	163 (17)	277 (16)
Asthenia	74 (9)	80 (8)	154 (9)
Nausea	62 (8)	85 (9)	147 (8)
Thrombocytopenia	38 (5)	92 (10)	130 (7)
Anal Pruritus	36 (4)	64 (7)	100 (6)
Patients with at least one Grade 3 or 4 AE§	274 (34)	344 (36)	618 (35)
Anaemia	203 (25)	253 (26)	456 (26)
Rash	26 (3)	27 (3)	53 (3)
Asthenia	15 (2)	17 (2)	32 (2)
Thrombocytopenia	4 (<1)	25 (3)	29 (2)
Hemoglobin Decreased	9 (1)	9 (<1)	18 (1)
Neutropenia	5 (<1)	12 (1)	17 (1)
Reason for discontinuation¶			
Any AE	91 (11)	130 (14)	221 (12)
Rash	32 (4)	36 (4)	68 (4)
Anaemia	17 (2)	33 (3)	50 (3)
Asthenia	8 (1)	10 (1)	18 (1)
Vomiting	8 (1)	10 (1)	18 (1)
Nausea	7 (<1)	10 (1)	17 (1)
Pruritus	3 (<1)	11 (1)	14 (<1)
Abdominal Pain	1 (<1)	8 (<1)	9 (<1)
Infections	1 (<1)	5 (<1)	6 (<1)
Investigations	3 (<1)	3 (<1)	6 (<1)
Fatigue	3 (<1)	2 (<1)	5 (<1)
Pyrexia	2 (<1)	3 (<1)	5 (<1)
Thrombocytopenia	1 (<1)	4 (<1)	5 (<1)

* Includes six F0–F2 patients.

^†^ Listed are drug-related AEs that occurred in at least 5% of the overall population during the telaprevir phase.

^‡^ Included in this category are all related events that were described with a variety of descriptive terms.

^§^ Listed are grade 3–4 drug-related AEs that occurred in at least 1% of the overall population during the telaprevir phase.

^¶^ Listed are discontinuations that occurred in at least 5 patients. These figures are the number of patients who discontinued telaprevir; patients may have continued treatment with pegylated interferon plus ribavirin.

AE, adverse event.

The statistical analysis showed that higher FibroScan values were significantly correlated with anemia (p = 0.0365), occurrence of grade 1–4 infections (p = 0.0472) and SAEs (p<0.0001) but not with telaprevir discontinuation due to AEs (p = 0.0602) (Table 5).

**Table 5:** Safety based on Liver Stiffness Measurement in patients with baseline FibroScan values.

	Liver Stiffness Measurement (kPa)				P value
n/N (%)	**≤11.3**	**11.4–14.6**	**14.7–21.9**	**>21.9**	
Anemia (<10 g/dL)	140/321 (44)	132/296 (45)	164/315 (52)	161/306 (53)	0.0365
Grade 1–4 infections	6/334 (2)	7/307 (2)	13/322 (4)	17/318 (5)	0.0472
SAEs	26/334 (8)	19/307 (6)	47/322 (15)	53/318 (17)	<0.0001
Telaprevir discontinuation due to AE	35/334 (10)	34/307 (11)	54/322 (17)	46/318 (14)	0.0602

### FibroScan and the risk of anemia

Multiple parameters were tested for their association with anemia, defined as hemoglobin ≤10 g/dL and severe anemia defined as hemoglobin ≤8.5 g/dL (Table 6). In univariate analysis of anemia, among other parameters, the baseline FibroScan score was predictive of telaprevir-based treatment-related anemia (p = 0.0072). By multivariate stepwise logistic-regression analysis, independent predictors were older age (p<0.0001), female sex (p<0.0001), lower baseline hemoglobin (p<0.0001), and ribavirin dosing (p<0.0001), while FibroScan was no longer statistically significant.

**Table 6:** Univariate and multivariate predictors of anemia.

	Univariate analysis			Multivariate analysis		
Factor	OR	95% CI	P value	OR	95% CI	P value
**Predictors of anemia (hemoglobin <10 g/dL)** *						
Male sex	0.22	0.18–0.27	<0.0001	0.59	0.45–0.76	<0.0001
Platelets (log_10_ n/L)	0.25	0.13–0.48	<0.0001			
PEG-IFN alfa 2a	0.77	0.58–1.01	0.0572	-	-	-
Hemoglobin (log_10_ g/L)	0.92	0.91–0.93	<0.0001	0.94	0.93–0.95	<0.0001
BMI (log_10_ kg/m^2^)	0.96	0.94–0.98	0.0006	-	-	-
HCV RNA ≥800,000 UI/mL	1.07	0.87–1.31	0.5114	-	-	-
Prior null response	1.11	0.90–1.37	0.3416	-	-	-
Genotype subtype 1a	1.21	1.08–1.36	0.0011	-	-	-
Initial dose of ribavirin mg/kg	1.26	1.19–1.33	<0.0001	1.15	1.08–1.22	<0.0001
α-Fetoprotein (log_10_ μg/L)	1.75	1.38–2.21	<0.0001	-	-	-
Liver stiffness measurement (log_10_ kPa)	2.03	1.21–3.40	0.0072	-	-	-
Age >65 years	3.27	2.20–4.87	<0.0001	2.62	1.70–4.03	<0.0001
**Predictors of severe anemia (hemoglobin <8.5 g/dL)** *						
Platelets (log_10_ n/L)	0.21	0.09–0.48	0.0002	-	-	-
Male sex	0.31	0.24–0.41	<0.0001	0.70	0.51–0.95	0.0224
Prior null response	0.82	0.61–1.08	0.1604	-	-	-
HCV RNA ≥800,000 UI/mL	0.86	0.66–1.11	0.2423	-	-	-
PEG-IFN alfa 2a	0.93	0.65–1.32	0.6739	-	-	-
Hemoglobin (log_10_ g/L)	0.95	0.94–0.96	<0.0001	0.96	0.94–0.97	<0.0001
BMI (log_10_ kg/m^2^)	0.97	0.94–1.00	0.0424	-	-	-
Genotype subtype 1a	1.20	1.02–1.41	0.0237	-	-	-
Initial dose of ribavirin mg/kg	1.22	1.14–1.30	<0.0001	1.12	1.04–1.20	0.0020
α-Fetoprotein (log_10_ μg/L)	1.92	1.43–2.59	<0.0001	1.67	1.22–2.27	0.0012
Liver stiffness measurement (log_10_ kPa)	2.54	1.32–4.87	0.0051	-	-	-
Age >65 years	2.81	1.93–4.09	<0.0001	2.25	1.51–3.37	<0.0001

* N>1700 except for assessment of liver stiffness measurement (N = 1240) and α-Fetoprotein (N = 1679)

This analysis consisted of 1671 observations.

In univariate analysis of severe anemia, among other parameters, the baseline FibroScan score was predictive of telaprevir-based treatment related anemia (p = 0.0051). By multivariate stepwise logistic-regression analysis, independent predictors were older age (p<0.0001), lower baseline hemoglobin (p<0.0001), ribavirin dosing (p = 0.0020), alfa fetoprotein (p = 0.0012), and female sex (p = 0.0224).

## Discussion

In this paper we present the final results of the international telaprevir EAP. Of the 1,772 patients with either bridging fibrosis or cirrhosis resulting from HCV genotype 1 infection who were treated with telaprevir-based triple therapy, a percentage of 64% achieved SVR. These results are similar to the ones of an interim analysis of the study, published recently by Colombo et al [44].

Further analyzing the data from this large, multicenter study, we evaluated the clinical interest of FibroScan baseline values in the prediction of efficacy and safety of telaprevir-based triple therapy in patients with HCV genotype 1-related, advanced liver disease. The studied population represents the 72% of the patients included in the HEP3002 EAP and who were initially evaluated for the fibrosis stage with FibroScan. Our cohort included both treatment-naive and -experienced patients with bridging hepatic fibrosis and compensated cirrhosis, a population with priority to treatment but poorly represented in clinical trials. The present study suggests that the baseline FibroScan value is a predictor of efficacy and safety of telaprevir-based treatment. Higher baseline FibroScan measurements are correlated with lower SVR rates and higher incidence of anemia in univariate analyses. In multivariate analyses this correlation is not strong enough to be considered significant and was overshadowed by other more accurate clinical and biologic independent predictors. Additionally, higher baseline FibroScan values were correlated with the occurrence of infections and SAEs.

To our knowledge, there are only 2 previous studies validating the predictive power of FibroScan for long-term antiviral treatment response [32, 36]. Patel et al evaluated the diagnostic utility of baseline FibroScan in 214 treatment-naive patients infected by genotype 1 to 3 of HCV in whom liver fibrosis was staged by a percutaneous liver biopsy and classified by Metavir (F0–F4) and who underwent treatment with interferon and ribavirin. These authors demonstrated lower baseline FibroScan scores in patients who achieved an SVR than in those who failed therapy, indicating that FibroScan could provide useful adjunctive information for the prediction of virologic response prior to IFN-based therapy for chronic HCV. The second study by Stasi et al evaluated 74 treatment naive, genotype 1 to 4 chronic HCV patients at various fibrosis stages, who were treated with PR. Patients with pretreatment FibroScan values >12 kPa that are predictive of advanced fibrosis in HCV patients, had a significantly lower response to antiviral therapy suggesting that FibroScan could be used for pretreatment patient stratification to optimize patient selection to antiviral therapy. Along this line was a smaller French study where baseline FibroScan was tested for the prediction of SVR to triple therapy with either boceprevir or telaprevir in 125 HCV genotype 1-infected patients with advanced fibrosis or cirrhosis, who were previously unsuccessfully treated with PR [45]. In that study, a pre-treatment FibroScan cut-off of 21.3 kPa was discriminant for the occurrence of SVR. Other smaller studies, however, have failed to demonstrate similar association between pretreatment FibroScan results and a likelihood to respond to interferon based regimens [31, 33–35]. As far as safety is concerned, to our knowledge, there are no clinical trials evaluating pretreatment FibroScan to predict occurrence of treatment related AEs.

In this sub-analysis of the EAP HEP3002 cohort, we showed the existence of a correlation between the baseline FibroScan results and the safety and efficacy of the telaprevir-based triple therapy in more than 1,000 patients treated in field practice. The strength of our study (apart from the relevant sample size and the use of a potent anti HCV regimen) was also the exclusive inclusion of both treatment-naive and treatment-experienced patients with advanced liver disease. This is in fact a most difficult-to-treat population in the everyday clinical practice, as advanced hepatic fibrosis in chronic HCV infection represents a prioritization criterion for therapy but also a tolerability and safety issue in patients exposed to triple therapy [6, 7, 24, 46].

FibroScan has gained popularity in HCV field since it has been extensively validated for the diagnosis of disease severity [23, 24, 35, 47–53] and prediction of liver related mortality [29, 30, 52–55]. Despite the rapid evolution in the field of HCV treatment, PIs in association with PR remain a standard of care in accurately selected genotype 1-infected patients in countries where all oral anti-HCV regimens are not available or are restricted for money constraints. However, since triple therapy requires expertise to prevent AEs in fragile patient populations like cirrhotics, in real life practice the disposing of reliable markers to predict safety and efficacy through the selection of ideal patients to treat is crucial. In our study, higher pre-treatment FibroScan scores correlated with a higher risk of anemia in univariate analysis and with a lower chance of SVR to telaprevir-based triple therapy. The latter, however, was independently predicted by genotype 1b, elevated alfa fetoprotein and prior null response. On the other hand, anemia, was independently predicted by older age, female sex, low baseline hemoglobin, higher weight-based ribavirin dosing and high baseline HCV RNA.

The EAP study as every real-life study has some limitations. Six patients with a baseline FibroScan <9.5 kPa, were considered protocol violations, and therefore were excluded from the analyses. The FibroScan cut-offs used for advanced fibrosis and cirrhosis were based on a previous study [42], but its performance for the assessment of fibrosis in previously treated HCV patients has not been investigated. Moreover, we could not review all FibroScan protocols of the participating centers but we only captured the elastography result without the companion reliability results (i.e. IQR) and success rates of each exam. In the overall EAP cohort, 12% of patients experienced SAEs that ultimately led to telaprevir discontinuation, a relatively low number compared to the 40% of SAEs in the CUPIC trial, further confirming the exclusion in the EAP study of high-risk patients with poorly compensated liver and bone marrow that were instead enrolled in the French study [14].

In conclusion, while FibroScan on its own was able to predict both efficacy and safety of triple therapy in patients with advanced fibrosis, in the frame of overall clinical assessment of HCV patients it is overshadowed as a predictor of safety and efficacy of telaprevir-based triple therapy by other clinical and biological parameters.

## Supporting Information

S1 Protocol Clinical Trial Protocol. (PDF)

S1 Table Efficacy outcome for each subgroup of patients with respect to previous PR treatment and to extended rapid virologic response (eRVR) in the intent-to-treat population. (DOCX)

S1 TREND Checklist TREND Checklist. (DOCX)
