# Supplementary material for: Final Results of the Telaprevir Access Program: FibroScan Values Predict Safety and Efficacy in Hepatitis C Patients with Advanced Fibrosis or Cirrhosis
Source: PLoS One. 2015 Sep 23;10(9):e0138503. doi: 10.1371/journal.pone.0138503 (PMC4580464; doi:10.1371/journal.pone.0138503)
Supplement: S1 Protocol — (PDF) [file pone.0138503.s001.pdf]

Clinical Trial Protocol

**Final Version:** FINAL / 11 Jan 2011	**Drug: Telaprevir**<br>(VX-950, TVR)
**Clinical Phase: Not Applicable**	
**Title:**	Multicenter, Open-Label, Early Access Program of Telaprevir in Combination With Peginterferon Alfa and Ribavirin in Genotype 1 Chronic Hepatitis C Subjects With Severe Fibrosis and Compensated Cirrhosis
**Summary:**	A multicenter, multinational, open-label, non-comparative early access program designed to provide telaprevir to subjects who are expected to benefit from telaprevir-based therapy and to collect additional safety and tolerability data on telaprevir treatment in combination with peginterferon alfa (Peg-IFN-alfa) and ribavirin (RBV). Eligible subjects include men and women, between 18 and 70 years of age, inclusive, with hepatitis C virus (HCV) infection genotype 1, a quantifiable serum HCV RNA, documentation of liver fibrosis, and compensated liver disease (Child Pugh Grade A). Subjects will be excluded if they are eligible for enrollment into an ongoing clinical study of telaprevir.<br><br>All eligible subjects will receive telaprevir 750 mg every 8 hours during 12 weeks in combination with standard-of-care (Peg-IFN-alfa/RBV). Subjects will subsequently be treated with Peg-IFN-alfa/RBV for either an additional 12 or 36 weeks based on virologic response to treatment and/or by type of subject (treatment naïve or prior treatment relapsers, or previously treated with prior partial or prior null response, or who had viral breakthrough; and Metavir/Ishak score). Virologic response will be measured throughout the study by plasma HCV RNA levels.
**Trial Location:**	International, multicenter
**Sponsor:**	JANSSEN*<br>*Medical Affairs*<br>Turnhoutseweg 30<br>B-2340 Beerse, Belgium

*JANSSEN is a global organization that operates through different legal entities in various countries. Therefore, the legal entity acting as the sponsor for studies of JANSSEN may vary, such as, but not limited to Janssen Pharmaceutica NV or Janssen-Cilag International NV. The term "sponsor" or "designee" is used throughout the protocol to represent these various legal entities that have been identified to perform various clinical trial services; the actual sponsor or designee is identified on the Contact Information page(s) that accompanies this protocol as a separate document.

Compliance: This study will be conducted in compliance with this protocol, Good Clinical Practice, the Declaration of Helsinki, and applicable regulatory requirements.

Confidentiality Statement

The information in this document contains trade secrets and commercial information that are privileged or confidential and may not be disclosed unless such disclosure is required by applicable law or regulations. In any event, persons to whom the information is disclosed must be informed that the information is *privileged* or *confidential* and may not be further disclosed by them. These restrictions on disclosure will apply equally to *all* future information supplied to you that is indicated as *privileged* or *confidential*.

TABLE OF CONTENTS

SYNOPSIS **5**

TIME AND EVENTS SCHEDULE **9**

ABBREVIATIONS **11**

1. INTRODUCTION **12**
1.1. Background 12
1.2. Overall Rationale for the Study 17

2. OBJECTIVES **17**

3. OVERVIEW OF STUDY DESIGN **17**
3.1. Study Design 17
3.2. Study Design Rationale 19
3.2.1. Study Population 19
3.2.2. Telaprevir Dose 20
3.2.3. Telaprevir Duration 20
3.2.4. Peg-IFN-Alfa and RBV Duration 21
3.2.5. Treatment Modifications Based on Virologic Response 21

4. SUBJECT SELECTION **22**
4.1. Inclusion Criteria 22
4.2. Exclusion Criteria 23
4.3. Prohibitions and Restrictions 25

5. TREATMENT ALLOCATION **26**

6. DOSAGE AND ADMINISTRATION **26**

7. TREATMENT COMPLIANCE **28**

8. PRESTUDY AND CONCOMITANT THERAPY **28**

9. STUDY EVALUATIONS **29**
9.1. Study Procedures 29
9.1.1. Overview 29
9.1.2. Screening Phase 30
9.1.3. Open-Label Treatment Phase 30
9.1.4. Posttreatment Phase (Follow-Up) 30
9.2. Efficacy Evaluations 31
9.3. Safety Evaluations 31
9.3.1. Specific Toxicities 33
9.3.1.1. Assessment, Treatment, Management, and Documentation of Rash 33
9.3.1.1.1. Rash Assessment: Severity Grading 33
9.3.1.1.2. Rash Treatment 34
9.3.1.1.3. Rash Management 35
9.3.1.2. Management of Anemia 38

10. SUBJECT COMPLETION/WITHDRAWAL **39**
10.1. Completion 39
10.2. Discontinuation of Treatment 39
10.3. Withdrawal From the Study 41

11. STATISTICAL METHODS **41**
11.1. Analysis Population 41

11.2. Subject Information 41
11.3. Efficacy Analyses 42
11.4. Sample Size Determination 42
11.5. Safety Analyses 42

12. ADVERSE EVENT REPORTING 43
12.1. Definitions 43
12.1.1. Adverse Event Definitions and Classifications 43
12.1.2. Attribution Definitions 45
12.2. Special Reporting Situations 45
12.2.1. Severity Criteria 45
12.3. Procedures 45
12.3.1. All Adverse Events 45
12.3.2. Serious Adverse Events 47
12.3.3. Pregnancy 48
12.4. Contacting Sponsor Regarding Safety 48

13. PRODUCT QUALITY COMPLAINT HANDLING 48
13.1. Procedures 49
13.2. Contacting Sponsor Regarding Product Quality 49

14. STUDY DRUG INFORMATION 49
14.1. Physical Description of Study Drug(s) 49
14.2. Packaging 49
14.3. Labeling 50
14.4. Preparation, Handling, and Storage 50
14.5. Drug Accountability 50

15. STUDY-SPECIFIC MATERIALS 51

16. ETHICAL ASPECTS 51
16.1. Study-Specific Design Considerations 51
16.2. Regulatory Ethics Compliance 51
16.2.1. Investigator Responsibilities 51
16.2.2. Independent Ethics Committee or Institutional Review Board 52
16.2.3. Informed Consent 53
16.2.4. Privacy of Personal Data 54
16.2.5. Country Selection 55

17. ADMINISTRATIVE REQUIREMENTS 55
17.1. Protocol Amendments 55
17.2. Regulatory Documentation 55
17.2.1. Regulatory Approval/Notification 55
17.2.2. Required Prestudy Documentation 56
17.3. Subject Identification, Enrollment, and Screening Logs 56
17.4. Source Documentation 57
17.5. Case Report Form Completion 57
17.6. Data Quality Assurance/Quality Control 58
17.7. Record Retention 58
17.8. Monitoring 59
17.9. Study Completion/Termination 60
17.9.1. Study Completion 60
17.9.2. Study Termination 60
17.10. On-Site Audits 60
17.11. Use of Information and Publication 61

REFERENCES 63

Attachment 1: Child-Pugh Classification of Severity of Liver Disease 65
Attachment 2: Interactions and Dose Recommendations With Other Medicinal Products ... 66
Attachment 3: DAIDS Table... 72

LAST PAGE **91**

SYNOPSIS

Multicenter, Open-Label, Early Access Program of Telaprevir in Combination With Peginterferon Alfa and Ribavirin in Genotype 1 Chronic Hepatitis C Subjects With Severe Fibrosis and Compensated Cirrhosis

Telaprevir (VX-950, TVR), which is being developed by Vertex Pharmaceuticals Incorporated (Vertex) in collaboration with the Johnson & Johnson companies, Janssen Pharmaceutica NV and Tibotec BVBA. Telaprevir is a member of a new class of drugs being developed for chronic hepatitis C: Direct Acting Antiviral (DAA) agents. Unlike pegylated interferon (Peg-IFN) and ribavirin (RBV), DAA agents act directly on the hepatitis C virus (HCV) replication cycle. Telaprevir is a specific, reversible, covalent, tight- and slow-binding NS3•4A inhibitor that was derived through structure-based drug design. Telaprevir prevents HCV replication by inhibiting the HCV NS3•4A protease, which is an enzyme that is essential for HCV replication.

OBJECTIVE

The objectives of this early access program are to provide telaprevir for subjects with genotype 1 chronic hepatitis C with severe fibrosis and compensated cirrhosis who reside in countries in which telaprevir is not yet commercially available and who are not eligible for enrollment into an ongoing clinical study of telaprevir, and to collect additional safety and tolerability data on telaprevir treatment in combination with Peg-IFN-alfa and RBV.

Hypothesis

In this early access program, all subjects will receive open-label telaprevir in addition to standard-of-care treatments. Only selected safety and tolerability data will be collected. As such, no formal statistical hypothesis will be tested.

OVERVIEW OF STUDY DESIGN

This is a multicenter, multinational, open-label, non-comparative early access program designed to provide telaprevir to subjects who are expected to benefit from telaprevir-based therapy and to collect additional safety and tolerability data on telaprevir treatment in combination with Peg-IFN-alfa and RBV. Enrollment of subjects into this early access program will continue until telaprevir becomes available for reimbursement in the country in which a subject resides, or until September 2013, whichever occurs first, unless other guidelines applicable per local regulations. Subjects already enrolled in the early access program when enrollment is stopped should complete treatment (ie, telaprevir, Peg-IFN-alfa/RBV) and continue follow-up according to the protocol schedule.

Approximately 3,000 subjects in countries in which telaprevir is not yet commercially available and who meet all of the inclusion and none of the exclusion criteria are expected to be enrolled. All subjects must sign informed consent prior to the conduct of any procedures for the early access program.

All subjects eligible to enter the study will receive telaprevir 750 mg every 8 hours (q8h) during the first 12 weeks of the early access program in combination with standard-of-care (Peg-IFN-alfa/RBV). The duration of Peg-IFN-alfa/RBV will be as follows:

- Subjects with severe fibrosis (Metavir F3 or Ishak 3-4) who are treatment naïve or prior treatment relapsers will subsequently be treated with Peg-IFN-alfa/RBV for either an additional 12 or 36 weeks (total treatment duration of 24 or 48 weeks, respectively) based on virologic response to treatment as measured by the Week 4 and 12 plasma HCV RNA levels reported by the local laboratory:
  - Subjects with undetectable HCV RNA at Weeks 4 and 12 will receive an additional 12 weeks of Peg-IFN-alfa/RBV alone for a total treatment duration of 24 weeks.
  - Subjects with detectable HCV RNA at either Week 4 or 12 will receive an additional 36 weeks of Peg-IFN-alfa/RBV alone for a total treatment duration of 48 weeks; see stopping rules for further guidelines.

- Previously treated subjects with prior partial or prior null response, or who had viral breakthrough, with severe fibrosis (Metavir F3 or Ishak 3-4) and all subjects with cirrhosis (Metavir F4 or Ishak 5-6) will subsequently be treated with Peg-IFN-alfa/RBV alone for an additional 36 weeks (total treatment duration of 48 weeks); see stopping rules for further guidelines.

For all subjects, stopping rules based on virologic response (ie, HCV RNA levels) will be applied to ensure that telaprevir or Peg-IFN-alfa/RBV treatments are stopped if subjects have viral breakthrough or failure. At Weeks 4 and 12, the stopping rules presented in the table below will be applied. For guidelines for treatment discontinuation after Week 12 of treatment, refer to the local prescribing information for Peg-IFN-alfa and RBV.

Guidelines for Discontinuation of Telaprevir, Peg-IFN-alfa and RBV Treatment

Medicinal product(s)	> 100 IU/mL at Week 4 of treatment	> 100 IU/mL at Week 12 of treatment
Telaprevir	Permanently discontinue	Telaprevir treatment completed
Peg-IFN-alfa/RBV	Continue to Week 12 decision point	Permanently discontinue

Additional plasma HCV RNA levels should be measured at Week 24 for subjects with a total treatment duration of 24 weeks and at Weeks 24 and 48 for subjects with a total treatment duration of 48 weeks. Additionally, all subjects should have a follow-up visit, including measurement of plasma HCV RNA levels, 24 weeks after the last administered dose of any treatment (ie, telaprevir, Peg-IFN-alfa, or RBV) (eg, Week 48 for subjects receiving 24 weeks of treatment, Week 72 for subjects requiring 48 weeks of treatment).

Subjects who discontinue all treatments (ie, telaprevir, Peg-IFN-alfa, and RBV) early or withdraw early from the early access program should have an early treatment discontinuation/early withdrawal visit performed and a follow-up visit performed 24 weeks after the last administered dose of treatment, unless informed consent for further follow-up has been withdrawn. Plasma HCV RNA levels should be measured at each of these visits.

Recommended evaluations of safety and tolerability to be performed during the early access program are presented in the Time and Events Schedule that follows the Synopsis.

A diagram of the early access program is provided below.

Schematic Overview of the Early Access Program

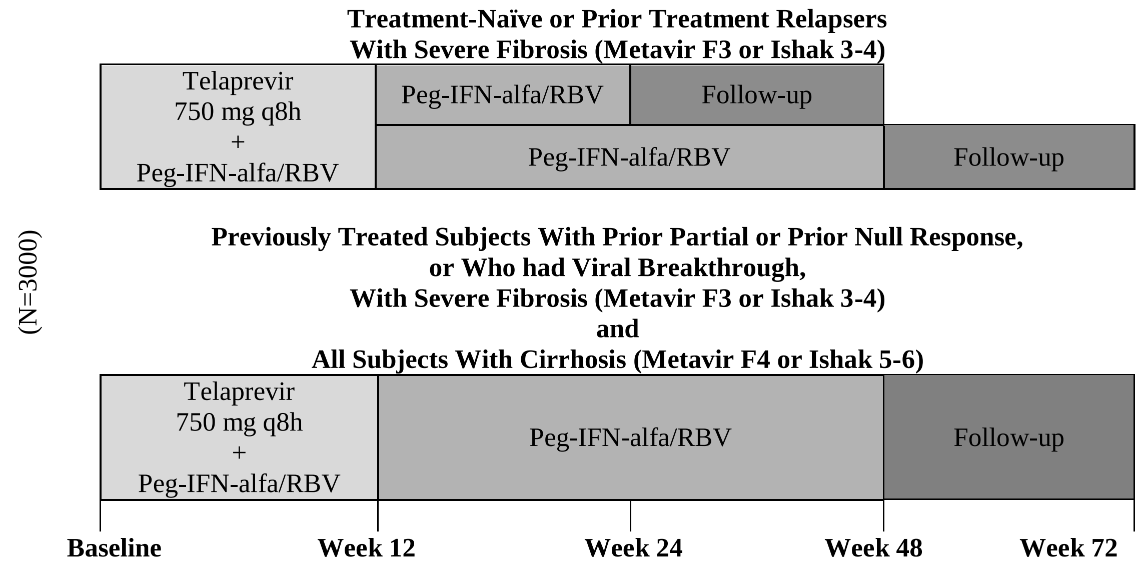

Peg-IFN-alfa/RBV=pegylated interferon alfa and ribavirin; q8h=every 8 hours.

SUBJECT SELECTION

Eligible subjects include men and women, between 18 and 70 years of age, inclusive, with HCV infection genotype 1, a quantifiable serum HCV RNA, documentation of liver fibrosis assessed by liver biopsy or non-invasive test (eg, fibrotest, fibroscan) showing severe fibrosis (Metavir F3 or Ishak 3-4) or cirrhosis (Metavir F4 or Ishak 5-6), and compensated liver disease (Child-Pugh Grade A). Additionally subjects must have access to the Hepatitis C standard-of-care (Peg-IFN-alfa/RBV) to participate in this program. Subjects will be excluded if they are eligible for enrollment into an ongoing clinical study of telaprevir, are infected or co-infected with HCV of another genotype than genotype 1, have a history of receiving investigational HCV protease or polymerase inhibitors, have signs or symptoms of hepatocellular carcinoma (HCC), have a history or other evidence of decompensated liver disease, or have co-infection with active hepatitis B or human immunodeficiency virus (HIV).

DOSAGE AND ADMINISTRATION

All subjects will receive telaprevir 750 mg (two 375 mg film-coated tablets) orally q8h with food (the total daily dose is 6 tablets [2,250 mg]) for 12 weeks in combination with standard-of-care consisting of Peg-IFN-alfa and RBV, as described in Overview of Study Design. Subjects should be instructed to swallow the tablets whole (eg, subjects should not chew, break, or dissolve the tablet).

Dose modifications of telaprevir are prohibited and once telaprevir treatment is discontinued for safety reasons or inadequate virologic response, it may not be reinitiated.

Concomitant administration of Peg-IFN-alfa and RBV will follow label recommendations of each product and the duration of therapy with these drugs will vary by early virologic response and/or by type of subject (treatment naïve or prior treatment relapsers, or previously treated with prior partial or prior null response, or who had viral breakthrough; and Metavir/Ishak score). In previously treated subjects, when available, the use of Peg-IFN-alfa-2a in combination with telaprevir and RBV should be considered due to the limited data with Peg-IFN-alfa-2b. To maximize adherence to dosing regimens and minimize inconvenience for subjects, RBV, which is taken twice daily, may be taken at the same time as the morning and evening telaprevir doses (rather than every 12 hours [q12h]). If the number of daily RBV tablets is an odd number, the larger number of tablets should be taken with the morning dose (ie, 3 tablets in the morning and 2 at night, so the larger dose is given before the longer period between doses). If RBV-related insomnia is a concern, then the second dose of RBV may be administered with the afternoon telaprevir dose instead of the evening dose.

EFFICACY EVALUATIONS

Although there are no formal efficacy evaluations in this early access program, plasma HCV RNA levels will be determined by the local laboratory and results will be used as the basis for treatment-guided therapy as described in Overview of Study Design.

SAFETY EVALUATIONS

The Division of AIDS Table for Grading the Severity of Adult and Pediatric Adverse Events (DAIDS) criteria will be used to grade adverse events except for rash which has protocol-specific definitions of severity grades.

The following adverse events which occur between the first use of investigational product (telaprevir) and 30 days after administration of the last dose of telaprevir will be recorded in the case report form (CRF):

- Adverse events of special interest
  - All rash and anemia events, regardless of DAIDS grade or relationship to telaprevir
- The following which are considered <u>at least possibly related to telaprevir</u> by the investigator
  - All adverse events, including laboratory abnormalities, with DAIDS grade 2 (moderate) or higher
  - Other adverse events (ie, DAIDS grade 1 [mild]), including laboratory abnormalities, considered medically significant (eg, require medical intervention)
- All adverse events leading to discontinuation of telaprevir, regardless of DAIDS grade or relationship to telaprevir

- All serious adverse events, regardless of relationship to telaprevir
- Any pregnancy in female subjects or in female partners of male subjects
- Special reporting situations: overdose, suspected abuse/misuse, inadvertent or accidental exposure, or medication error of telaprevir
- Other adverse events will be recorded as per local regulations only

A product quality complaint (PQC) is defined as any suspicion of a product defect related to manufacturing, labeling, or packaging of telaprevir. All initial PQCs must be reported to the sponsor by the investigational staff as soon as possible after being made aware of the event. If the defect is combined with a serious adverse event, the investigational staff must report the PQC to the sponsor according to the serious adverse event reporting timelines.

STATISTICAL METHODS

No formal sample size calculation has been performed. The trial is not designed to evaluate a specific statistical hypothesis but to provide subjects with early access to telaprevir. Therefore, the number of enrolled subjects is not based on statistical but rather practical considerations involving the expected prevalence of HCV infection in the included countries and the projected number of eligible subjects based on major eligibility criteria along with other available therapies.

The intent-to-treat analysis population will be defined as all enrolled subjects who received at least 1 dose of telaprevir. The intent-to-treat analysis population will be used for all statistical analyses, unless otherwise specified.

Safety analyses will be performed for descriptive purposes only. There will be no inferential statistical analyses. Although there is no control arm and statistical comparisons are not formally planned, multiple logistic regression and subgroup analyses may be used to assess the variability of selected safety parameters across potential prognostic factors including, but not limited to, demographic, treatment, and other baseline characteristics. These will be viewed as hypothesis-generating rather than hypothesis testing analyses. These methods will be described in the Statistical Analysis Plan which will be finalized prior to database lock.

There are no planned interim analyses of safety data. However, the safety data may be used for standard safety reporting required for health authority submissions or requests.

TIME AND EVENTS SCHEDULE

Phase	Screening	Baseline	Open-Label Treatment							Posttreatment
**Procedures** / **Visit**	**Week -6**[a]	**Day 1**[a,b]	**Week 2**[c]	**Week 4**	**Week 8**	**Week 12**	**Week 24**[c,d]	**Week 48**[c,d]	**Early Treatment Discontinuation/ Early Withdrawal**	**Follow-up visit (24 weeks after last administered dose of treatment)**
Informed consent	X									
Medical and surgical history and concomitant diseases[e,f]	X									
HCV genotype determination[g]	X									
Demographic data	X									
Pregnancy test[h]	X[i]	X[i]		X[j]	X[j]	X[j]	X[j]	X[j]	X[j]	X[j]
Inclusion/exclusion criteria	X	X								
Electrocardiogram (recommended)	X									
Serology: HIV antibody, HbsAg[g]	X[k]									
Thyroid-stimulating hormone (TSH)[g]	X			X	X	X[c]	X[c]	X[c]		X[c]
Height	X									
Physical examination,[f,l] weight	X	X	X	X	X	X	X	X	X	
Pulse, blood pressure	X	X	X	X	X	X	X	X	X	
Hematology, serum chemistry[f,g,m]	X	X	X	X	X	X	X	X	X	
HCV RNA[g]	X			X		X	X	X	X[c]	X[n]
Dispense telaprevir		X		X	X					
Telaprevir accountability[o]			X	X	X	X			X	
Prior and concomitant hepatitis C therapy	X	X	X	X	X	X	X	X	X	X
Concomitant therapy for rash, anemia, serious adverse events[p]		X	X	X	X	X			X	
Serious adverse events[q]	X[r]	X[r]	X	X	X	X	X[r]	X[r]	X[r]	X[r]
Adverse events[s,t]	X[r]	X[r]	X	X	X	X	X[r]	X[r]	X[r]	X[r]

a The time period between the screening visit and the baseline visit (Day 1) can be reduced provided all data are available to perform the baseline visit.

b All assessments are predose on Day 1.

c Recommended time points or according to local regulations, as per standard-of-care.

d The total duration of Peg-IFN-alfa/RBV treatment is 48 weeks in previously treated subjects with prior partial or prior null response, or who had viral breakthrough, with severe fibrosis (Metavir F3 or Ishak 3-4) and all subjects with cirrhosis (Metavir F4 or Ishak 5-6). Subjects with severe fibrosis (Metavir F3 or Ishak 3-4) who are treatment naïve or prior treatment relapsers with undetectable HCV RNA at Week 4 and 12 should receive a total Peg-IFN-alfa/RBV treatment duration of 24 weeks and with detectable HCV RNA at Week 4 or 12 must receive a total Peg-IFN-alfa/RBV treatment duration of 48 weeks. Further, for all subjects, stopping rules will be applied: subjects with HCV RNA >100 IU/mL at Week 4 should discontinue telaprevir and continue with both Peg-IFN-alfa and RBV and subjects with HCV RNA >100 IU/mL at Week 12 should discontinue Peg-IFN-alfa and RBV. For guidelines for treatment discontinuation after Week 12 of treatment, refer to the local prescribing information for Peg-IFN-alfa and RBV.

(Continued)

[e] Clinically significant medical history. Subject must have documentation of liver fibrosis assessed by liver biopsy or non-invasive test (eg, fibrotest, fibroscan); for subjects with Metavir F3 or Ishak 3-4, the liver biopsy or non-invasive test should have been performed within the past 18 months. Serum alpha-fetoprotein (AFP) level and ultrasonography should be available at screening for all subjects to screen for hepatocellular carcinoma (both tests should have been done a maximum of 4 months before the screening). In case of abnormalities, appropriate procedures according to clinical practice should be taken.

[f] Note that it is the responsibility of the investigator to ensure that according to standard-of-care the subject's medical history or laboratory values do not preclude treatment according to the respective local prescribing information for Peg-IFN-alfa or RBV. Additionally it is also the responsibility of the investigator to perform follow-up physical examinations and clinical laboratory tests according to the respective local prescribing information for Peg-IFN-alfa or RBV.

[g] Local laboratory assessment.

[h] Pregnancy test for women of childbearing potential only. For male subjects with female partners of childbearing potential, pregnancy tests are strongly recommended. Male subjects whose partners are pregnant must be instructed to use a condom to minimize exposure of RBV to the partner. Female subjects of childbearing potential and their male partners must use 2 effective contraceptive methods during treatment and for 4 months after all treatment has ended. Male subjects and their female partners of childbearing potential must use 2 effective contraceptive methods during treatment and for 7 months after all treatment has ended.

[i] Serum (β-HCG) pregnancy test at screening and serum or urine pregnancy test before first dose of treatment to ensure that women are not pregnant at the time of starting treatment.

[j] For female subjects of childbearing potential, pregnancy testing (serum or urine) should occur monthly during telaprevir combination therapy and for 4 months after all treatment has ended. For male subjects with female partners of childbearing potential, pregnancy testing should occur monthly during telaprevir combination therapy and for 7 months after RBV therapy has ended.

[k] If not performed in the past or subject to change at screening.

[l] At screening a complete physical examination should be performed. At all other visits up to 30 days after the last dose of telaprevir, physical examinations should be directed at detecting adverse events. All physical examinations performed more than 30 days after the last dose of telaprevir should be completed according to clinical practice.

[m] Electrolyte disturbances (eg, hypokalemia, hypomagnesemia, and hypocalcemia) should be monitored and corrected, if necessary, before initiation and during telaprevir therapy.

[n] A posttreatment HCV RNA level that is measured at a time point at least 12 weeks after the last dose of treatment may be reported if HCV RNA is not measured at 24 weeks after the last dose of treatment.

[o] Drug accountability only for telaprevir.

[p] Collect all concomitant therapies for rash and anemia, regardless of adverse event severity grade, and all concomitant therapies for serious adverse events through 30 days after last dose of telaprevir.

[q] Serious adverse events, as described in the protocol, will be recorded in the CRF starting from the first use of telaprevir until 30 days after administration of the last dose of telaprevir.

[r] Adverse events reported by subjects after signing of the informed consent and before the first dose of telaprevir and those reported more than 30 days after administration of the last dose of telaprevir should be monitored according to clinical practice and noted in the source document.

[s] Only protocol-defined adverse events (see Safety Evaluations in Synopsis), whether serious or non-serious, that occur between the first use of investigational product (telaprevir) and 30 days after administration of the last dose of telaprevir will be reported in the CRF. The DAIDS criteria will be used to grade adverse events except for rash which has protocol-specific definitions of severity grades.

[t] Special reporting situations (eg, overdose, suspected abuse/misuse, inadvertent or accidental exposure, or medication error of telaprevir) and Product Quality Complaints (PQCs) should be reported in addition to adverse events.

β-HCG=β-human chorionic gonadotropin; CRF=case report form; DAIDS=Division of AIDS Table for Grading the Severity of Adult and Pediatric Adverse Events; HbsAg=hepatitis B surface antigen; HCV=hepatitis C virus; HIV=human immunodeficiency virus; Peg-IFN-alfa=pegylated interferon alfa; RBV=ribavirin; RNA= ribonucleic acid.

ABBREVIATIONS

AGEP	acute generalized exanthematous pustulosis
ALT	alanine aminotransferase
AST	aspartate aminotransferase
β-hCG	β-human chorionic gonadotropin
CRF	case report form
CI	confidence interval
CYP	cytochrome P450
DAA	direct acting antiviral
DAIDS	Division of AIDS Table for Grading the Severity of Adult and Pediatric Adverse Events
DRESS	drug-related eosinophilia with systemic symptoms
DS	delayed start
eDC	electronic data capture
ECG	electrocardiogram
EM	erythema multiforme
GCP	Good Clinical Practice
GGT	gamma-glutamyltransferase
HCC	hepatocellular carcinoma
HCV	hepatitis C virus
HIV	human immunodeficiency virus
ICH	International Conference on Harmonisation
IEC	Independent Ethics Committee
IRB	Institutional Review Board
Peg-IFN	pegylated interferon
PQC	Product Quality Complaint
q8h	every 8 hours
RBV	ribavirin
RNA	ribonucleic acid
SJS	Stevens-Johnson syndrome
SVR	sustained virologic response
SVR_{24}	sustained virologic response 24 weeks after last dose of study drug
TEN	toxic epidermal necrolysis
WBC	white blood cell
WT	wild type

DEFINITION OF TERMS

End of treatment with study drug	Week 12 (telaprevir)
End of treatment with all drug	Treatment naïve or prior treatment relapsers with severe fibrosis (Metavir F3 or Ishak 3-4): Week 24 or 48, depending on virologic response or early discontinuation.<br>Previously treated subjects with prior partial or prior null response, or who had viral breakthrough, with severe fibrosis (Metavir F3 or Ishak 3-4) and all subjects with cirrhosis (Metavir F4 or Ishak 5-6): Week 48 or early discontinuation.
Standard-of-care treatment	Treatment with Peg-IFN-alfa and RBV for 24 or 48 weeks
Stopping rules	• Telaprevir should be discontinued if HCV RNA is >100 IU/mL at Week 4.<br>• Peg-IFN-alfa/RBV should be discontinued if HCV RNA is >100 IU/mL at Week 12.<br>• Beyond Week 12, refer to the local prescribing information for Peg-IFN-alfa and RBV.

1. INTRODUCTION

Telaprevir (VX-950, TVR) is being developed by Vertex Pharmaceuticals Incorporated (Vertex) in collaboration with the Johnson & Johnson companies Janssen Pharmaceutica NV and Tibotec BVBA. Telaprevir is a member of a new class of drugs being developed for chronic hepatitis C: Direct Acting Antiviral (DAA) agents. Unlike pegylated interferon (Peg-IFN) and ribavirin (RBV), DAA agents act directly on the hepatitis C virus (HCV) replication cycle. Telaprevir is a specific, reversible, covalent, tight- and slow-binding NS3•4A inhibitor that was derived through structure-based drug design. Telaprevir prevents HCV replication by inhibiting the HCV NS3•4A protease, which is an enzyme that is essential for HCV replication.

Telaprevir is currently in Phase 3 of clinical development and submission for marketing authorization in the European Union was completed in December 2010. Submissions in other countries are planned.

For the most comprehensive nonclinical and clinical information regarding the efficacy and safety of telaprevir, refer to the latest version of the Investigator's Brochure for Telaprevir (VX-950, TVR).[1]

The term sponsor used throughout this document refers to the entities listed in the Contact Information page(s), which will be provided as a separate document.

1.1. Background

Epidemiology of Chronic Hepatitis C

Since the discovery of HCV and development of a serologic assay for HCV antibody in 1989,[2] hepatitis C has been identified as a major cause of chronic liver disease throughout the world. The World Health Organization (WHO) estimates that 170 million people,[3] approximately 3% of the global population, are infected with HCV. In developed countries, HCV infection is the most common reason for liver transplantation.

Liver Disease and Primary Hepatocellular Carcinoma due to HCV

After human immunodeficiency virus (HIV), HCV has the second highest rate of morbidity and mortality amongst recently discovered infectious diseases.[4] Hepatitis C virus is the most common infectious cause of chronic liver disease in Europe.[5]

Chronic HCV infection causes hepatic inflammation, which typically progresses to varying degrees of hepatic fibrosis, including cirrhosis. Once cirrhosis is present, the patient is at risk for hepatic decompensation (ascites, variceal bleeding, portosystemic encephalopathy), development of primary hepatocellular carcinoma (HCC), need for liver

transplant, and death due to liver disease.[6] It is estimated that 20% to 30% of patients with chronic hepatitis C progress to cirrhosis.[7]

From prospective studies, more accurate data are available on the risk of hepatic decompensation, HCC, and survival rates once advanced hepatic fibrosis and cirrhosis are diagnosed.[8,9,10] In subjects with clinically compensated cirrhosis, the cumulative probability of an episode of hepatic decompensation is 5% at 1 year, and increases to 30% at 10 years from the diagnosis of cirrhosis. Estimated survival rates at 3, 5, and 10 years are 96%, 91%, and 79%. Once hepatic decompensation occurs, however, the 5-year survival rate falls to 50% without liver transplantation.

The recently completed HALT-C study provides more detailed information on the rate of clinical outcomes (death, HCC, ascites, hepatic encephalopathy, variceal hemorrhage, spontaneous peritonitis, and persistent elevation of the Child-Turcotte-Pugh score ≥7) in a cohort of 1,050 subjects in the United States with advanced hepatic fibrosis (Ishak 3 to 6) who were prospectively followed over 3.5 years.[11,12] Among subjects with cirrhosis at baseline, 28.7% developed a clinical outcome, and among those with bridging fibrosis, 21.5% advanced to cirrhosis over 3.5 years.

Hepatitis C virus infection is one of the most important factors to HCC development; the risk of HCC is 17 times greater in HCV-infected patients compared to HCV-negative individuals. Markers of HCV infection are found in a high proportion of HCC patients in a number of European countries such as France (27% to 58%).

Treatment for HCV: Defining Unmet Medical Needs

<u>Sustained Virologic Response</u>

Sustained virologic response (SVR; defined as the absence of detectable HCV RNA 24 weeks after completion of antiviral treatment) has been universally accepted as a surrogate endpoint of permanent clearance of this chronic viral infection. As recently reviewed by Nelson, based on numerous long-term follow-up studies conducted over the 23 years since patients were first treated with alfa interferon for hepatitis C, several important benefits can be attributed to achieving SVR, such as regression of hepatic fibrosis,[13,14,15] reduced risk of long-term complications of liver disease, reductions in impaired quality of life, and rates of hepatic decompensation.[16]

<u>Treatment of HCV Genotype 1</u>

The current treatment regimen for chronic HCV genotype 1 is a combination of Peg-IFN-alfa and RBV, and these regimens are approved for 2 categories of patients: those who have never received treatment for hepatitis C (treatment-naïve) and those who

have received previous treatment with alfa interferon regimens and did not develop SVR (treatment-failure).

Overall SVR rate in genotype 1 patients is around 40% to 50%.[17,18] The results of re-treatment trials show that SVR rates for non-responders were <10% for Peg-IFN and RBV regimens of 48 weeks.

Advanced Liver Disease, Cirrhosis

Across all interferon studies for subjects with HCV, the presence of advanced hepatic fibrosis (Metavir F3, F4; Ishak stage 3 to 6) is associated with a lower likelihood of SVR. These subjects are also at greatest risk of liver failure, HCC, and death.[19,20]

In the HALT-C study, subjects with an Ishak stage 3 or greater hepatic fibrosis, and who had not achieved SVR with prior interferon treatment with or without RBV, were re-treated with Peg-IFN and RBV.[11,12] In the first 604 subjects, 539 (89%) of whom were infected with genotype 1, only 18% of subjects achieved SVR.[21] An additional analysis conducted in the entire cohort of 1,046 subjects showed that reduction in SVR rate was a function of increasingly severe liver disease: the SVR rate was 23% in subjects with bridging fibrosis and platelet counts >125,000 cells/mm^3 and 9% in subjects with cirrhosis and platelet counts ≤125,000 cells/mm^3.[22] By logistic regression, cirrhosis was the major determinant that impaired virologic response, independent of dose reduction or platelet count.

Efficacy Results of Telaprevir Addressing This Urgent Unmet Medical Need

A Phase 2 program that includes 5 studies, all completed, has demonstrated the benefit of adding telaprevir to the Peg-IFN and RBV standard regimen for the treatment of subjects chronically infected with HCV genotype 1.[1] These results were confirmed in a large Phase 3 trial.

Study VX-950-108 (ADVANCE) was a randomized, double-blind, placebo-controlled, parallel-group, multicenter Phase 3 study conducted in 1,088 treatment-naïve subjects with genotype 1, chronic HCV infection. The study compared 2 regimens of telaprevir dosed with Peg-IFN-alfa-2a and RBV against standard treatment, Peg-IFN-alfa-2a and RBV. Telaprevir was given for the first 8 weeks or the first 12 weeks in combination with Peg-IFN-alfa-2a and RBV for either 24 or 48 weeks in duration. For subjects receiving a telaprevir-based regimen with undetectable HCV RNA at Week 4 and 12, Peg-IFN-alfa-2a and RBV were dosed for a total of 24 weeks. For subjects who did not achieve undetectable HCV RNA at Week 4 and 12, Peg-IFN-alfa-2a and RBV were dosed for a total of 48 weeks. The control group had a fixed treatment duration

of 48 weeks, with telaprevir-matching placebo given for the first 12 weeks and Peg-IFN-alfa-2a and RBV dosed for 48 weeks.

Sustained virologic response rates were significantly higher in the 8- and 12-week telaprevir-based combination groups (68.7% and 74.7%, respectively), compared with the Peg-IFN-alfa-2a/RBV 48-week control group (43.8%). Relapse rates were lower in the 8- and 12-week telaprevir-based combination groups (9.5% and 8.6%, respectively) than in the control group (27.9%). For subjects with bridging fibrosis and cirrhosis, SVRs in the 8- and 12-week telaprevir-based combination groups were 53% (45/85) and 62% (45/73), respectively, and 33% (24/73) in the control group. For subjects without bridging fibrosis and cirrhosis, SVRs in the 8- and 12-week telaprevir-based combination groups were 73% (205/279) and 78% (226/290), respectively, and 47% (135/288) in the control group.[23,24]

Two Phase 2 studies evaluated HCV genotype 1 in chronically infected subjects who had previously failed Peg-IFN and RBV based therapy (Studies 106 and 107) and one Phase 3 trial, REALIZE.[1]

Study 106 was a randomized, stratified, partially placebo-controlled, and partially double-blinded study to assess the efficacy and safety of 12 to 24 weeks of telaprevir in combination with various durations of Peg-IFN-alfa-2a treatment, with or without RBV. This study evaluated well-characterized subjects who had previously received an adequate course of Peg-IFN (alfa-2a or alfa-2b) and RBV but did not achieve SVR. Randomization was stratified based on whether subjects had or did not have undetectable HCV RNA during the prior course of Peg-IFN/RBV treatment. Of the 453 subjects enrolled in Study 106, 260 (57%) were non-responders to their prior treatment and 162 (36%) had relapsed after prior treatment; 16% of subjects had cirrhosis. Unprecedented and statistically significantly higher overall SVR rates were observed in the telaprevir, Peg-IFN, and RBV treatment groups (51% to 52%) compared to that in the control arm (14%) ($p<0.001$) for this very difficult-to-treat population. In the overall population, SVR rates in the telaprevir, Peg-IFN, and RBV arms were similar across subjects without cirrhosis (51% to 54.8 %) compared to those with cirrhosis at baseline (53% and 45%).[1]

Study 107 is an open-label study for subjects who failed therapy in the control arms of Studies 104, 104EU, and 106. This study allows for a very clear assessment of the response to Peg-IFN-alfa-2a and RBV treatment in the previous study. On-treatment antiviral activity in response to the combination of telaprevir, Peg-IFN-alfa-2a, and RBV was demonstrated in all profiles of previous treatment-failure subjects (null responders,

partial responders, and relapsers). Final SVR_{24} (SVR 24 weeks after last dose of study drug) results demonstrated for the first time that treatment with telaprevir and Peg-IFN-alfa-2a and RBV can result in SVR in previous null responders to Peg-IFN and RBV (16 of 28 subjects treated with telaprevir 12 week/Peg-IFN-alfa-2a and RBV 48 week).[1]

Study VX-950-TiDP24-C216 (REALIZE) was a Phase 3 study designed to compare the efficacy, safety, and tolerability of 2 regimens of telaprevir administered for 12 weeks (with and without delayed start [DS] of telaprevir) combined with 48 weeks of Peg-IFN-alfa-2a and RBV versus standard treatment (Peg-IFN-alfa-2a and RBV for 48 weeks). Telaprevir was administered at a dose of 750 mg every 8 hours (q8h) and Peg-IFN-alfa-2a and RBV at standard doses, ie, 180 μg once weekly and 1,000 or 1,200 mg/day (weight-based), respectively. In total, 663 subjects were randomized in a 2:2:1 ratio to 1 of 3 treatment groups, of whom 662 subjects were treated: 266 subjects in the 12-week telaprevir-based combination group, 264 subjects in the 12-week DS telaprevir-based combination group, and 132 subjects in the Peg-IFN-alfa-2a/RBV 48-week control group.

This study included well-characterized prior relapsers and prior non-responders (prior null-responders and prior partial responders). Of the 662 subjects who were treated, 354 (53.5%) subjects were prior relapsers and 308 (46.5%) subjects were prior non-responders. Among the prior non-responder population, 184 (59.7%) subjects were prior null responders and 124 (40.3%) subjects were partial responders.

The proportion of subjects achieving SVR_{24} was statistically significantly higher in each of the telaprevir treatment groups (with and without delayed start) than in the control group for prior relapsers and prior non-responders separately. For the prior relapser population, SVR_{24} planned rates were 83.4% and 87.9% for the 12-week and 12-week DS telaprevir-based treatment groups, respectively, compared with 23.5% for the control group (both p values compared with control <0.001). For the prior non-responder population, SVR rates were 41.3% and 41.5% for the 12-week and 12-week DS telaprevir-based treatment groups, respectively, compared with 9.4% for the control group (both p values compared with control <0.001). SVR_{24} planned rates were also statistically significantly higher in each of the telaprevir-based treatment groups than in the control group in the 2 subgroups of prior non-responders: prior null-responders and prior partial responders. For the prior null-responder population, SVR rates were 29.2% and 33.3% for the 12-week and 12-week DS telaprevir-based treatment groups, respectively, compared with 5.4% for the control group. For the prior partial responder population, SVR rates were 59.2% and 54.2% for the 12-week and 12-week

DS telaprevir-based treatment groups, respectively, compared with 14.8% for the control group.[25]

1.2. Overall Rationale for the Study

Clinical studies in treatment-naïve and treatment-failure subjects have demonstrated a statistically significant benefit (higher rate of SVR) by adding telaprevir to the Peg-IFN-alfa-2a and RBV standard regimen for the treatment of subjects chronically infected with HCV genotype 1. Patients with advanced hepatic fibrosis or cirrhosis (Metavir F3, F4; Ishak stage 3 to 6) have a lower likelihood of SVR and also have the greatest risk of liver failure, HCC, and death. Making telaprevir available to this difficult-to-treat population may reduce the risk of long-term complications associated with HCV infection.

2. OBJECTIVES

The objectives of this early access program are to provide telaprevir for subjects with genotype 1 chronic hepatitis C with severe fibrosis and compensated cirrhosis who reside in countries in which telaprevir is not yet commercially available and who are not eligible for enrollment into an ongoing clinical study of telaprevir, and to collect additional safety and tolerability data on telaprevir treatment in combination with Peg-IFN-alfa and RBV.

Hypothesis:

In this early access program, all subjects will receive open-label telaprevir in addition to standard-of-care treatments. Only selected safety and tolerability data will be collected. As such, no formal statistical hypothesis will be tested.

3. OVERVIEW OF STUDY DESIGN

Approximately 3,000 subjects in countries in which telaprevir is not yet commercially available and who are not eligible for enrollment into an ongoing clinical study of telaprevir are expected to be enrolled in this early access program. All subjects must sign informed consent prior to the conduct of any procedures for the early access program.

The number of subjects is derived from practical considerations related to the projected number of subjects eligible to benefit from early access to telaprevir in the included countries and not on statistical considerations. However, some guidance is provided on the expected precision of estimation of safety parameters (see Section 11.4, Sample Size Determination).

3.1. Study Design

This is a multicenter, multinational, open-label, non-comparative early access program designed to provide telaprevir to subjects who are expected to benefit from

telaprevir-based therapy and to collect additional safety and tolerability data on telaprevir treatment in combination with Peg-IFN-alfa and RBV. Enrollment of subjects into this early access program will continue until telaprevir becomes available for reimbursement in the country in which a subject resides, or until September 2013, whichever occurs first, unless other guidelines applicable per local regulations. Subjects already enrolled in the early access program when enrollment is stopped should complete treatment (ie, telaprevir, Peg-IFN-alfa/RBV) and continue follow-up according to the protocol schedule.

All subjects eligible to enter the study will receive telaprevir 750 mg q8h during the first 12 weeks of the early access program in combination with standard-of-care (Peg-IFN-alfa/RBV). The duration of Peg-IFN-alfa/RBV will be as follows:

- Subjects with severe fibrosis (Metavir F3 or Ishak 3-4) who are treatment naïve or prior treatment relapsers will subsequently be treated with Peg-IFN-alfa/RBV for either an additional 12 or 36 weeks (total treatment duration of 24 or 48 weeks, respectively) based on virologic response to treatment as measured by the Week 4 and 12 plasma HCV RNA levels reported by the local laboratory.
  - Subjects with undetectable HCV RNA at Weeks 4 and 12 will receive an additional 12 weeks of Peg-IFN-alfa/RBV alone for a total treatment duration of 24 weeks.
  - Subjects with detectable HCV RNA at either Week 4 or 12 will receive an additional 36 weeks of Peg-IFN-alfa/RBV alone for a total treatment duration of 48 weeks; see stopping rules for further guidelines.
- Previously treated subjects with prior partial or prior null response, or who had viral breakthrough, with severe fibrosis (Metavir F3 or Ishak 3-4) and all subjects with cirrhosis (Metavir F4 or Ishak 5-6) will subsequently be treated with Peg-IFN-alfa/RBV alone for an additional 36 weeks (total treatment duration of 48 weeks); see stopping rules for further guidelines.

For all subjects, stopping rules based on virologic response (ie, HCV RNA levels) will be applied to ensure that telaprevir or Peg-IFN-alfa/RBV treatments are stopped if subjects have viral breakthrough or failure. At Weeks 4 and 12, the stopping rules presented in the table below will be applied. For guidelines for treatment discontinuation after Week 12 of treatment, refer to the local prescribing information for Peg-IFN-alfa and RBV.

Guidelines for Discontinuation of Telaprevir, Peg-IFN-alfa and RBV Treatment

Medicinal product(s)	> 100 IU/mL at Week 4 of treatment	> 100 IU/mL at Week 12 of treatment
Telaprevir	Permanently discontinue	Telaprevir treatment completed
Peg-IFN-alfa/RBV	Continue to Week 12 decision point	Permanently discontinue

Additional plasma HCV RNA levels should be measured at Week 24 for subjects with a total treatment duration of 24 weeks and at Weeks 24 and 48 for subjects with a total

treatment duration of 48 weeks. Additionally, all subjects should have a follow-up visit, including measurement of plasma HCV RNA levels, 24 weeks after the last administered dose of any treatment (ie, telaprevir, Peg-IFN-alfa, or RBV) (eg, Week 48 for subjects receiving 24 weeks of treatment, Week 72 for subjects requiring 48 weeks of treatment).

Subjects who discontinue all treatments (ie, telaprevir, Peg-IFN-alfa, and RBV) early or withdraw early from the early access program should have an early treatment discontinuation/early withdrawal visit performed and a follow-up visit performed 24 weeks after the last administered dose of treatment, unless informed consent for further follow-up has been withdrawn. Plasma HCV RNA levels should be measured at each of these visits.

Recommended evaluations of safety and tolerability to be performed during the early access program are presented in the Time and Events Schedule that follows the Synopsis.

A diagram of the early access program is provided below.

Schematic Overview of the Early Access Program

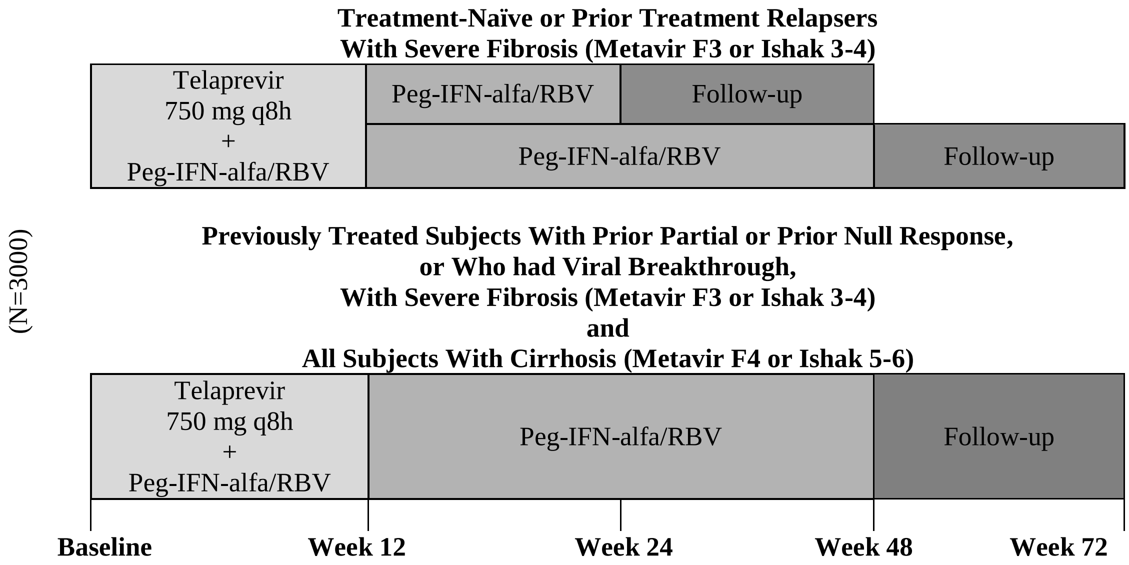

Peg-IFN-alfa/RBV=pegylated interferon alfa and ribavirin; q8h=every 8 hours.

3.2. Study Design Rationale

3.2.1. Study Population

The population for the early access program includes subjects with HCV compensated liver disease and severe liver fibrosis or cirrhosis (Metavir F3, F4; Ishak stage 3 to 6), who are treatment naïve, prior treatment relapsers, or were previously treated with prior

partial or prior null response, or who had viral breakthrough, and who have limited treatment options. See Section 1.2, Overall Rationale for the Study, for additional details.

3.2.2. Telaprevir Dose

A telaprevir dose of 750 mg q8h was selected based on the data generated in Trial VX04-950-101, a Phase 1b, multiple-dose, dose-escalation trial with telaprevir in healthy subjects and subjects with HCV infection. Interim efficacy data from 2 Phase 2 trials in treatment-naïve subjects (Trials 104 and 104EU) and a Phase 2 trial in treatment-failure subjects (Trial 106) show that 12 weeks of telaprevir, at a dose of 750 mg q8h, in combination with different durations of Peg-IFN-alfa-2a and RBV is associated with higher SVR rates than those that can be expected with standard treatment.

Lowering the telaprevir dose below 750 mg q8h is expected to lead to an increased rate of breakthrough with viral variants with low levels of telaprevir resistance. The results of viral dynamic modeling analyses predicted that reducing telaprevir dose by 50% (375 mg q8h) would result in a 2-fold increase in the rate of breakthrough by viral variants with low levels of telaprevir resistance.

3.2.3. Telaprevir Duration

Simulated trajectories of HCV RNA levels attributed to the wild-type (WT) virus and defined variants have been generated for representative subjects having varying responses to treatment with Peg-IFN-alfa-2a and RBV, and have been compared for dosing regimens that incorporated either 4 weeks or 12 weeks of telaprevir co-administered with 48 weeks of treatment with Peg-IFN-alfa-2a and RBV.[26] For a null-responder to Peg-IFN-alfa-2a/RBV, neither 4 weeks nor 12 weeks of telaprevir dosing was predicted to eliminate HCV. For a partial or slow responder to Peg-IFN-alfa-2a/RBV, the outcomes predicted for 4 weeks versus 12 weeks of telaprevir dosing were different. In contrast to the 4-week telaprevir regimen, 12 weeks of telaprevir dosing in a subject with a slow Peg-IFN-alfa-2a/RBV response was predicted to eliminate WT virus, viral variants with low levels of telaprevir resistance, and variants with high levels of telaprevir resistance by approximately 10 weeks, thereby preventing relapse of HCV after termination of telaprevir dosing. For an average Peg-IFN-alfa-2a/RBV responder, both 4 weeks and 12 weeks of telaprevir dosing were predicted to eliminate all HCV. Lower breakthrough rates with WT virus and viral variants with low levels of telaprevir resistance during the Peg-IFN-alfa-2a/RBV dosing period following telaprevir dosing were predicted for longer duration of telaprevir dosing (12 weeks as compared to 4 weeks). These predictions were confirmed by the results of Trials 104 and 104EU (in treatment-naïve subjects). On the basis of these results, a telaprevir duration of 12 weeks was selected.

In Trial 106 (in treatment-failure subjects), the effect of prolonging the telaprevir duration to 24 weeks was investigated. It was observed that telaprevir dosing for 24 weeks was associated with more treatment discontinuations and dose adjustments due to poor tolerance compared to 12 weeks of telaprevir duration. Furthermore, no evidence of additional virologic benefit was observed with the longer telaprevir dosing. These findings support the proposed 12-week telaprevir treatment duration.

3.2.4. Peg-IFN-Alfa and RBV Duration

The efficacy results from Trials 104 and 104EU in treatment-naïve subjects show that a regimen with a total duration of 24 weeks (12 weeks of telaprevir in combination with 24 weeks of Peg-IFN-alfa-2a and RBV) had a low relapse rate, indicating that this treatment duration was sufficient to keep relapse rate at a minimum. The findings of Trial 106 indicate that this treatment duration may not be sufficient for a treatment failure population. In this trial, higher relapse rates, compared to those obtained in treatment-naïve subjects, were observed in the telaprevir-treated subjects that completed a total of 24 weeks of therapy: 29% overall, 40% for non-responders, and 18% for relapsers.

Therefore, a longer treatment duration (ie, 12 weeks of telaprevir in combination with 48 weeks of Peg-IFN-alfa-2a and RBV) was assessed in a Phase 3 trial of subjects that failed a prior Peg-IFN/RBV treatment. The treatment duration of Peg-IFN-alfa and RBV in combination with telaprevir in this early access program for previously treated subjects with prior partial or prior null response, or who had viral breakthrough, will be 48 weeks based on the results of the Phase 3 study (REALIZE) presented in Section 1.1, Background, and will be different than what is in the current Peg-IFN-alfa-2a label with a recommendation of up to 72 weeks treatment duration in a subgroup of patients not responding to prior treatment with Peg-IFN and RBV.[27]

In previously treated subjects, when available, the use of Peg-IFN-alfa-2a in combination with telaprevir and RBV should be considered due to the limited data with Peg-IFN-alfa-2b.

3.2.5. Treatment Modifications Based on Virologic Response

Evolution of viral population evidenced by an additional gain in mutations is expected if telaprevir dosing is continued in subjects with viral breakthrough (defined as an increase of >1 log HCV RNA compared to the lowest recorded on-treatment value, or if the HCV RNA had become undetectable, a confirmed increase to HCV RNA >100 IU/mL). This was seen in some subjects in Trial 104EU. However, evolution of resistant variants may be limited if telaprevir dosing is stopped after breakthrough is detected, as observed

for some subjects in Trial 104. The majority of breakthroughs (75%) occurred within 4 weeks after the start of telaprevir treatment. These results highlight the importance of closely monitoring subjects and discontinuing telaprevir dosing in case of viral breakthrough or virologic failure.

To ensure that viral breakthrough or failure has not occurred, subjects must have plasma HCV RNA levels monitored throughout the study. Telaprevir should be discontinued if HCV RNA is >100 IU/mL at Week 4. Peg-IFN-alfa/RBV should be discontinued if HCV RNA is >100 IU/mL at Week 12. Beyond Week 12, the guidelines for treatment discontinuation in the local prescribing information for Peg-IFN-alfa and RBV will be followed. The value of 100 IU/mL was selected as it offers good discrimination between subjects who are virologic failures as opposed to those with a slower response to the treatment regimen.

4. SUBJECT SELECTION

The inclusion and exclusion criteria for enrolling subjects in this early access program are described in the following 2 subsections. If there is a question about the inclusion or exclusion criteria below, the investigator should consult with the appropriate sponsor representative before enrolling a subject in the early access program.

4.1. Inclusion Criteria

Each potential subject must satisfy all of the following criteria to be enrolled in the early access program. Each subject must:

1. Be a man or woman, between 18 and 70 years of age, inclusive
2. Have evidence of HCV infection genotype 1 (molecular assay)
3. Have a quantifiable plasma HCV RNA
4. Have documentation of liver fibrosis assessed by liver biopsy or non-invasive test (eg, fibrotest, fibroscan) showing severe fibrosis (Metavir F3 or Ishak 3-4) or cirrhosis (Metavir F4 or Ishak 5-6). For subjects with Metavir F3 or Ishak 3-4, the liver biopsy or non-invasive test should have been performed within the past 18 months.
5. Have compensated liver disease (Child-Pugh Grade A clinical classification) (see Attachment 1)
6. Have access to the Hepatitis C standard-of-care (Peg-IFN-alfa/RBV)

7. If a women of childbearing potential, must have a negative serum β-human chorionic gonadotropin (β-hCG) pregnancy test documented at the screening visit and a negative serum or urine pregnancy test before the first dose of study drug to ensure that they are not pregnant at the time of starting treatment.

8. If heterosexually active, a female subject of childbearing potential and a nonvasectomized male subject who has a female partner of childbearing potential must agree to use 2 effective contraceptives from screening onwards until 4 months (female subject) or 7 months (male subject) after all therapy has ended.

   Note: Hormonal contraceptives may not be reliable during telaprevir dosing. Therefore, to be eligible for this early access program, subjects should use 2 other effective birth control methods during telaprevir combination therapy and for 2 months after the last intake of telaprevir (see also Section 4.3).

9. Sign the informed consent document indicating that they understand the purpose of and procedures required for the early access program and are willing to participate in the early access program.

4.2. Exclusion Criteria

Any potential subject who meets any of the following criteria will be excluded from participating in the early access program. The subject will be excluded if he or she:

1. Is eligible for enrollment into an ongoing clinical study of telaprevir

2. Is infected or co-infected with HCV of another genotype than genotype 1

3. Has a contraindication to the administration of Peg-IFN-alfa or RBV, or medical history or laboratory values that preclude treatment with Peg-IFN-alfa or RBV according to the respective local prescribing information

4. Has a history of having received investigational HCV protease or polymerase inhibitors at any previous time

5. Has signs or symptoms of HCC. Serum alpha-fetoprotein (AFP) level and ultrasonography should be available at screening for all subjects to screen for HCC (both tests should have been done a maximum of 4 months before the screening visit).

6. Has a history of decompensated liver disease: history of ascites, hepatic encephalopathy, or bleeding esophageal varices, and/or any of the following screening laboratory results:
   - International Normalized Ratio (INR) of ≥1.5
   - Serum albumin <3.3 g/dL
   - Serum total bilirubin >1.8 times the upper limit of the laboratory normal range, unless isolated or in subjects with Gilbert's Syndrome.

7. Has a co-infection with active hepatitis B or HIV

8. Has any of the following laboratory abnormalities (assessed at local laboratory) as defined by the Division of AIDS Table for Grading the Severity of Adult and Pediatric Adverse Events (DAIDS).
   - Absolute neutrophil count (ANC) <1,500 cells/mm^3
   - Platelet count <90,000 cells/mm^3
   - Hemoglobin concentration <12 g/dL in females or <13 g/dL in males
   - Calculated creatinine clearance <50 mL/min
   - potassium <3.5 mmol/L

9. Has inadequately controlled thyroid function (TSH)

10. Has baseline increased risk for anemia (eg, thalassemia, sickle cell anemia, spherocytosis, history of gastrointestinal bleeding) or for whom anemia would be medically problematic

11. Has congenital QT prolongation or family history of congenital QT prolongation or sudden death

12. Has a history of severe psychiatric disease, including psychosis and/or depression, characterized by a suicide attempt, hospitalization for psychiatric disease, or a period of disability as a result of psychiatric disease

13. Has a history of immunologically mediated disease (eg, inflammatory bowel disease, idiopathic thrombocytopenic purpura, lupus erythematosus, autoimmune hemolytic anemia, scleroderma, severe psoriasis [defined as affecting >10% of the body, where the palm of one hand equals 1%, or if the hands and feet are affected], rheumatoid arthritis requiring more than intermittent nonsteroidal anti-inflammatory medications for management)

14. Has clinical evidence of chronic pulmonary disease associated with functional impairment

15. Has a history of uncontrolled severe seizure disorders

16. Has a history or other evidence of a clinically relevant ophthalmologic disorder due to diabetes mellitus or hypertension or history or other evidence of severe retinopathy (eg, cytomegalovirus, macular degeneration)

17. Has a history of major organ transplantation with an existing functional graft with the exception of corneal transplants and skin grafts

18. Is currently enrolled in an investigational drug study or has participated in such a study within 30 days before Day 1

19. Is a woman who is pregnant or breast-feeding

20. Has any condition that, in the opinion of the investigator, would compromise the well-being of the subject or the early access program or prevent the subject from meeting or performing requirements of the early access program

4.3. Prohibitions and Restrictions

Refer to Section 8, Prestudy and Concomitant Therapy for contraindications to telaprevir. Because telaprevir must be used in combination with RBV and Peg-IFN, the contraindications and warnings applicable to those medicinal products are applicable to combination therapy. For contraindications and warnings to Peg-IFN-alfa and RBV, please refer to the relevant local prescribing information of these products.

The prohibitions and restrictions required for participation in this early access program were based on the following:

- Telaprevir has shown no teratogenic potential in rats and mice and is not considered a developmental toxicant in these species. Significant teratogenic and/or embryocidal effects have been demonstrated in all animal species exposed to RBV. Extreme care must be taken to avoid pregnancy in female subjects (see Prohibitions and Restrictions Criteria 1, 2, and 3) and in female partners of male subjects (see Prohibitions and Restrictions Criteria 1 and 4; also refer to the local prescribing information for RBV).
- In female subjects, the co-administration of telaprevir with an estrogen-based hormonal contraceptive reduced ethinylestradiol exposure (see Prohibitions and Restrictions Criterion 5).
- It is not known whether telaprevir is excreted in human breast milk. When administered to lactating rats, levels of telaprevir and its major metabolite were higher in milk compared to those observed in plasma. Rat offspring exposed to telaprevir in utero showed normal body weight at birth. However, when fed via milk from telaprevir-treated dams, body weight gain of rat pups was lower than normal (likely due to taste aversion). After weaning, rat pup body weight gain returned to normal. (See Prohibitions and Restrictions Criterion 6; local prescribing information for RBV.)
- Telaprevir had no effects on fertility or fecundity when evaluated in rats.

Potential subjects must be willing and able to adhere to the prohibitions and restrictions described in this section during the course of the early access program to be eligible for participation.

1. As any birth control method can fail, at least 2 reliable forms of effective contraception must be used.

2. Female subjects of childbearing potential and their male partners must use 2 effective contraceptive methods during treatment and for 4 months after all treatment has ended

3. For female subjects of childbearing potential, pregnancy testing (serum or urine) should occur monthly during telaprevir combination therapy and for 4 months after all treatment has ended

4. Male subjects and their female partners of childbearing potential must use 2 effective contraceptive methods during treatment and for 7 months after all treatment has ended. Male subjects whose partners are pregnant must be instructed to use a condom to minimize exposure of RBV to the partner. Pregnancy testing in non-pregnant female partners is recommended before telaprevir combination therapy, every month during telaprevir combination therapy, and for 7 months after RBV therapy has ended.

5. Estrogen-based hormonal contraceptives may not be reliable during telaprevir dosing. Therefore, female subjects of childbearing potential should use 2 additional methods of effective birth control during telaprevir dosing and for 2 months after the last intake of telaprevir. Examples of non-hormonal methods of contraception include a male condom with spermicidal jelly OR female condom with spermicidal jelly (a combination of a male condom and a female condom is not suitable), a diaphragm with spermicidal jelly, or a cervical cap with spermicidal jelly. As of 2 months after completion of telaprevir treatment, estrogen-based hormonal contraceptives can again be used as 1 of the 2 required effective methods of birth control; however, specific local prescribing information recommendations should be respected.

6. Because of the potential for adverse reactions in breast-fed infants, breast-feeding must be discontinued prior to initiation of therapy.

5. TREATMENT ALLOCATION

This is an open-label early access program, in which all subjects will receive telaprevir 750 mg orally q8h for 12 weeks in combination with standard-of-care consisting of Peg-IFN-alfa and RBV followed by Peg-IFN-alfa and RBV given for additional 12 or 36 weeks based on virologic response to treatment and/or type of subject (treatment naïve or prior treatment relapsers, or previously treated with prior partial or prior null response, or who had viral breakthrough; and Metavir/Ishak score), as described in Section 3.1.

6. DOSAGE AND ADMINISTRATION

Telaprevir must be used in combination with Peg-IFN-alfa and RBV.

Investigators participating in the early access program will assume responsibility for the subjects' compliance with all procedures and guidelines for the use of telaprevir as outlined in the protocol, the Investigator's Brochure, and other early access program-related materials provided by the sponsor or their designee.

Telaprevir (750 mg given as two 375 mg film-coated tablets) will be administered orally q8h with food (the total daily dose is 6 tablets [2,250 mg]). Subjects should be instructed to swallow the tablets whole (eg, subjects should not chew, break, or dissolve the tablet).

In case a dose of telaprevir is missed within 4 hours of the time it is usually taken, subjects should be instructed to take the prescribed dose of telaprevir with food as soon as possible. If the missed dose is noticed more than 4 hours after the time telaprevir should be taken, the missed dose should be skipped and the subject should resume the normal dosing schedule.

Dose modifications of telaprevir are prohibited and once telaprevir treatment is discontinued for safety reasons or inadequate virologic response, it may not be reinitiated.

In addition to the administration of telaprevir, subjects enrolled in this early access program will be administered the prescribed standard-of-care products Peg-IFN-alfa and RBV. Concomitant administration of Peg-IFN-alfa and RBV will follow label recommendations of each product and the duration of therapy with these drugs will vary by early virologic response and/or by type of subject (treatment naïve or prior treatment relapsers, or previously treated with prior partial or prior null response, or who had viral breakthrough; and Metavir/Ishak score); see Section 3.1, Study Design. In previously treated subjects, when available, the use of Peg-IFN-alfa-2a in combination with telaprevir and RBV should be considered due to the limited data with Peg-IFN-alfa-2b. It is the responsibility of the investigator to ensure that the guidelines for administration, missed doses, contraindications, and special warnings, as outlined in the label recommendations of these products, are adhered to according to the requirements of Good Clinical Practice (GCP).

To maximize adherence to dosing regimens and minimize inconvenience for subjects, RBV, which is taken twice daily, may be taken at the same time as the morning and evening telaprevir doses (rather than every 12 hours [q12h]). If the number of daily RBV tablets is an odd number, the larger number of tablets should be taken with the morning dose (ie, 3 tablets in the morning and 2 at night, so the larger dose is given before the longer period between doses). If RBV-related insomnia is a concern, then the second dose of RBV may be administered with the afternoon telaprevir dose instead of the evening dose.

The preset stopping rules based on virologic response for telaprevir, Peg-IFN-alfa, and RBV are presented in Section 3.1, Study Design, and additional guidelines are presented in Section 10.2, Discontinuation of Treatment. For temporary interruptions of Peg-IFN-alfa or RBV in case of rash, see Section 9.3.1.1.3, Rash Management.

The investigator's staff will instruct subjects on how to store medication for at-home use as indicated for this protocol.

7. TREATMENT COMPLIANCE

The investigator or designated personnel will maintain a log of telaprevir dispensed and returned. Study drug supplies (telaprevir) for each subject will be inventoried and accounted for throughout the early access program.

The investigator should monitor, as per clinical practice, the standard-of-care medications (Peg-IFN-alfa and RBV) for compliance with dosing.

8. PRESTUDY AND CONCOMITANT THERAPY

The use of all therapy for hepatitis C infection (eg, Peg-IFN-alfa, RBV), prior to and during the early access program, should be recorded in the case report form (CRF).

Use of all therapy (prescription and over-the-counter) for rash and anemia administered after the first use of telaprevir until 30 days after administration of the last dose of telaprevir should be recorded on the appropriate pages of the CRF. See Section 9.3.1.1.2 for additional information on allowed and prohibited concomitant therapies for treatment of rash.

For serious adverse events, concomitant medications will be recorded in the CRF.

For therapies listed above, the therapy, start and stops dates, dosing regimen, route of administration, and dosage changes will be recorded in the CRF. All other concomitant medication should be recorded in the subject's medical record.

Recommendations for Other Medicinal Products

Recommendations for other medicinal products, as a result of drug interactions with telaprevir, are provided in Attachment 2. These recommendations are based on either drug interaction studies or predicted interactions due to the expected magnitude of interaction and potential for serious adverse events or loss of efficacy. Attachment 2 is a comprehensive list of all recommendations, however, since co-infections with active hepatitis B and HIV are excluded, antiviral treatments are not allowed.

Concomitant therapy with any of the following should <u>not</u> be prescribed.

<u>CYP3A substrates with a narrow therapeutic index</u>

Telaprevir must not be administered concurrently with medicinal products with a narrow therapeutic window that are substrates of cytochrome P450 3A (CYP3A). Co-administration of telaprevir may increase the plasma concentration of these medicinal

products, which may lead to serious and/or life threatening adverse reactions such as cardiac arrhythmia (ie, amiodarone, astemizole, bepridil, cisapride, flecainide, pimozide, propafenone, quinidine, terfenadine), or peripheral vasospasm or ischemia (ie, dihydroergotamine, ergonovine, ergotamine, methylergonovine), or myopathy, including rhabdomylosis (ie, lovastatin, simvastatin), or prolonged or increased sedation or respiratory depression (ie, orally administered midazolam, triazolam), or hypotension, or cardiac arrhythmia (ie, alfuzosin and sildenafil for pulmonary arterial hypertension).

Telaprevir must not be administered concurrently with any Class I or III antiarrhythmics.

Rifampicin

Rifampicin reduces the telaprevir plasma AUC by approximately 92%. Therefore, telaprevir must not be co-administered with rifampicin.

St. John's wort (Hypericum perforatum)

Plasma concentrations of telaprevir can be reduced by concomitant use of the herbal preparation St. John's wort (*Hypericum perforatum*). Therefore, herbal preparations containing St. John's wort should not be combined with telaprevir.

The decision to administer a prohibited drug or treatment during telaprevir administration should be discussed with the sponsor's responsible physician for the early access program.

9. STUDY EVALUATIONS

9.1. Study Procedures

9.1.1. Overview

The Time and Events Schedule that follows the Synopsis summarizes the recommended frequency and timing of safety and other measurements applicable to this early access program.

Enrollment of subjects into this early access program will continue until telaprevir becomes available for reimbursement in the country in which a subject resides, or until September 2013, whichever occurs first, unless other guidelines applicable per local regulations. Subjects already enrolled in the early access program when enrollment is stopped should complete treatment (ie, telaprevir, Peg-IFN-alfa/RBV) and continue follow-up according to the protocol schedule.

Additional serum or urine pregnancy tests may be performed, as determined necessary by the investigator or as required by local regulation, to establish the absence of pregnancy throughout the early access program.

9.1.2. Screening Phase

All subjects must sign informed consent prior to conduct of any procedure for the early access program. Subjects must satisfy all the inclusion criteria and none of the exclusion criteria listed in Sections 4.1 and 4.2, respectively, to be enrolled in this early access program.

The time period between the screening visit and the baseline visit (Day 1) can be reduced provided all data are available to perform the baseline visit.

9.1.3. Open-Label Treatment Phase

Eligible subjects will be given study drug (telaprevir) and a prescription for Peg-IFN-alfa/RBV and will be instructed to start all medications on the same day (Day 1) and on how to take the medications throughout the open-label treatment phase (see Section 6, Dosage and Administration).

Subjects must have HCV RNA monitored at Week 4 and 12. The virologic response at Week 4 and 12 will be used to determine the total treatment duration (24 or 48 weeks) of Peg-IFN-alfa/RBV for subjects with severe fibrosis (Metavir F3 or Ishak 3-4) who are treatment naïve or prior treatment relapsers. Previously treated subjects with prior partial or prior null response, or who had viral breakthrough, with severe fibrosis (Metavir F3 or Ishak 3-4) and all subjects with cirrhosis (Metavir F4 or Ishak 5-6) will subsequently be treated with Peg-IFN-alfa/RBV for an additional 36 weeks (total treatment duration of 48 weeks). (See Section 3.1, Study Design.)

For all subjects, stopping rules based on virologic response (ie, HCV RNA levels) will be applied to ensure that telaprevir or Peg-IFN-alfa/RBV treatments are stopped if subjects have viral breakthrough or failure (see Section 3.1, Study Design).

Subjects who discontinue treatment early or withdraw early from the early access program should have an early treatment discontinuation/early withdrawal visit performed. These subjects should return for the posttreatment follow-up visit, unless informed consent for further follow-up has been withdrawn.

9.1.4. Posttreatment Phase (Follow-Up)

All subjects should have a posttreatment follow-up visit, including measurement of plasma HCV RNA levels, performed 24 weeks after the last administered dose of treatment (ie, telaprevir, Peg-IFN-alfa, or RBV) (eg, Week 48 for subjects receiving 24 weeks of treatment, Week 72 for subjects requiring 48 weeks of treatment, 24 weeks after last administered dose of treatment for subjects who discontinue treatment early or withdraw from the open-label treatment phase without withdrawing informed consent).

Investigators may recontact the subject to obtain long-term follow-up information to determine the subject's safety or survival status (see Section 16.2.3, Informed Consent).

9.2. Efficacy Evaluations

Although there are no formal efficacy evaluations in this early access program, plasma HCV RNA levels will be determined by the local laboratory and results will be used as the basis for treatment-guided therapy as presented in Section 3.1, Study Design.

9.3. Safety Evaluations

Safety evaluations will include clinical laboratory assessments performed by local laboratories, physical examinations, evaluation of vital signs, and the reporting of adverse events.

Any clinically significant abnormalities persisting at the end of the early access program/early withdrawal will be followed by the investigator until resolution or until a clinically stable endpoint is reached.

The following evaluations of safety and tolerability are recommended to be performed according to the time points provided in the Time and Events Schedule. If needed, extra visits and assessments can be planned at the discretion of the investigator in order to best manage the subject's hepatitis C treatment.

Adverse Events

At each visit from signing of the informed consent form onwards, the subject will be asked about any untoward medical occurrences. Adverse events will be followed by the investigator as specified in Section 12, Adverse Event Reporting.

The DAIDS criteria (see Attachment 3) will be used to grade adverse events except for rash which has protocol-specific definitions of severity grades provided in Section 9.3.1.1.1. Only adverse events, including laboratory abnormalities, as defined in Section 12.3.1, which occur between the first use of investigational product (telaprevir) and 30 days after administration of the last dose of telaprevir will be recorded in the case report form (CRF).

Clinical Laboratory Tests

Blood samples for serum chemistry and hematology should be collected at the screening visit and at the time points listed in the Time and Events Schedule to assess clinically significant laboratory abnormalities. The investigator must review the laboratory reports, document these reviews, and record clinically important changes, whether serious or non-serious, which meet the adverse event reporting requirements as detailed in Section 12.3.1, in the adverse event section of the CRF.

The following tests should be performed by the local laboratory:

- Hematology Panel

  -hemoglobin
  -white blood cell (WBC) count with differential
  -platelet count

- Serum Chemistry Panel

-sodium	-gamma-glutamyltransferase (GGT)
-potassium	-bilirubin
-magnesium	-alkaline phosphatase
-creatinine	-uric acid
-glucose	-calcium
-aspartate aminotransferase (AST)	-albumin
-alanine aminotransferase (ALT)	

- Thyroid-stimulating hormone (TSH) test is required at screening and Weeks 4 and 8. Additional TSH tests should be performed at the recommended time points (eg, Weeks 12, 24, 48, and at the posttreatment follow-up visit) or according to local regulations, as per the standard-of-care, as presented in the Time and Events Schedule.
- Serum or urine pregnancy testing for women of childbearing potential only. A serum (β-HCG) pregnancy test is required at the screening visit and a serum or urine pregnancy test is required before first dose of treatment to ensure that the women are not pregnant at the time of starting treatment. For male subjects with female partners of childbearing potential, pregnancy tests are strongly recommended. See Section 4.3, Prohibitions and Restrictions, for additional information on pregnancy testing.
- Serology (HIV antibody, hepatitis B surface antigen [HbsAg], if not performed in the past or subject to change at screening)

Electrocardiogram

An electrocardiogram (ECG) is recommended at the screening visit to assess clinically significant ECG abnormalities.

Vital Signs

Pulse and blood pressure should be measured at the screening visit and at all visits in the open-label treatment phase.

Physical Examination

Height should be measured at screening only, and weight should be measured at screening and all visits in the open-label treatment phase.

A complete physical examination should be performed at screening and should include a review of the following systems: head/neck/thyroid, eyes/ears/nose/throat, chest, lungs, heart, lymph nodes, abdomen, skin, musculoskeletal systems, and neurological systems. Other body systems (ie, anorectal, genital) should be examined if medically indicated. The screening physical examination should also include an eye examination, as per standard-of-care for Peg-IFN-alfa.

At all other visits up to 30 days after the last dose of telaprevir, physical examinations should be directed at detecting adverse events. Only those signs and symptoms that represent clinically important changes, whether serious or non-serious, meeting the criteria for reporting of adverse events in this early access program as described in Section 12.3.1, All Adverse Events, will be recorded in the CRF.

All physical examinations performed more than 30 days after the last dose of telaprevir should be completed according to clinical practice.

9.3.1. Specific Toxicities

9.3.1.1. Assessment, Treatment, Management, and Documentation of Rash

Management of rash throughout the treatment period of the early access program should always follow generally accepted medical standards. In addition, the procedures described in the following paragraphs for the assessment, treatment, and management of rash cases should be followed.

Subjects will be instructed to contact the site immediately upon noticing a skin reaction and should be evaluated in a timely manner.

9.3.1.1.1. Rash Assessment: Severity Grading

Rashes will not be graded according to the DAIDS scale; instead the grade and severity of rash events should be assigned using the criteria described in the remainder of this section. If there is any doubt about severity grading of a rash event, the investigator must consult with the monitor before taking any action.

For rash, all grades will be recorded on the rash pages in the CRF.

Grade 1, mild: Rash is defined as a localized skin eruption and/or a skin eruption with a limited distribution (eg, up to several isolated sites on the body), with or without associated pruritus. A mild rash will have no target lesions, no signs of systemic involvement, and no involvement of mucous membranes or signs of epidermal detachment.

Grade 2, moderate: Rash is defined as a diffuse skin eruption involving up to approximately 50% of the body surface area, with or without superficial skin peeling and/or pruritus. Mucosal inflammation without ulceration may be present. Note that mucosal conditions that are not related to skin events, such as aphthous ulcers, stomatitis, or oral lichen planus, should be well documented and not considered when the rash is graded. A moderate rash will have no target lesions or signs of epidermal detachment. Moderate systemic signs and symptoms, such as fever, joint pain, and/or eosinophilia, can be experienced with moderate morbilliform rash. Therefore the type of rash and a thorough assessment of the degree of any systemic signs and their temporal relation to the onset and/or its progression are necessary for rash grading.

Grade 3, severe: Rash is defined as a generalized rash involving over 50% of the body surface area, or rash presenting with any of the following characteristics:

- rash with vesicles or bullae;
- superficial ulceration of mucous membranes;
- epidermal detachment (full thickness epidermal necrosis and separation of epidermis from underlying dermis);
- atypical or typical target lesions;
- palpable purpura/non-blanching erythema;
- diagnosis of drug-related eosinophilia with systemic symptoms (DRESS), erythema multiforme (EM), or acute generalized exanthematous pustulosis (AGEP).

Rash with appearance of significant systemic signs or symptoms that are new and are considered related to the onset and/or progression of rash should be considered to be grade 3.

Grade 4, life-threatening: Diagnosis of generalized bullous eruption, Stevens-Johnson syndrome (SJS), or toxic epidermal necrolysis (TEN).

Determination of seriousness of skin rash events will follow the standard International Conference on Harmonisation (ICH) criteria for serious adverse events as described in Section 12.1.1.

9.3.1.1.2. Rash Treatment

Antihistamines and topical corticosteroids may provide symptomatic relief to subjects who develop rash and experience associated symptoms such as pruritus.

Treatment of rash throughout the treatment period of the early access program should always follow generally accepted medical standards. In addition, the following treatment recommendations during telaprevir dosing should be followed.

Permitted topical and systemic antihistaminic drugs allowed for use with caution for all grades of rash include diphenhydramine (Benadryl®), hydroxyzine, levocetirizine (Xyzal®), and desloratadine (Clarinex®). Subjects should be followed symptomatically for possible increased exposure and worsening of adverse events such as drowsiness, and should be advised not to drive or operate heavy machinery while receiving antihistamines. The following antihistamines are prohibited from Day -14 until 7 days after the last dose of telaprevir: astemizole, terfenadine, and loratadine (see Section 8 and Attachment 2).

Because immunosuppression due to systemic corticosteroids can cause elevations in HCV RNA levels and because of the suspected drug-drug interactions between telaprevir and systemic corticosteroids, the latter should be used only when clinically necessary and after other treatments and measures have been employed, when possible. A consultation with a dermatologist should be requested for advice if needed. Telaprevir, Peg-IFN-alfa, and RBV must be discontinued immediately and permanently if a subject receives treatment with systemic corticosteroids (see also Section 10.2).

Topical corticosteroid use is permitted, but should be limited to brief periods, eg, up to 2 weeks of continuous/regular use, and limited to use on up to 50% of the body surface. Cream or lotion preparations are strongly encouraged in this situation, due to a lower absorption potential. Use of gel or ointment preparations of topical corticosteroids is discouraged due to a relative higher absorption potential. Treatment of skin eruptions with investigational agents or use of approved drugs in an off-label manner is also discouraged.

Use of all therapy (prescription and over-the-counter) for rash administered after the first use of telaprevir until 30 days after administration of the last dose of telaprevir, should be recorded on the appropriate pages of the CRF.

9.3.1.1.3. Rash Management

All skin events should be evaluated together with other systemic symptoms and laboratory abnormalities.

Management of rash until 30 days after the last telaprevir dose (Week 16 or earlier) should follow generally accepted medical standards, taking into account the protocol defined procedures described hereafter and treatment recommendations during telaprevir dosing described in Section 9.3.1.1.2. Management of rash during the subsequent weeks of treatment (standard treatment) should be in accordance with prescribing information recommendations for Peg-IFN-alfa and RBV.

Note that subjects having a skin rash (any grade) that requires treatment with systemic corticosteroids must have telaprevir, Peg-IFN-alfa, and RBV discontinued immediately and permanently. The sponsor's physician responsible for the early access program should be notified immediately of any subject who has a skin rash that requires treatment with systemic corticosteroids. The rash pages in the CRF must be completed.

Subjects experiencing any skin reaction considered to be a serious adverse event during telaprevir administration must permanently discontinue telaprevir treatment.

9.3.1.1.3.1. Management of Grade 1 or 2 Rash

General recommendations: For subjects experiencing a grade 1 or 2 rash, medical management will be at the discretion of the investigator and should follow generally accepted medical standards. Drugs to help alleviate symptoms as described above may be employed. In addition, subjects experiencing skin rash or pruritus should be advised of other strategies to minimize the intensity or progression of their signs and symptoms (eg, limiting sun exposure and heat; baking soda or oatmeal baths; loose-fitting clothes). Rash associated with RBV may complicate the assessment of rash in subjects receiving telaprevir. In all cases, subjects should be closely monitored for any rash progression or worsening of signs or symptoms of systemic involvement, and should be followed until the rash has resolved completely (in some cases this may require follow-up beyond the planned Follow-up Visit).

Study drugs discontinuation: For subjects experiencing a grade 1 rash, discontinuation of telaprevir, Peg-IFN-alfa, and RBV is generally not necessary. However, for subjects experiencing a grade 2 rash that progresses or does not improve, discontinuation of telaprevir, Peg-IFN-alfa, and RBV should be considered. If discontinuation of treatment due to rash is necessary, telaprevir should be permanently discontinued first. If the rash does not improve, symptomatically or objectively, within 7 days following telaprevir discontinuation, RBV use should be interrupted. Interruption of RBV dosing may be done sooner if the rash worsens despite discontinuation of telaprevir. Peg-IFN-alfa may be continued unless interruption is medically indicated.

Resumption of treatment: telaprevir dosing cannot be restarted after having been discontinued due to an adverse event. Peg-IFN-alfa and/or RBV may be restarted, if interrupted due to rash, if there is improvement in the rash within 14 days following their respective discontinuation. RBV monotherapy is not allowed; therefore, if Peg-IFN-alfa and RBV dosing are interrupted and RBV is restarted, Peg-IFN-alfa must also be restarted.

9.3.1.1.3.2. Management of Grade 3 Rash

General recommendations: For subjects experiencing a grade 3 rash, the same general recommendations as for subjects experiencing grade 1 and grade 2 rashes apply.

Treatment discontinuation: In subjects experiencing a grade 3 rash, telaprevir should be discontinued immediately and permanently. If the rash does not improve, symptomatically or objectively, within 7 days following telaprevir discontinuation, RBV use should also be interrupted. Interruption of RBV dosing may be done sooner if the subject’s rash worsens despite discontinuation of telaprevir. Peg-IFN-alfa may be continued unless interruption is medically indicated. The investigator may discontinue telaprevir, Peg-IFN-alfa, and RBV simultaneously if clinically indicated.

However, any subject diagnosed with or suspected to have DRESS, EM, or AGEP must have telaprevir, Peg-IFN-alfa, and RBV discontinued immediately and permanently.

Resumption of treatment: telaprevir dosing cannot be restarted after having been discontinued due to grade 3 rash. Peg-IFN-alfa and/or RBV may be restarted, if interrupted due to grade 3 rash, if there is improvement in the rash within 14 days following the interruption. RBV monotherapy is not allowed; therefore, if Peg-IFN-alfa and RBV dosing are interrupted and RBV is restarted, Peg-IFN-alfa must also be restarted. Please note that any subject who discontinues treatment due to DRESS, EM, or AGEP should not resume telaprevir, Peg-IFN-alfa, or RBV.

Management: The sponsor’s physician responsible for the early access program should be notified immediately of any subject who is diagnosed with or suspected to have DRESS, EM, or AGEP. The rash pages in the CRF must be completed.

Referral to a dermatologist is recommended for all grade 3 rashes. The following laboratory tests are mandatory: the hematology tests (with differential) described in Section 9.3, ALT, AST, and creatinine. Any additional or subsequent laboratory testing should be performed as needed, eg, if rash is worsening or if significant laboratory abnormalities that are identified, require monitoring.

Close clinical follow-up and appropriate medical intervention should be instituted. Daily follow up, in person or by telephone, to monitor for progression of the event may be necessary from the onset of the event until improvement is observed. Additional visits should be performed (eg, each week or more often, as clinically appropriate). All subjects should be followed until rash has resolved completely.

9.3.1.1.3.3. Management of Grade 4 Rash

General recommendations: For subjects experiencing a grade 4 rash, the same general recommendations as for subjects experiencing grade 1, 2, and 3 rashes apply.

Treatment discontinuation: Subjects diagnosed with or suspected to have a skin rash that is considered life-threatening (grade 4 rash), including SJS and TEN, must have telaprevir, Peg-IFN-alfa, and RBV discontinued immediately and permanently.

Resumption of treatment: Any subject who discontinues telaprevir, Peg-IFN-alfa, and RBV due to a grade 4 rash cannot resume treatment.

Management: The sponsor’s physician responsible for the early access program should be notified immediately of any subject who is diagnosed with or suspected to have a skin rash that is considered life-threatening (grade 4 rash), including SJS and TEN. The rash pages in the CRF must be completed.

Referral to a dermatologist is recommended for all grade 4 rashes. The following laboratory tests are mandatory: the hematology tests (with differential) described in Section 9.3, ALT, AST, and creatinine. Any additional or subsequent laboratory testing should be performed as needed, eg, if rash is worsening or if significant laboratory abnormalities that are identified, require monitoring.

Close clinical follow-up and appropriate medical intervention should be instituted. Daily follow up, in person or by telephone, to monitor for progression of the event may be necessary from the onset of the event until improvement is observed. Additional visits should be performed (eg, each week or more often, as clinically appropriate). All subjects should be followed until rash has resolved completely.

9.3.1.2. Management of Anemia

Decreases in hemoglobin levels are commonly observed in the treatment of chronic HCV infection. Hemoglobin levels should therefore be assessed before treatment and at Weeks 2, 4, 8, and 12, and as clinically appropriate thereafter. In certain cases it may be indicated to follow these parameters more frequently; this will be at the discretion of the investigator and unscheduled visits can be performed.

If anemia develops during treatment, treatment modifications of RBV should be made in line with label recommendations. Note that if RBV is permanently discontinued for the management of anemia, telaprevir must also be permanently discontinued. Telaprevir dose reductions are prohibited and once telaprevir treatment is discontinued for the management of anemia, it may not be reinitiated.

Anemia which occurs between the first use of investigational product (telaprevir) and 30 days after administration of the last dose of telaprevir will be recorded in the CRF (see Section 12.3.1) according to DAIDS criteria (see Attachment 3).

All interventions for anemia should be recorded on the CRF as described in Section 8, Prestudy and Concomitant Therapy.

10. SUBJECT COMPLETION/WITHDRAWAL

10.1. Completion

A subject will be considered to have completed the early access program if he or she has completed assessments at Week 24 or 48, based on virologic response to treatment as measured by locally collected plasma HCV RNA levels and/or type of subject (treatment naïve or prior treatment relapsers, or previously treated with prior partial or prior null response, or who had viral breakthrough; and Metavir/Ishak score) as discussed in Section 3.1, Study Design.

10.2. Discontinuation of Treatment

If a subject's study treatment (telaprevir) must be discontinued before the end of the treatment regimen, this will not result in automatic withdrawal of the subject from the early access program.

A subject’s treatment (telaprevir, Peg-IFN-alfa, RBV) may be discontinued if:

1. an serious adverse event occurs
   (Note: treatment must be discontinued for skin reactions considered serious adverse events, as described in the table below);
2. they fail to comply with the protocol requirements or fail to cooperate with the investigator.

A subject’s treatment with telaprevir may be permanently discontinued if a subject develops a medical condition that requires concomitant therapy with a prohibited drug listed in Section 8.

Subjects must discontinue telaprevir and Peg-IFN-alfa/RBV or have their treatment modified for the reasons included in the table below.

Reasons for Treatment Discontinuation or Modification and Actions to be Taken

Reason for Discontinuation/Modification	Action
**Safety Reason**	
Grade 3 or 4 adverse event/toxicity at least possibly related to telaprevir, except for grade 3 elevations in ALT/AST, grade 3 or 4 GGT elevations, and grade 3 or 4 hemoglobin decreases (unless they are persistent)	• Permanently discontinue telaprevir and continue Peg-IFN-alfa and RBV.<br>• Permanently discontinue telaprevir and interrupt RBV or RBV and Peg-IFN-alfa if clinically indicated.<br>• Peg-IFN-alfa and/or RBV may be restarted if there is improvement in the adverse event/toxicity within 14 days following the interruption. RBV monotherapy is not allowed.
Treatment with systemic corticosteroids	• Permanently discontinue telaprevir, Peg-IFN-alfa, and RBV immediately.
Grade 3 rash (for DRESS, EM, and AGEP, see special instructions below)	• Permanently discontinue telaprevir immediately and continue Peg-IFN-alfa and RBV.<br>• Interrupt RBV if the rash does not improve within 7 days after telaprevir discontinuation and continue Peg-IFN-alfa unless interruption is medically indicated. The investigator may also discontinue telaprevir, Peg-IFN-alfa, and RBV simultaneously if clinically indicated.<br>• Peg-IFN-alfa and/or RBV may be restarted if there is improvement in the rash within 14 days following the interruption. RBV monotherapy is not allowed.
DRESS, EM, and AGEP	• Permanently discontinue telaprevir, Peg-IFN-alfa, and RBV immediately.
Grade 4 rash (ie, skin rash that is considered life-threatening), including SJS and TEN	• Permanently discontinue telaprevir, Peg-IFN-alfa, and RBV immediately.
Anemia<br>(please also refer to Section 9.3.1.2)	• The investigator should consider adjusting the RBV dose, per the product labelling of RBV.<br>• If RBV is permanently discontinued for the management of anemia, telaprevir must also be permanently discontinued.
Telaprevir safety reason other than the ones specified above[a]	• Permanently discontinue telaprevir and continue Peg-IFN-alfa and RBV (regardless of HCV RNA value).
**Inadequate virologic response**	
HCV RNA >100 IU/mL at Week 4	• Permanently discontinue telaprevir
HCV RNA >100 IU/mL at Week 12	• Permanently discontinue Peg-IFN-alfa and RBV
HCV RNA levels beyond Week 12	• Refer to the local prescribing information for Peg-IFN-alfa and RBV

[a] If in the best interest of the subject according to the investigator

Note: Telaprevir dose modifications are prohibited and once telaprevir treatment is discontinued because of one of the withdrawal criteria specified above, it may not be reinitiated. Dose modifications, interruptions, or discontinuations of Peg-IFN-alfa or RBV should be in accordance with the local prescribing information of Peg-IFN-alfa or RBV, respectively.

AGEP=acute generalized exanthematous pustulosis; ALT=alanine aminotransferase; AST=aspartate aminotransferase; DRESS=drug-related eosinophilia with systemic symptoms; EM=erythema multiforme; GGT=gamma-glutamyltransferase; Peg-IFN=pegylated interferon; RBV=ribavirin; SJS=Stevens-Johnson syndrome; TEN=toxic epidermal necrolysis.

The date and the reason for discontinuation must be noted on the CRF.

If a subject discontinues treatment before the end of the treatment phase, obtain early treatment discontinuation and follow-up assessments, unless informed consent for further follow-up has been withdrawn.

For all subjects, it is recommended that a posttreatment follow-up visit (including sampling for HCV RNA) be performed 24 weeks after the last administrated dose of treatment (ie, telaprevir, Peg-IFN-alfa, or RBV) (eg, Week 48 for subjects receiving

24 weeks of treatment, Week 72 for subjects requiring 48 weeks of treatment, 24 weeks after last administered dose of treatment for subjects who discontinue treatment early or withdraw from the open-label treatment phase without withdrawing informed consent).

Subjects who discontinue study drugs prematurely will be instructed to continue the contraception and pregnancy testing procedures outlined in Section 4.3, Prohibitions and Restrictions until 4 months (female subjects of childbearing potential) or 7 months (male subjects and their female partners of childbearing potential) after the last intake of RBV, depending on the local prescribing information.

10.3. Withdrawal From the Study

A subject will be withdrawn from the early access program for any of the following reasons:

- Lost to follow-up
- Withdrawal of consent
- The investigator considers, for efficacy or safety reasons, it is in the best interest of the subject
- Death

If a subject withdraws from the early access program before the end of the treatment phase, obtain early treatment discontinuation/early withdrawal and follow-up assessments, unless informed consent for further follow-up has been withdrawn.

If a subject is lost to follow-up, every reasonable effort must be made by the study site personnel to contact the subject and determine the reason for discontinuation/withdrawal. The measures taken to follow up must be documented in the source documents.

When a subject withdraws before completing the early access program, the reason for withdrawal is to be documented in the CRF and in the source document. Study drug assigned to the withdrawn subject may not be assigned to another subject.

11. STATISTICAL METHODS

11.1. Analysis Population

The intent-to-treat analysis population will be defined as all enrolled subjects who received at least 1 dose of telaprevir. The intent-to-treat analysis population will be used for all statistical analyses, unless otherwise specified.

11.2. Subject Information

Descriptive statistics will be provided for all subjects who receive at least 1 dose of telaprevir.

11.3. Efficacy Analyses

There are no efficacy assessments and as such no formal planned efficacy analyses in this early access program. The HCV RNA data will be summarized using descriptive statistics (n, percent).

11.4. Sample Size Determination

No formal sample size calculation has been performed. The trial is not designed to evaluate a specific statistical hypothesis but to provide subjects with early access to telaprevir. Therefore, the number of enrolled subjects is not based on statistical but rather practical considerations involving the expected prevalence of HCV infection in the included countries and the projected number of eligible subjects based on major eligibility criteria along with other available therapies. Nonetheless, safety and tolerability data will be summarized using descriptive statistics. The percentage of subjects having selected safety and tolerability events will be estimated and 95% confidence intervals (CIs) for these estimates will be derived. Although no formal statistical hypotheses regarding overall response rates will be tested, the following table provides some guidance on the precision of estimation as measured by the half-width of the derived 95% CIs. For example, if the expected percentage of subjects with a serious adverse event is 10%, the expected 95% confidence interval would be approximately (8.9%, 11.1%) with 3,000 subjects.

Expected Half-Width of 95% Confidence Intervals for Percentage of Subjects With an Event

	Number of Subjects		
**Estimated Event Rate (%)**	**2,000**	**3,000**	**4,000**
**10**	1.3	1.1	0.9
**20**	1.8	1.4	1.2
**30**	2.0	1.6	1.4
**40**	2.1	1.8	1.5
**50**	2.2	1.8	1.5

11.5. Safety Analyses

Safety analyses will be performed for descriptive purposes only. There will be no inferential statistical analyses. Although there is no control arm and statistical comparisons are not formally planned, multiple logistic regression and subgroup analyses may be used to assess the variability of selected safety parameters across potential prognostic factors including, but not limited to, demographic, treatment, and other baseline characteristics. These will be viewed as hypothesis-generating rather than hypothesis testing analyses. These methods will be described in the Statistical Analysis Plan which will be finalized prior to database lock.

There are no planned interim analyses of safety data. However, the safety data may be used for standard safety reporting required for health authority submissions or requests.

Adverse Events

The verbatim terms used in the CRF by investigators to identify adverse events will be coded using the Medical Dictionary for Regulatory Activities (MedDRA). All reported adverse events with onset during the treatment phase (ie, treatment-emergent adverse events, and adverse events that have worsened since baseline) will be included in the analysis. For each adverse event, the percentage of subjects who experience at least 1 occurrence of the given event will be summarized by treatment group.

Only adverse events as defined in Section 12.3.1, All Adverse Events, whether serious or non-serious, that occur between the first use of investigational product (telaprevir) and 30 days after administration of the last dose of telaprevir will be collected and summarized. Particular attention will be given to adverse events of special interest, eg, rash and anemia.

Summaries, listings, datasets, or subject narratives may be provided, as appropriate, for those subjects who die, who discontinue treatment due to an adverse event, or who experience a severe or a serious adverse event.

12. ADVERSE EVENT REPORTING

Timely, accurate, and complete reporting and analysis of safety information from clinical studies are crucial for the protection of subjects, investigators, and the sponsor, and are mandated by regulatory agencies worldwide. The sponsor has established Standard Operating Procedures in conformity with regulatory requirements worldwide to ensure appropriate reporting of safety information; all clinical studies conducted by the sponsor or its affiliates will be conducted in accordance with those procedures.

12.1. Definitions

12.1.1. Adverse Event Definitions and Classifications

Adverse Event

An adverse event is any untoward medical occurrence in a clinical study subject administered a medicinal (investigational or non-investigational) product. An adverse event does not necessarily have a causal relationship with the treatment. An adverse event can therefore be any unfavorable and unintended sign (including an abnormal finding), symptom, or disease temporally associated with the use of a medicinal (investigational or non-investigational) product, whether or not related to that medicinal (investigational or non-investigational) product. (Definition per ICH)

This includes any occurrence that is new in onset or aggravated in severity or frequency from the baseline condition, or abnormal results of diagnostic procedures, including laboratory test abnormalities.

Note: The sponsor collects adverse events starting with the first use of investigational product (refer to Section 12.3.1, All Adverse Events for time of last adverse event recording).

Serious Adverse Event

A serious adverse event based on ICH is any untoward medical occurrence that at any dose:

- Results in death
- Is life-threatening
  (The subject was at risk of death at the time of the event. It does not refer to an event that hypothetically might have caused death if it were more severe.)
- Requires inpatient hospitalization or prolongation of existing hospitalization
- Results in persistent or significant disability/incapacity
- Is a congenital anomaly/birth defect
- Is a suspected transmission of any infectious agent via a medicinal product
- Medically Important*

*Medical and scientific judgment should be exercised in deciding whether expedited reporting is also appropriate in other situations, such as important medical events that may not be immediately life threatening or result in death or hospitalization but may jeopardize the subject or may require intervention to prevent one of the other outcomes listed in the definition above. These should usually be considered serious.

Unlisted (Unexpected) Adverse Event/Reference Safety Information

An adverse event is considered unlisted if the nature or severity is not consistent with the applicable product reference safety information. For an investigational product, the expectedness of an adverse event will be determined by whether or not it is listed in the Investigator's Brochure. For non-sponsor medicinal products to be administered during this study (eg, combination therapy) with a marketing authorization, the expectedness of an adverse event will be determined by whether or not it is listed in the local prescribing information. Investigator’s should follow standard practice for reporting unlisted adverse events to the marketing authorization holder for the standard-of-care therapy (ie, Peg-IFN-alfa, RBV) administered with telaprevir in this expanded access program.

Associated With the Use of the Drug

An adverse event is considered associated with the use of the drug if the attribution is possibly related or related by the definitions listed in Section 12.1.2.

12.1.2. Attribution Definitions

Not Related

An adverse event which is not related to the use of the drug.

Unlikely Related

An adverse event for which an alternative explanation is more likely, eg, concomitant drug(s) or concomitant disease(s), and/or the relationship in time suggests that a causal relationship is unlikely.

Possibly Related

An adverse event which might be due to the use of the drug. An alternative explanation, eg, concomitant drug(s) or concomitant disease(s), is inconclusive. The relationship in time is reasonable; therefore the causal relationship cannot be excluded.

Related

An adverse event which might be due to the use of the drug. The relationship in time is suggestive, eg, confirmed by dechallenge. An alternative explanation is less likely, eg, concomitant drug(s) or concomitant disease(s).

12.2. Special Reporting Situations

Safety events of interest on a sponsor medicinal product (telaprevir) that may require expedited reporting and/or safety evaluation include, but are not limited to:

- Overdose of a sponsor medicinal product
- Suspected abuse/misuse of a sponsor medicinal product
- Inadvertent or accidental exposure to a sponsor medicinal product
- Medication error involving a sponsor product (with or without subject/patient exposure to the sponsor medicinal product, eg, name confusion)

Special reporting situations which occur between the first use of telaprevir and 30 days after administration of the last dose of telaprevir should be recorded in the CRF. Any special reporting situation that meets the criteria of a serious adverse event should be recorded on the serious adverse event page of the CRF.

12.2.1. Severity Criteria

Please refer to Section 9.3.1.1.1 (rash) and Attachment 3 (other adverse events).

12.3. Procedures

12.3.1. All Adverse Events

All adverse events, as defined below, which occur between the first use of investigational product (telaprevir) and 30 days after administration of the last dose of telaprevir will be recorded in the CRF. Serious adverse events, including those spontaneously reported to the investigator more than 30 days after the last dose of investigational product (telaprevir), must be reported using the Serious Adverse Event Form. The sponsor will

evaluate any safety information that is spontaneously reported by an investigator beyond the time frame specified in the protocol.

The DAIDS criteria will be used to grade all adverse events except for rash which has protocol-specific definitions of severity grades provided in Section 9.3.1.1.1.

The following adverse events will be recorded in the CRF:

- Adverse events of special interest
  - All rash and anemia events, regardless of DAIDS grade or relationship to telaprevir
- The following which are considered <u>at least possibly related to telaprevir</u> by the investigator
  - All adverse events, including laboratory abnormalities, with DAIDS grade 2 (moderate) or higher
  - Other adverse events (ie, DAIDS grade 1 [mild]), including laboratory abnormalities, considered medically significant (eg, require medical intervention)
- All adverse events leading to discontinuation of telaprevir, regardless of DAIDS grade or relationship to telaprevir
- All serious adverse events, regardless of relationship to telaprevir
- Any pregnancy in female subjects or in female partners of male subjects; see Section 12.3.3
- Special reporting situations: overdose, suspected abuse/misuse, inadvertent or accidental exposure, or medication error of telaprevir; see Section 12.2
- Other adverse events will be recorded as per local regulations only

All adverse events, as defined above, must be recorded using medical terminology in the source document and the CRF. Whenever possible, diagnoses should be given when signs and symptoms are due to a common etiology (eg, cough, runny nose, sneezing, sore throat, and head congestion should be reported as "upper respiratory infection"). Investigators must record in the CRF their opinion concerning the relationship of the adverse event to study therapy. All measures required for adverse event management must be recorded in the source document and reported according to sponsor instructions.

The sponsor assumes responsibility for appropriate reporting of adverse events to the regulatory authorities. The sponsor will also report to the investigator all serious adverse events that are unlisted (unexpected) and associated with the use of the study drug (telaprevir). The investigator (or sponsor where required) must report these events to the appropriate Independent Ethics Committee/Institutional Review Board (IEC/IRB) that approved the protocol unless otherwise required and documented by the IEC/IRB.

12.3.2. Serious Adverse Events

All serious adverse events occurring during clinical studies must be reported to the appropriate sponsor contact person by investigational staff within 24 hours of their knowledge of the event.

Information regarding serious adverse events will be transmitted to the sponsor using the Serious Adverse Event Form, which must be completed and signed by a member of the investigational staff, and transmitted to the sponsor within 24 hours. The initial and follow-up reports of a serious adverse event should be made by facsimile (fax).

All serious adverse events that have not resolved by the end of the early access program, or that have not resolved upon discontinuation of the subject’s participation in the early access program, must be followed until any of the following occurs:

- The event resolves
- The event stabilizes
- The event returns to baseline, if a baseline value/status is available
- The event can be attributed to agents other than the study drug or to factors unrelated to the conduct of the early access program
- It becomes unlikely that any additional information can be obtained (subject or health care practitioner refusal to provide additional information, lost to follow-up after demonstration of due diligence with follow-up efforts)

Suspected transmission of an infectious agent by a medicinal product will be reported as a serious adverse event. Any event requiring hospitalization (or prolongation of hospitalization) that occurs during the course of a subject’s participation in this early access program must be reported as a serious adverse event, except hospitalizations for the following:

- Conditions which existed prior to the trial and which did not deteriorate during the trial
- Treatment or care of hepatitis C diseases unless fatal or considered to be related to telaprevir
- Investigational or elective procedures

Hepatitis C infection is characterized by a series of infections and neoplasms, which depending on the disease stage of the subject may constitute a life-threatening event. Reporting all such events as serious adverse events could divert attention from the serious adverse events that could be related to the study drug. Therefore for this early access

program the following occurrences will NOT be recorded or reported as serious adverse events:

- Any condition which occurred prior to the subject being enrolled in the early access program and did NOT worsen during the trial. These conditions should be recorded at the screening visit.
- Hepatitis C related events, unless fatal or judged to be associated with the use of telaprevir

The cause of death of a subject in a clinical study within 30 days of last dose of telaprevir, whether or not the event is expected or associated with the investigational agent, is considered a serious adverse event.

12.3.3. Pregnancy

Pregnancy which occurs between the first use of investigational product (telaprevir) and 30 days after administration of the last dose of telaprevir will be reported.

All initial reports of pregnancy must be reported to the sponsor by the investigational staff within 24 hours of their knowledge of the event using the appropriate pregnancy notification form. Abnormal pregnancy outcomes (eg, spontaneous abortion, stillbirth, and congenital anomaly) are considered serious adverse events and must be reported using the Serious Adverse Event Form. Any subject who becomes pregnant during this early access program must discontinue further treatment with telaprevir.

Because the study drug may have an effect on sperm, or if the effect is unknown, pregnancies in partners of male subjects included in this early access program will be reported by the investigational staff within 24 hours of their knowledge of the event using the appropriate pregnancy notification form.

Follow-up information regarding the outcome of the pregnancy and any postnatal sequelae in the infant will be required.

12.4. Contacting Sponsor Regarding Safety

The names (and corresponding telephone numbers) of the individuals who should be contacted regarding safety issues or questions regarding the early access program are listed on the Contact Information page(s), which will be provided as a separate document.

13. PRODUCT QUALITY COMPLAINT HANDLING

A product quality complaint (PQC) is defined as any suspicion of a product defect related to manufacturing, labeling, or packaging, ie, any dissatisfaction relative to the identity, quality, durability, or reliability of a product, including its labeling or package integrity.

The product in this early access program is telaprevir. PQCs may have an impact on the safety and efficacy of the product. Timely, accurate, and complete reporting and analysis of PQC information from clinical studies are crucial for the protection of subjects, investigators, and the sponsor, and are mandated by regulatory agencies worldwide. The sponsor has established procedures in conformity with regulatory requirements worldwide to ensure appropriate reporting of PQC information; all clinical studies conducted by the sponsor or its affiliates will be conducted in accordance with those procedures.

13.1. Procedures

All initial PQCs must be reported to the sponsor by the investigational staff as soon as possible after being made aware of the event.

If the defect is combined with a serious adverse event, the investigational staff must report the PQC to the sponsor according to the serious adverse event reporting timelines (refer to Section 12.3.2, Serious Adverse Events). A sample of the suspected product should be maintained for further investigation if requested by the sponsor.

13.2. Contacting Sponsor Regarding Product Quality

The names (and corresponding telephone numbers) of the individuals who should be contacted regarding product quality issues are listed on the Contact Information page(s), which will be provided as a separate document.

14. STUDY DRUG INFORMATION

Telaprevir will be provided by the sponsor. Standard-of-care treatment (Peg-IFN-alfa and RBV) will be prescribed by the investigator and will not be provided by the sponsor.

14.1. Physical Description of Study Drug(s)

Manufacturing, packaging, and labeling of telaprevir tablets will be done under the responsibility of the sponsor.

The telaprevir supplied for this early access program is formulated as a caplet-shaped yellow film-coated tablet for oral administration, containing 375 mg of telaprevir. Refer to the Investigator's Brochure[1] for a list of excipients.

14.2. Packaging

Telaprevir (375 mg) tablets will be packaged in high-density polyethylene (HDPE) bottles, each containing 42 film-coated tablets and fitted with a polypropylene (PP) child resistant closure and induction seal liner. A desiccant(s) will be added.

All medication must remain in the original packaging. No medication can be repackaged without prior approval from the sponsor.

14.3. Labeling

Study drug labels will contain information to meet the applicable regulatory requirements.

No medication can be relabeled without prior approval from the sponsor.

14.4. Preparation, Handling, and Storage

Telaprevir should be stored in the original bottle. Keep the bottle tightly closed in order to protect from moisture. Do not remove the desiccant.

14.5. Drug Accountability

The investigator is responsible for ensuring that all study drug (telaprevir) received at the site is inventoried and accounted for throughout the early access program. The dispensing of study drug to the subject, and the return of study drug from the subject (if applicable), must be documented on the drug accountability form. Subjects must be instructed to return all original containers, whether empty or containing study drug. Study drug returned by subjects participating in this early access program will be stored and disposed of according to the sponsor's instructions. Site staff must not combine contents of the study drug containers.

Study drug must be handled in strict accordance with the protocol and the container label, and must be stored at the study site in a limited-access area or in a locked cabinet under appropriate environmental conditions. Unused study drug, and study drug returned by the subject, must be available for verification by the sponsor's site monitor during on-site monitoring visits. The return to the sponsor of unused study drug, or used returned study drug for destruction, will be documented on the drug return form. When the site is an authorized destruction unit and study drug supplies are destroyed on site, this must also be documented on the drug return form.

Study drug should be dispensed under the supervision of the investigator or a qualified member of the investigational staff, or by a hospital/clinic pharmacist. Study drug will be supplied only to subjects participating in the early access program. Returned study drug must not be dispensed again, even to the same subject. Whenever a subject brings his or her study drug to the site for pill count, this is not seen as a return of supplies. Study drug may not be relabeled or reassigned for use by other subjects. The investigator agrees neither to dispense the study drug from, nor store it at, any site other than the study sites agreed upon with the sponsor.

15. STUDY-SPECIFIC MATERIALS

The investigator will be provided with the following supplies:

- Telaprevir Investigator's Brochure

16. ETHICAL ASPECTS

16.1. Study-Specific Design Considerations

The population chosen for this early access program includes subjects with advanced hepatic fibrosis or cirrhosis (Metavir F3, F4; Ishak stage 3 to 6) who are treatment naïve, prior treatment relapsers, or previously treated subjects with prior partial or prior null response, or who had viral breakthrough; see Section 1.2, Overall Rationale for the Study, for additional information. In this difficult-to-treat population with limited treatment options, making telaprevir available for use along with the standard-of-care may reduce the risk of long-term complications associated with HCV infection.

Subjects will have HCV RNA monitored throughout their participation in this early access program to closely monitor for viral breakthrough or virologic failure to limit evolution of resistant variants, as discussed in Section 3.2.5, Treatment Modifications Based on Virologic Response.

Potential subjects will be fully informed of the risks and requirements of the early access program and, during the early access program, subjects will be given any new information that may affect their decision to continue participation. They will be told that their consent to participate in the early access program is voluntary and may be withdrawn at any time with no reason given and without penalty or loss of benefits to which they would otherwise be entitled. Only subjects who are fully able to understand the risks, benefits, and potential adverse events of the early access program, and provide their consent voluntarily will be enrolled.

16.2. Regulatory Ethics Compliance

16.2.1. Investigator Responsibilities

The investigator is responsible for ensuring that the early access program is performed in accordance with the protocol, current ICH guidelines on GCP, and applicable regulatory and country-specific requirements.

Good Clinical Practice is an international ethical and scientific quality standard for designing, conducting, recording, and reporting studies that involve the participation of human subjects. Compliance with this standard provides public assurance that the rights, safety, and well being of study subjects are protected, consistent with the principles that originated in the Declaration of Helsinki, and that the clinical study data are credible.

16.2.2. Independent Ethics Committee or Institutional Review Board

Before the start of the early access program, the investigator (or sponsor where required) will provide the IEC/IRB with current and complete copies of the following documents:

- Final protocol and, if applicable, amendments
- Sponsor-approved informed consent form (and any other written materials to be provided to the subjects)
- Investigator's Brochure (or equivalent information) and amendments/addenda
- Sponsor-approved subject recruiting materials, if applicable
- Information on compensation for study-related injuries or payment to subjects for participation in the early access program, if applicable
- Investigator's curriculum vitae or equivalent information (unless not required, as documented by the IEC/IRB)
- Information regarding funding, name of the sponsor, institutional affiliations, other potential conflicts of interest, and incentives for subjects
- Any other documents that the IEC/IRB requests to fulfill its obligation

This early access program will be undertaken only after the IEC/IRB has given full approval of the final protocol, amendments (if any), the informed consent form, applicable recruiting materials, and subject compensation programs, and the sponsor has received a copy of this approval. This approval letter must be dated and must clearly identify the IEC/IRB and the documents being approved.

During the early access program the investigator (or sponsor where required) will send the following documents and updates to the IEC/IRB for their review and approval, where appropriate:

- Protocol amendments
- Revision(s) to informed consent form and any other written materials to be provided to subjects
- If applicable, new or revised subject recruiting materials approved by the sponsor
- Revisions to compensation for study-related injuries or payment to subjects for participation in the early access program, if applicable
- New edition(s) of the Investigator's Brochure and amendments/addenda
- Summaries of the status of the early access program at intervals stipulated in guidelines of the IEC/IRB (at least annually)
- Reports of adverse events that are serious, unlisted/unexpected, and associated with the investigational drug and concomitantly administered medicinal products (ie, Peg-IFN-alfa and RBV)

- New information that may adversely affect the safety of the subjects or the conduct of the early access program
- Deviations from or changes to the protocol to eliminate immediate hazards to the subjects
- Report of deaths of subjects under the investigator's care
- Notification if a new investigator is responsible for the early access program at the site
- Annual Safety Report and Line Listings, where applicable
- Any other requirements of the IEC/IRB

For all protocol amendments (excluding the ones that are purely administrative, with no consequences for subjects, data or trial conduct), the amendment and applicable informed consent form revisions must be submitted promptly to the IEC/IRB for review and approval before implementation of the change(s).

At least once a year, the IEC/IRB will be asked to review and reapprove this early access program. The reapproval should be documented in writing (excluding the ones that are purely administrative, with no consequences for subjects, data, or study conduct).

At the end of the early access program, the investigator (or sponsor where required) will notify the IEC/IRB about completion of the early access program.

16.2.3. Informed Consent

Each subject must give written consent according to local requirements after the nature of the early access program has been fully explained. The consent form must be signed before performance of any early access program-related activity. The consent form that is used must be approved by both the sponsor and by the reviewing IEC/IRB and be in a language that the subject can read and understand. The informed consent should be in accordance with principles that originated in the Declaration of Helsinki, current ICH and GCP guidelines, applicable regulatory requirements, and sponsor policy.

Before enrollment in the early access program, the investigator or an authorized member of the investigational staff must explain to potential subjects the aims, methods, reasonably anticipated benefits, and potential hazards of the early access program, and any discomfort participation in the early access program may entail. Subjects will be informed that their participation is voluntary and that they may withdraw consent to participate at any time. They will be informed that choosing not to participate will not affect the care the subject will receive for the treatment of his or her disease. Subjects will be told that alternative treatments are available if they refuse to take part and that such

refusal will not prejudice future treatment. Finally, they will be told that the investigator will maintain a subject identification register for the purposes of long-term follow-up if needed and that their records may be accessed by health authorities and authorized sponsor staff without violating the confidentiality of the subject, to the extent permitted by the applicable law(s) or regulations. By signing the informed consent form the subject is authorizing such access, and agrees to allow his or her study physician to recontact the subject for the purpose of obtaining consent for additional safety evaluations, if needed, or to obtain information about his or her survival status.

The subject will be given sufficient time to read the informed consent form and the opportunity to ask questions. After this explanation and before entry into the early access program, consent should be appropriately recorded by means of the subject's personally dated signature. After having obtained the consent, a copy of the informed consent form must be given to the subject.

If the subject is unable to read or write, an impartial witness should be present for the entire informed consent process (which includes reading and explaining all written information) and should personally date and sign the informed consent form after the oral consent of the subject is obtained.

16.2.4. Privacy of Personal Data

The collection and processing of personal data from subjects enrolled in this early access program will be limited to those data that are necessary to fulfill the objectives of the early access program.

These data must be collected and processed with adequate precautions to ensure confidentiality and compliance with applicable data privacy protection laws and regulations. Appropriate technical and organizational measures to protect the personal data against unauthorized disclosures or access, accidental or unlawful destruction, or accidental loss or alteration must be put in place. Sponsor personnel whose responsibilities require access to personal data agree to keep the identity of subjects in this early access program confidential.

The informed consent obtained from the subject includes explicit consent for the processing of personal data and for the investigator to allow direct access to his or her original medical records for early access program-related monitoring, audit, IEC/IRB review, and regulatory inspection. This consent also addresses the transfer of the data to other entities and to other countries.

The subject has the right to request through the investigator access to his or her personal data and the right to request rectification of any data that are not correct or complete. Reasonable steps will be taken to respond to such a request, taking into consideration the nature of the request, the conditions of the early access program, and the applicable laws and regulations.

16.2.5. Country Selection

This early access program will only be conducted in those countries where the intent is to launch or otherwise help ensure access to the developed product, unless explicitly addressed as a specific ethical consideration in Section 16.1, Study-Specific Design Considerations.

17. ADMINISTRATIVE REQUIREMENTS

17.1. Protocol Amendments

Neither the investigator nor the sponsor will modify this protocol without a formal amendment by the sponsor. All protocol amendments must be issued by the sponsor, and signed and dated by the investigator. Protocol amendments must not be implemented without prior IEC/IRB approval, or when the relevant competent authority has raised any grounds for non-acceptance, except when necessary to eliminate immediate hazards to the subjects, in which case the amendment must be promptly submitted to the IEC/IRB and relevant competent authority. Documentation of amendment approval by the investigator and IEC/IRB must be provided to the sponsor or its designee. When the change(s) involves only logistic or administrative aspects of the early access program, the IRB (and IEC where required) only needs to be notified.

During the course of the early access program, in situations where a departure from the protocol is unavoidable, the investigator or other physician in attendance will contact the appropriate sponsor representative (see Contact Information page(s) provided separately). Except in emergency situations, this contact should be made before implementing any departure from the protocol. In all cases, contact with the sponsor must be made as soon as possible to discuss the situation and agree on an appropriate course of action. The data recorded in the CRF and source documents will reflect any departure from the protocol, and the source documents will describe this departure and the circumstances requiring it.

17.2. Regulatory Documentation

17.2.1. Regulatory Approval/Notification

This protocol and any amendment(s) must be submitted to the appropriate regulatory authorities in each respective country, if applicable. This early access program may not be initiated until all local regulatory requirements are met.

17.2.2. Required Prestudy Documentation

The following documents must be provided to the sponsor before shipment of study drug to the investigational site:

- Protocol and amendment(s), if any, signed and dated by the principal investigator
- A copy of the dated and signed written IEC/IRB approval of the protocol, amendments, informed consent form, any recruiting materials, and if applicable, subject compensation programs. This approval must clearly identify the specific protocol by title and number and must be signed by the chairman or authorized designee.
- Name and address of the IEC/IRB including a current list of the IEC/IRB members and their function, with a statement that it is organized and operates according to GCP and the applicable laws and regulations. If accompanied by a letter of explanation, or equivalent, from the IEC/IRB, a general statement may be substituted for this list. If an investigator or a member of the investigational staff is a member of the IEC/IRB, documentation must be obtained to state that this person did not participate in the deliberations or in the vote/opinion of the early access program.
- Regulatory authority approval or notification, if applicable
- Signed and dated statement of investigator (eg, Form FDA 1572), if applicable
- Documentation of investigator qualifications (eg, curriculum vitae)
- Completed investigator financial disclosure form from the principal investigator, where required
- Signed and dated clinical trial agreement, which includes the financial agreement
- Any other documentation required by local regulations

The following documents must be provided to the sponsor before enrollment of the first subject:

- Completed investigator financial disclosure forms from all clinical subinvestigators
- Documentation of subinvestigator qualifications (eg, curriculum vitae)
- Name and address of any local laboratory conducting tests for the early access program, and a dated copy of current laboratory normal ranges for these tests, if applicable
- Local laboratory documentation demonstrating competence and test reliability (eg, accreditation/license), if applicable.

17.3. Subject Identification, Enrollment, and Screening Logs

The investigator agrees to complete a subject identification and enrollment log to permit easy identification of each subject during and after the early access program. This document will be reviewed by the sponsor site contact for completeness.

The subject identification and enrollment log will be treated as confidential and will be filed by the investigator in the study file. To ensure subject confidentiality, no copy will be made. All reports and communications relating to the early access program will identify subjects by initials and assigned number only.

The investigator must also complete a subject-screening log, which reports on all subjects who were seen to determine eligibility for inclusion in the early access program.

17.4. Source Documentation

At a minimum, source documentation must be available for the following to confirm data collected in the CRF: subject identification, eligibility, and study identification; discussion of the early access program and date of informed consent; dates of visits; results of safety and efficacy parameters as required by the protocol; record of all adverse events; and follow-up of adverse events; concomitant medication; drug receipt/dispensing/return records; study drug administration information; and date of completion of the early access program, and reason for early discontinuation of study drug or withdrawal from the early access program, if applicable.

In addition, the author of an entry in the source documents should be identifiable.

At a minimum, the type and level of detail of source data available for a subject in the early access program should be consistent with that commonly recorded at the site as a basis for standard medical care. Specific details required as source data for the early access program will be reviewed with the investigator before the early access program and will be described in the monitoring guidelines (or other equivalent document).

Data that will be recorded directly into the CRF will be considered source data and will be specified in the CRF completion guidelines.

17.5. Case Report Form Completion

Case report forms are provided for each subject in printed or electronic format.

Electronic Data Capture (eDC) will be used for this early access program. The data for the early access program will be transcribed by study personnel from the source documents onto an electronic CRF, and transmitted in a secure manner to the sponsor within the timeframe agreed upon between the sponsor and the site. The electronic file will be considered to be the CRF. Worksheets may be used for the capture of some data to facilitate completion of the CRF. Any such worksheets will become part of the subjects’ source documentation. All data relating to the early access program must be recorded in CRFs prepared by the sponsor. Data must be entered into CRFs in English.

Designated site personnel must complete CRFs as soon as possible after a subject visit, and the forms should be available for review at the next scheduled monitoring visit.

The investigator must verify that all data entries in the CRFs are accurate and correct.

All CRF entries, corrections, and alterations must be made by the investigator or other authorized study-site personnel. If necessary, queries will be generated in the eDC tool. The investigator or an authorized member of the investigational staff must adjust the CRF (if applicable) and complete the query.

If corrections to a CRF are needed after the initial entry into the CRF, this can be done in 3 different ways:

- Site personnel can make corrections in the eDC tool at their own initiative or as a response to an auto query (generated by the eDC tool)
- Site manager can generate a query for resolution by the investigational staff
- Clinical data manager can generate a query for resolution by the investigational staff

17.6. Data Quality Assurance/Quality Control

Steps to be taken to ensure the accuracy and reliability of data include the selection of qualified investigators and appropriate study sites, review of protocol procedures with the investigator and associated personnel before the early access program, and periodic monitoring visits by the sponsor.

Guidelines for CRF completion will be provided and reviewed with study personnel before the start of the early access program.

The sponsor will review CRFs for accuracy and completeness during on-site monitoring visits and after transmission to the sponsor; any discrepancies will be resolved with the investigator or designee, as appropriate. After upload of the data into the clinical study database they will be verified for accuracy and consistency with the data sources.

17.7. Record Retention

In compliance with the ICH/GCP guidelines, the investigator/institution will maintain all CRFs and all source documents that support the data collected from each subject, as well as all study documents as specified in ICH/GCP Section 8, Essential Documents for the Conduct of a Clinical Trial, and all study documents as specified by the applicable regulatory requirement(s). The investigator/institution will take measures to prevent accidental or premature destruction of these documents.

Essential documents must be retained until at least 2 years after the last approval of a marketing application in an ICH region and until there are no pending or contemplated marketing applications in an ICH region or until at least 2 years have elapsed since the formal discontinuation of clinical development of the investigational product. These documents will be retained for a longer period if required by the applicable regulatory requirements or by an agreement with the sponsor. It is the responsibility of the sponsor to inform the investigator/institution as to when these documents no longer need to be retained.

If the responsible investigator retires, relocates, or for other reasons withdraws from the responsibility of keeping the study records, custody must be transferred to a person who will accept the responsibility. The sponsor must be notified in writing of the name and address of the new custodian. Under no circumstance shall the investigator relocate or dispose of any study documents before having obtained written approval from the sponsor.

If it becomes necessary for the sponsor or the appropriate regulatory authority to review any documentation relating to this early access program, the investigator must permit access to such reports.

17.8. Monitoring

The sponsor will perform on-site monitoring visits as frequently as necessary. The monitor will record dates of the visits in a study site visit log that will be kept at the site. The first post-initiation visit will be made as soon as possible after enrollment has begun. At these visits, the monitor will compare the data entered into the CRFs with the hospital or clinic records (source documents). The nature and location of all source documents will be identified to ensure that all sources of original data required to complete the CRF are known to the sponsor and investigational staff and are accessible for verification by the sponsor site contact. If electronic records are maintained at the investigational site, the method of verification must be discussed with the investigational staff.

Direct access to source documentation (medical records) must be allowed for the purpose of verifying that the data recorded in the CRF are consistent with the original source data. Findings from this review of CRFs and source documents will be discussed with the investigational staff. The sponsor expects that, during monitoring visits, the relevant investigational staff will be available, the source documentation will be accessible, and a suitable environment will be provided for review of study-related documents. The monitor will meet with the investigator on a regular basis during the early access program to provide feedback on the conduct of the early access program.

17.9. Study Completion/Termination

17.9.1. Study Completion

The early access program is considered completed with the last visit for the last subject participating in the early access program. The final data from the investigational site will be sent to the sponsor (or designee) after completion of the final subject visit at that site, in the time frame specified in the Clinical Trial Agreement.

17.9.2. Study Termination

The sponsor reserves the right to close the investigational site or terminate the early access program at any time for any reason at the sole discretion of the sponsor. Investigational sites will be closed upon completion of the early access program. An investigational site is considered closed when all required documents and study supplies have been collected and a site closure visit has been performed.

The investigator may initiate site closure at any time, provided there is reasonable cause and sufficient notice is given in advance of the intended termination.

Reasons for the early closure of an investigational site by the sponsor or investigator may include but are not limited to:

- Failure of the investigator to comply with the protocol, the requirements of the IEC/IRB or local health authorities, the sponsor’s procedures, or GCP guidelines
- Inadequate recruitment of subjects by the investigator
- Discontinuation of further drug development.

17.10. On-Site Audits

Representatives of the sponsor’s clinical quality assurance department may visit the site at any time during or after completion of the early access program to conduct an audit of the early access program in compliance with regulatory guidelines and company policy. These audits will require access to all study records, including source documents, for inspection and comparison with the CRFs. Subject privacy must, however, be respected. The investigator and staff are responsible for being present and available for consultation during routinely scheduled site audit visits conducted by the sponsor or its designees.

Similar auditing procedures may also be conducted by agents of any regulatory body, either as part of a national GCP compliance program or to review the results of this early access program in support of a regulatory submission. The investigator should immediately notify the sponsor if they have been contacted by a regulatory agency concerning an upcoming inspection.

17.11. Use of Information and Publication

All information, including but not limited to information regarding telaprevir or the sponsor's operations (eg, patent application, formulas, manufacturing processes, basic scientific data, prior clinical data, formulation information) supplied by the sponsor to the investigator and not previously published, and any data generated as a result of this early access program, are considered confidential and remain the sole property of the sponsor. The investigator agrees to maintain this information in confidence and use this information only to accomplish this early access program, and will not use it for other purposes without the sponsor's prior written consent.

The investigator understands that the information developed in the early access program will be used by the sponsor in connection with the continued development of telaprevir, and thus may be disclosed as required to other clinical investigators or regulatory agencies. To permit the information derived from the clinical studies to be used, the investigator is obligated to provide the sponsor with all data obtained in the early access program.

The results of the early access program will be reported in a Clinical Study Report generated by the sponsor and will contain CRF data from all investigational sites that participated in the early access program. Recruitment performance or specific expertise related to the nature and the key assessment parameters of the early access program will be used to determine a coordinating investigator. Any work created in connection with performance of the early access program and contained in the data that can benefit from copyright protection (except any publication by the investigator as provided for below) shall be the property of the sponsor as author and owner of copyright in such work.

The sponsor shall have the right to publish such data and information without approval from the investigator. If an investigator wishes to publish information from the early access program, a copy of the manuscript must be provided to the sponsor for review at least 60 days before submission for publication or presentation. Expedited reviews will be arranged for abstracts, poster presentations, or other materials. If requested by the sponsor in writing, the investigator will withhold such publication for up to an additional 60 days to allow for filing of a patent application. In the event that issues arise regarding scientific integrity or regulatory compliance, the sponsor will review these issues with the investigator. The sponsor will not mandate modifications to scientific content and does not have the right to suppress information. For multicenter study designs and substudy approaches, secondary results generally should not be published before the primary endpoints of a study have been published. Similarly, investigators will recognize the integrity of a multicenter study by not submitting for publication data derived from the

individual site until the combined results from the completed study have been submitted for publication, within 12 months of the availability of the final data (tables, listings, graphs), or the sponsor confirms there will be no multicenter study publication. Authorship of publications resulting from this early access program will be based on the guidelines on authorship, such as those described in the Uniform Requirements for Manuscripts Submitted to Biomedical Journals, which state that the named authors must have made a significant contribution to the design of the early access program or analysis and interpretation of the data, provided critical review of the paper, and given final approval of the final version.

Registration of Clinical Studies and Disclosure of Results

The sponsor will register and/or disclose the existence of and the results of clinical studies as required by law.

REFERENCES

1. Telaprevir (VX-950) Investigator’s Brochure Version 11. Document ID No. EDMS-ERI-4497699 Vertex Pharmaceuticals Incorporated and Tibotec BVBA (8 June 2010).
2. Alter HJ, Houghton M. Clinical Medical Research Award. Hepatitis C virus and eliminating post-transfusion hepatitis. Nat Med 2000;6(10):1082-1086.
3. WHO. Global surveillance and control of hepatitis C. Report of a WHO Consultation organized in collaboration with the Viral Hepatitis Prevention Board, Antwerp, Belgium. J Viral Hepat 1999;6:35-47.
4. Strickland GT, El-Kamary SS, Klenerman P, Nicosia A. Hepatitis C vaccine: supply and demand. Lancet Infect Dis 2008;8(6):379-386.
5. Guideline on the clinical evaluation of direct acting antiviral agents intended for treatment of chronic hepatitis C (EMEA/CHMP/EWP/30039/2008). http://www.emea.europa.eu/pdfs/human/ewp/3003908en.pdf, accessed on 10 September 2009.
6. Hoofnagle JH. Course and outcome of hepatitis C. Hepatol 2002;36:S211-S29.
7. Di Bisceglie AM. Natural history of hepatitis C: its impact on clinical management. Hepatol 2000;31(4):1014-1018.
8. Fattovich G, et al. Morbidity and mortality in compensated cirrhosis type C: A retrospective follow-up study of 384 patients. Gastroenterol 1997;112(2):463-472.
9. Serfaty L, et al. Determinants of outcomes of compensated hepatitis C virus-related cirrhosis. Hepatol 1998;27(5):1435-1440.
10. Hu KQ, Tong MJ. The long-term outcomes of patients with compensated hepatitis C virus-related cirrhosis and history of parenteral exposure in the United States. Hepatol 1999;29(4):1311-1316.
11. Lee WM, Dienstag JL, Lindsay KL, et al. Evolution of the HALT-C Trial: pegylated interferon as maintenance therapy for chronic hepatitis C in previous interferon nonresponders. Control Clin Trials 2004;25(5):472-492.
12. Di Bisceglie AM, Shiffman ML, Everson GT, et al. Prolonged therapy of advanced chronic hepatitis C with low-dose peginterferon. N Engl J Med 2008;359:2429-2441.
13. Mallet V. Brief Communication: The relationship of regression of cirrhosis to outcome in chronic Hepatitis C. Ann Intern Med 2008;149(6):399-403.
14. Black M, Thomas R. Antiviral treatment for Hepatitis C cirrhosis: Is the effort justified? Ann Intern Med 2008;149(6):427-428.
15. Aghemo A, Colombo M. Cirrhosis regression in chronic Hepatitis C: an old tale with a new ending. Gastroenterol 2009;136(4):1447-1449.
16. Nelson DR, Davis GL, Jacobson I, et al. Hepatitis C virus: a critical appraisal of approaches to therapy. Clin Gastroenterol Hepatol 2009;7:397-414.
17. McHutchison JG, Lawitz EJ, Shiffman ML, et al for the IDEAL Study Team. Peginterferon alfa-2b or alfa-2a with ribavirin for treatment of hepatitis C infection. N Engl J Med 2009;361(6):580-593.
18. Hadziyannis SJ et al, Peginterferon alfa-2a and ribavirin combination therapy in chronic hepatitis C. Ann Int Med 2004;140:346-355.
19. Jensen DM, Marcellin P, Freillich B, et al. Re-treatment of patients with chronic hepatitis C who do not respond to peginterferon-α2b. Ann Intern Med 2009;150:528-540.
20. Poynard T, Colombo M, Bruix J, et al. Peginterferon alfa-2b and ribavirin: effective in patients with hepatitis C who failed interferon alfa/ribavirin therapy. Gastroenterol 2009;136(5):1618-1628.
21. Shiffman ML, Di Bisceglie AM, Lindsay KL, et al. for the HALT-C Trial Group. Peinterferon alfa-2a and ribavirin in patients with chronic Hepatitis C who have failed prior treatment. Gastroenterol 2004;126:1015-1023.

22. Everson GT, Hoefs JC, Seeff LB, et al. for the HALT-C Trial Group. Impact of disease severity on outcome of antiviral therapy for chronic hepatitis C: Lessons from the HALT-C trial. Hepatol 2006;44(6):1675-1684.

23. 75% of Treatment-Naïve Patients with Chronic Hepatitis C Achieve SVR (Viral Cure) with Telaprevir-Based Treatment in Phase 3 Trial [news release]. Cambridge, MA: Vertex Pharmaceuticals Inc; May 25, 2010. http://investors.vrtx.com/releasedetail.cfm?ReleaseID=473342. Accessed August 27, 2010.

24. McHutchison JG, Jacobson IM, Dusheiko GM, et al for the ADVANCE study team. Telaprevir in combination with peginterferon and ribavirin in genotype 1 HCV treatment-naïve patients: final results of Phase 3 ADVANCE study. Oral Communication at: 61st Annual Meeting of the American Association for the Study of Liver Diseases; October 29 - November 2, 2010; Boston, MA.

25. 65% of People Whose Prior Treatment for Hepatitis C Was Unsuccessful Achieved SVR (Viral Cure) with Telaprevir-Based Therapy in Phase 3 REALIZE Study [news release]. Cambridge, MA: Vertex Pharmaceuticals Inc; September 7, 2010. http://investors.vrtx.com/releasedetail.cfm?ReleaseID=505239. Accessed October 18, 2010.

26. Report D224. Viral dynamic model-based simulations of varying telaprevir dosage and duration, and varying duration of peg-interferon and ribavirin co-administration, during clinical studies. Vertex Pharmaceutical Inc (October 2007).

27. Pegasys [Summary of Product Characteristics]. United Kingdom: Roche Ltd; 2010.

Attachment 1:
Child-Pugh Classification of Severity of Liver Disease

	Points Scored for Increasing Abnormality		
Clinical and Biochemical Measurements	1	2	3
Encephalopathy (grade)[a]	None	1 and 2	3 and 4
Ascites[b]	Absent	Slight	Moderate
Bilirubin (mg per 100 mL)	1 - 2	2 - 3	> 3
Albumin (g per 100 mL)	3.5	2.8 - 3.5	< 2.8
Prothrombin time (sec. prolonged)	1 - 4	4 - 6	> 6

[a] According to grading of Trey, Burns and Saunders (1966).
[b] As determined by physical examination alone.

1, 2 or 3 points are scored for increasing abnormality of each of the 5 parameters measured.

Grade A: Total score of 5 or 6

Grade B: Total score of 7 to 9

Grade C: Total score of 10 to 15

Attachment 2:

Interactions and Dose Recommendations With Other Medicinal Products

This attachment provides dosing recommendations as a result of drug interactions with telaprevir. These recommendations are based on either drug interaction studies (indicated with *) or predicted interactions due to the expected magnitude of interaction and potential for serious adverse events or loss of efficacy.

The direction of the arrow (↑ = increase, ↓ = decrease, ↔ = no change) for each pharmacokinetic parameter is based on the 90% confidence interval of the geometric mean ratio being within (↔), below (↓) or above (↑) the 80% to 125% range.

INTERACTIONS AND DOSE RECOMMENDATIONS WITH OTHER MEDICINAL PRODUCTS

Medicinal products by therapeutic areas	Effect on concentration of telaprevir or concomitant medicinal product	Clinical Comment
**ANTIARRHYTHMICS**		
lidocaine<br>(systemic)	↑ lidocaine	Telaprevir may increase the concentrations of systemically administered lidocaine. Caution is warranted and clinical monitoring is recommended when co-administered with telaprevir.
digoxin*	↑ digoxin<br>AUC 1.85 (1.70-2.00)<br>C_{max} 1.50 (1.36-1.65)	Concentrations of digoxin were increased when co-administered with telaprevir. The lowest dose of digoxin should be initially prescribed. The serum digoxin concentrations should be monitored and used for titration of digoxin dose to obtain the desired clinical effect.
**ANTIBACTERIALS**		
clarithromycin<br>erythromycin<br>telithromycin<br>troleandomycin	↑ telaprevir<br>↑ antibacterials	Concentrations of both telaprevir and the antibacterial may be increased during co-administration. Caution is warranted and clinical monitoring is recommended when co-administered with telaprevir.<br>QT interval prolongation and Torsade de Pointes have been reported with clarithromycin and erythromycin. QT interval prolongation has been reported with telithromycin
**ANTICOAGULANT**		
warfarin	↑ or ↓ warfarin	Concentrations of warfarin may be altered when co-administered with telaprevir. It is recommended that the international normalized ratio (INR) be monitored when warfarin is co-administered with telaprevir.
**ANTICONVULSANTS**		
carbamazepine<br>phenobarbital<br>phenytoin	↓ telaprevir<br>↑ carbamazepine<br>↑ or ↓ phenytoin<br>↑ or ↓ phenobarbital	Concentrations of the anticonvulsant may be altered and concentrations of telaprevir may be decreased. Caution should be used when prescribing carbamazepine, phenobarbital, and phenytoin.<br>Telaprevir may be less effective in patients taking these agents concomitantly.<br>Clinical or laboratory monitoring of carbamazepine, phenobarbital, and phenytoin concentrations and dose titration are recommended to achieve the desired clinical response.
**ANTIDEPRESSANTS**		
escitalopram*	↔ telaprevir<br>↓ escitalopram<br>AUC 0.65 (0.60-0.70)<br>C_{max} 0.70 (0.65-0.76)<br>C_{min} 0.58 (0.52-0.64)	Concentrations of escitalopram were decreased when co-administered with telaprevir. Selective serotonin reuptake inhibitors such as escitalopram have a wide therapeutic index, but doses may need to be adjusted when combined with telaprevir.

(Continued)

Attachment 2: (Continued)
Interactions and Dose Recommendations With Other Medicinal Products

INTERACTIONS AND DOSE RECOMMENDATIONS WITH OTHER MEDICINAL PRODUCTS

**Medicinal products by therapeutic areas**	**Effect on concentration of telaprevir or concomitant medicinal product**	**Clinical Comment**
**ANTIDEPRESSANTS (continued)**		
desipramine<br>trazodone	↑ desipramine<br>↑ trazodone	Concomitant use of trazodone or desipramine and telaprevir may increase plasma concentrations of trazadone or desipramine which may lead to adverse events such as nausea, dizziness, hypotension, and syncope. If trazodone or desipramine is used with telaprevir, the combination should be used with caution and a lower dose of trazodone or desipramine should be considered.
**ANTIEMETICS**		
domperidone	↑ domperidone	Concentrations of domperidone may be increased when co-administered with telaprevir. Co-administration of domperidone with telaprevir should be avoided.
**ANTIFUNGALS**		
ketoconazole*<br>itraconazole<br>posaconazole<br>voriconazole	↑ ketoconazole<br>↑ telaprevir<br>AUC 1.62 (1.45-1.81)<br>C_{max} 1.24 (1.10-1.41)<br><br>↑ itraconazole<br>↑ posaconazole<br>↑ or ↓ voriconazole	Ketoconazole increases the plasma concentrations of telaprevir. Concomitant systemic use of itraconazole or posaconazole with telaprevir may increase plasma concentration of telaprevir.<br>Plasma concentrations of itraconazole, ketoconazole, or posaconazole may be increased in the presence of telaprevir. When co-administration is required, high doses of itraconazole (>200 mg/day) or ketoconazole (>200 mg/day) are not recommended. Caution is warranted and clinical monitoring is recommended for itraconazole, posaconazole, and voriconazole.<br>QT interval prolongation and Torsade de Pointes have been reported with voriconazole and posaconazole. QT interval prolongation has been reported with ketoconazole.<br>Due to multiple enzymes involved with voricanazole metabolism, it is difficult to predict the interaction with telaprevir. Voriconazole should not be administered to patients receiving telaprevir unless an assessment of the benefit/risk ratio justifies its use.
**ANTIGOUT**		
colchicine	↑ colchicine	Patients with renal or hepatic impairment should not be given colchicine with telaprevir, due to the risk of colchicine toxicity. A reduction in colchicine dosage or an interruption of colchicine treatment is recommended in patients with normal renal or hepatic function.<br><u>Treatment of gout flares: co-administration of colchicine in patients on telaprevir:</u><br>0.6 mg (1 tablet) for 1 dose, followed by 0.3 mg (half tablet) 1 hour later. Not to be repeated before 3 days.<br><u>If used for prophylaxis of gout flares: co-administration of colchicine in patients on telaprevir:</u><br>If the original regimen was 0.6 mg twice a day, the regimen should be adjusted to 0.3 mg once a day.<br>If the original regimen was 0.6 mg once a day, the regimen should be adjusted to 0.3 mg once every other day.<br><u>Treatment of familial Mediterranean fever (FMF):</u><br>co-administration of colchicine in patients on telaprevir: Maximum daily dose of 0.6 mg (may be given as 0.3 mg twice a day).

(Continued)

Attachment 2: (Continued)
Interactions and Dose Recommendations With Other Medicinal Products

INTERACTIONS AND DOSE RECOMMENDATIONS WITH OTHER MEDICINAL PRODUCTS

**Medicinal products by therapeutic areas**	**Effect on concentration of telaprevir or concomitant medicinal product**	**Clinical Comment**
**ANTIMYCOBACTERIAL**		
rifabutin	↓ telaprevir<br>↑ rifabutin	Concentrations of telaprevir may be decreased, while rifabutin concentrations may be increased during co-administration. Telaprevir may be less effective due to decreased concentrations. The concomitant use of rifabutin and telaprevir is not recommended.
**BENZODIAZEPINES**		
alprazolam*	↑ alprazolam<br>AUC 1.35 (1.23-1.49)<br>C_{max} 0.97 (0.92-1.03)	Concomitant use of alprazolam and telaprevir increase exposure to alprazolam by 35%. Clinical monitoring is warranted.
parenterally administered midazolam*	↑ midazolam<br>AUC 3.40 (3.04-3.79)<br>C_{max} 1.02 (0.80-1.31)	Concomitant use of parenterally administered midazolam with telaprevir increased exposure to midazolam 3.4-fold. Co-administration should be done in a setting which ensures clinical monitoring and appropriate medical management in case of respiratory depression and/or prolonged sedation.<br>Dose reduction for midazolam should be considered, especially if more than a single dose of midazolam is administered.<br>Co-administration of oral midazolam with telaprevir is contraindicated.
zolpidem (non-benzodiazepine sedative)*	↓ zolpidem<br>AUC 0.53 (0.45-0.64)<br>C_{max} 0.58 (0.52-0.66)	Exposure to zolpidem was decreased by 47% when co-administered with telaprevir. Clinical monitoring and dose titration of zolpidem is recommended to achieve the desired clinical response.
**CALCIUM CHANNEL BLOCKERS**		
amlodipine*	↑ amlodipine<br>AUC 2.79 (2.58-3.01)<br>C_{max} 1.27 (1.21-1.33)	Exposure to amlodipine was increased 2.8-fold when co-administered with telaprevir. Caution should be used and dose reduction for amlodipine should be considered. Clinical monitoring is recommended.
diltiazem<br>felodipine<br>nicardipine<br>nifedipine<br>nisoldipine<br>verapamil	↑ calcium channel blockers	Concentrations of other calcium channel blockers may be increased when telaprevir is co-administered.<br>Caution is warranted and clinical monitoring of patients is recommended.
**CORTICOSTEROIDS**		
Systemic<br>dexamethasone	↓ telaprevir	Systemic dexamethasone induces CYP3A and can thereby decrease telaprevir plasma concentrations. This may result in loss of therapeutic effect of telaprevir. Therefore this combination should be used with caution or alternatives should be considered.
inhaled/nasal<br>fluticasone<br>budesonide	↑ fluticasone<br>↑ budesonide	Concomitant use of inhaled fluticasone or budesonide and telaprevir may increase plasma concentrations of fluticasone or budesonide resulting in significantly reduced serum cortisol concentrations. Co-administration of fluticasone or budesonide and telaprevir is not recommended unless the potential benefit to the patient outweighs the risk of systemic corticosteroid side effects.

(Continued)

Attachment 2: (Continued)

Interactions and Dose Recommendations With Other Medicinal Products

INTERACTIONS AND DOSE RECOMMENDATIONS WITH OTHER MEDICINAL PRODUCTS

**Medicinal products by therapeutic areas**	**Effect on concentration of telaprevir or concomitant medicinal product**	**Clinical Comment**
**ENDOTHELIN RECEPTOR ANTAGONIST**		
bosentan	↑ bosentan	Concentrations of bosentan may be increased when co-administered with telaprevir. Caution is warranted and clinical monitoring is recommended.
**HIV-ANTIVIRAL AGENTS: HIV-PROTEASE INHIBITORS (PIs)**<br>**Note: For this protocol, since co-infections with HIV are excluded, antiviral treatments are not allowed.**		
atazanavir/ritonavir*	↓ telaprevir<br>AUC 0.80 (0.76-0.85)<br>C_{max} 0.79 (0.74-0.84)<br>C_{min} 0.85 (0.75-0.98)<br>↑ atazanavir<br>AUC 1.17 (0.97-1.43)<br>C_{max} 0.85 (0.73-0.98)<br>C_{min} 1.85 (1.40-2.44)	In a drug interaction study in healthy volunteers where telaprevir was co-administered with atazanavir/ritonavir, the steady-state telaprevir exposure was reduced by 20%, while the steady-state atazanavir exposure was increased by 17%.
darunavir/ritonavir*	↓ telaprevir<br>AUC 0.65 (0.61-0.69)<br>C_{max} 0.64 (0.61-0.67)<br>C_{min} 0.68 (0.63-0.74)<br>↓ darunavir<br>AUC 0.60 (0.57-0.63)<br>C_{max} 0.60 (0.56-0.64)<br>C_{min} 0.58 (0.52-0.63)	In a drug interaction study in healthy volunteers where telaprevir was co-administered with darunavir/ritonavir, the steady-state telaprevir exposure was reduced by 35%, while the steady-state darunavir exposure was reduced by 40%. It is not recommended to co-administer darunavir/ritonavir and telaprevir.
fosamprenavir/ritonavir*	↓ telaprevir<br>AUC 0.68 (0.63-0.72)<br>C_{max} 0.67 (0.63-0.71)<br>C_{min} 0.70 (0.64-0.77)<br>↓ amprenavir<br>AUC 0.53 (0.49-0.58)<br>C_{max} 0.65 (0.59-0.70)<br>C_{min} 0.44 (0.40-0.50)	In a drug interaction study in healthy volunteers where telaprevir was co-administered with fosamprenavir/ritonavir, the steady-state telaprevir exposure was reduced by 32%, while the steady-state amprenavir exposure was reduced by 47%. It is not recommended to co-administer fosamprenavir/ritonavir and telaprevir.
lopinavir/ritonavir*	↓ telaprevir<br>AUC 0.46 (0.41-0.52)<br>C_{max} 0.47 (0.41-0.52)<br>C_{min} 0.48 (0.40-0.56)<br>↔ lopinavir<br>AUC 1.06 (0.96-1.17)<br>C_{max} 0.96 (0.87-1.05)<br>C_{min} 1.14 (0.96-1.36)	In a drug interaction study in healthy volunteers where telaprevir was co-administered with lopinavir/ritonavir, the steady-state telaprevir exposure was reduced by 54%, while the steady-state exposure to lopinavir was not affected. It is not recommended to co-administer lopinavir/ritonavir and telaprevir.

(Continued)

Attachment 2: (Continued)

Interactions and Dose Recommendations With Other Medicinal Products

INTERACTIONS AND DOSE RECOMMENDATIONS WITH OTHER MEDICINAL PRODUCTS

**Medicinal products by therapeutic areas**	**Effect on concentration of telaprevir or concomitant medicinal product**	**Clinical Comment**
**HIV-ANTIVIRAL AGENTS: REVERSE TRANSCRIPTASE INHIBITORS**<br>**Note: For this protocol, since co-infections with active hepatitis B and HIV are excluded, antiviral treatments are not allowed.**		
efavirenz*	↓ telaprevir 1.125 mg q8h<br>AUC 0.82 (0.73-0.92)<br>C_{max} 0.86 (0.76-0.97)<br>C_{min} 0.75 (0.66-0.86)<br>↓ efavirenz (+ TVR 1.125 mg q8h)<br>AUC 0.82 (0.74-0.90)<br>C_{max} 0.76 (0.68-0.85)<br>C_{min} 0.90 (0.81-1.01)	In a drug interaction study in healthy volunteers where telaprevir (at a dose of 1.125 mg q8h) was co-administered with efavirenz, the steady-state efavirenz exposure was reduced by 18%. The steady-state telaprevir exposure was reduced by 18% relative to telaprevir administered 750 mg q8h.
tenofovir disoproxil fumarate*	↔ telaprevir<br>AUC 1.00 (0.94-1.07)<br>C_{max} 1.01 (0.96-1.05)<br>C_{min} 1.03 (0.93-1.14)<br>↑ tenofovir<br>AUC 1.30 (1.22-1.39)<br>C_{max} 1.30 (1.16-1.45)<br>C_{min} 1.41 (1.29-1.54)	In a drug interaction study in healthy volunteers co-administration of telaprevir and tenofovir led to an increase in tenofovir exposure by about 30%. Increased clinical and laboratory monitoring are warranted.
**HMG-CoA REDUCTASE INHIBITORS**		
atorvastatin*	↑ atorvastatin<br>AUC 7.88 (6.82-9.07)<br>C_{max} 10.6 (8.74-12.85)	Exposure to atorvastatin was increased 8-fold when co-administered with telaprevir.<br><br>Caution should be used and dose reduction of atorvastatin should be considered, along with clinical or laboratory monitoring.
**HORMONAL CONTRACEPTIVES/ESTROGEN**		
ethinylestradiol*<br>norethindrone*	↓ ethinylestradiol<br>AUC 0.72 (0.69-0.75)<br>C_{max} 0.74 (0.68-0.80)<br>C_{min} 0.67 (0.63-0.71)<br>↔ norethindrone<br>AUC 0.89 (0.86-0.93)<br>C_{max} 0.85 (0.81-0.89)<br>C_{min} 0.94 (0.87-1.00)	Exposure to ethinylestradiol was decreased by 28% when co-administered with telaprevir. Alternative methods of non-hormonal contraception should be used when estrogen-based contraceptives are co-administered with telaprevir.<br>Patients using estrogens as hormone replacement therapy should be clinically monitored for signs of estrogen deficiency.
**IMMUNOSUPPRESSANTS**		
cyclosporine*<br>sirolimus<br>tacrolimus*	↑ cyclosporine<br>AUC 4.64 (3.90-5.51)<br>C_{max} 1.32 (1.08-1.60)<br>↑ sirolimus<br>↑ tacrolimus<br>AUC 70.3 (52.9-93.4)<br>C_{max} 9.35 (6.73-13.0)	Plasma concentrations of cyclosporine and tacrolimus are markedly increased when co-administered with telaprevir. Plasma concentration of sirolimus may be increased when co-administered with telaprevir, though this has not been studied. Significant dose reductions and prolongation of the dosing interval of the immunosuppressant to achieve the desired blood levels should be anticipated. Close monitoring of the immunosuppressant blood levels, and frequent assessments of renal function and immunosuppressant related side effects are recommended when co-administered with telaprevir. Tacrolimus may prolong the QT interval. The use of telaprevir in organ transplant candidates or patients is not recommended.

(Continued)

Attachment 2: (Continued)

Interactions and Dose Recommendations With Other Medicinal Products

INTERACTIONS AND DOSE RECOMMENDATIONS WITH OTHER MEDICINAL PRODUCTS

**Medicinal products by therapeutic areas**	**Effect on concentration of telaprevir or concomitant medicinal product**	**Clinical Comment**
**INHALED BETA AGONIST**		
salmeterol	↑ salmeterol	Concentrations of salmeterol may be increased when co-administered with telaprevir. Concurrent administration of salmeterol and telaprevir is not recommended. The combination may result in increased risk of cardiovascular adverse events associated with salmeterol, including QT prolongation, palpitations, and sinus tachycardia.
**NARCOTIC ANALGESIC**		
Methadone*	↓ R-methadone<br>AUC 0.71 (0.66-0.76)<br>C_{max} 0.71 (0.66-0.76)<br>C_{min} 0.69 (0.64-0.75)	Concentrations of methadone were reduced by 29% when co-administered with telaprevir. No adjustment of methadone dose is required when initiating co-administration of telaprevir. However, clinical monitoring is recommended as the dose of methadone during maintenance therapy may need to be adjusted in some patients.<br>QT interval prolongation and Torsade de Pointes have been reported with methadone.
**PDE-5 INHIBITORS**		
sildenafil<br>tadalafil<br>vardenafil	↑ PDE-5 inhibitors	Concentrations of PDE-5 inhibitors may be increased when co-administered with telaprevir. For the treatment of erectile dysfunction, sildenafil at a single dose not exceeding 25 mg in 48 hours, vardenafil at a single dose not exceeding 2.5 mg dose in 72 hours, or tadalafil at a single dose not exceeding 10 mg dose in 72 hours can be used with increased monitoring for PDE-5 inhibitor associated adverse events.<br>QT interval prolongation has been reported with vardenafil. Caution is warranted and clinical monitoring is recommended.<br>Co-administration of sildenafil and telaprevir in the treatment of pulmonary arterial hypertension is contraindicated.<br>Co-administration of tadalafil and telaprevir in the treatment of pulmonary arterial hypertension is not recommended.
**PROTON PUMP INHIBITORS**		
esomeprazole*	↔ telaprevir<br>AUC 0.98 (0.91-1.05)<br>C_{max} 0.95 (0.86-1.06)	Since there was no effect of esomeprazole on the plasma concentrations of telaprevir, proton pump inhibitors can be used without dose modification.

Pediatric population

Interaction studies have only been performed in adults.

Attachment 3:
DAIDS Table

DIVISION OF AIDS TABLE FOR GRADING THE SEVERITY OF ADULT AND PEDIATRIC ADVERSE EVENTS. VERSION 1.0, DECEMBER, 2004; CLARIFICATION AUGUST 2009

The Division of AIDS Table for Grading the Severity of Adult and Pediatric Adverse Events ("DAIDS grading table") is a descriptive terminology to be utilized for adverse event reporting in this study. A grading (severity) scale is provided for each adverse event term.

This clarification of the DAIDS Table for Grading the Severity of Adult and Pediatric Adverse Events provides additional explanation of the DAIDS Adverse Event Grading Table and clarifies some of the parameters.

I. Instructions and Clarifications

Grading Adult and Pediatric Adverse Events
The DAIDS Adverse Event Grading Table includes parameters for grading both adult and pediatric adverse events. When a single set of parameters is not appropriate for grading specific types of adverse events for both adult and pediatric populations, separate sets of parameters for adult and/or pediatric populations (with specified respective age ranges) are given in the table. If there is no distinction in the table between adult and pediatric values for a type of adverse event, then the single set of parameters listed is to be used for grading the severity of both adult and pediatric events of that type.

> Note: In the classification of adverse events, the term '**severe**' is not the same as '**serious**'. Severity is an indication of the intensity of a specific event (as in mild, moderate, or severe chest pain). The term 'serious' relates to a participant/event outcome or action criteria, usually associated with events that pose a threat to a participant's life or functioning.

Estimating Severity Grade for Parameters Not Indentified in the Table
In order to grade a clinical adverse event that is not identified in the DAIDS Adverse Event Grading Table, use the category 'Estimating Severity Grade'.

Determining Severity Grade for Parameters 'Between Grades'
If the severity of an adverse event could fall under either one of 2 grades (eg, the severity of an adverse event could be either grade 2 or grade 3), select the higher of the 2 grades for the adverse event. If a laboratory value that is graded as a multiple of the upper limit of the normal range (ULN) or lower limit of the normal range (LLN) falls between 2 grades, select the higher of the 2 grades for the adverse event. For example, Grade 1 is 2.5 x ULN and Grade 2 is 2.6 x ULN for a parameter. If the laboratory value is 2.53 x ULN (which is between the 2 grades), the severity of this adverse event would be Grade 2, the higher of the 2 grades.

Values Below Grade 1
Any laboratory value that is between either the LLN or ULN and Grade 1 should not be graded.

Determining Severity Grade when Local Laboratory Normal Values Overlap with Grade 1 Ranges
In these situations, the severity grading is based on the ranges in the DAIDS Adverse Event Grading Table, even when there is a reference to the local laboratory LLN.

> For example, *Phosphate, Serum, Low, Adult and Pediatric >14 years Grade 1 range is 2.50 mg/dL -<LLN*. A particular laboratory normal range for Phosphate is 2.1 – 3.8 mg/dL.
>
> A participant's actual laboratory value is 2.5. In this case, the value of 2.5 exceeds the LLN for the local laboratory, but will be graded as Grade 1 per DAIDS Adverse Event Grading Table.

Attachment 3: (Continued)
DAIDS Table

II. Definitions of Terms Used in the Table:

Basic self-care functions	Adult: activities such as bathing, dressing, toileting, transfer/movement, continence, and feeding.<br>Young children: activities that are age and culturally appropriate (eg, feeding self with culturally appropriate eating implement).
LLN	Lower limit of normal
Medical intervention	Use of pharmacologic or biologic agent(s) for treatment of an adverse event.
NA	Not Applicable
Operative intervention	Surgical OR other invasive mechanical procedures.
ULN	Upper limit of normal
Usual social & functional activities	Adult: adaptive tasks and desirable activities, such as going to work, shopping, cooking, use of transportation, pursuing a hobby, etc.<br>Young Children: activities that are age and culturally appropriate (eg, social interactions, play activities, learning tasks, etc.).

Attachment 3: (Continued)
DAIDS Table

PARAMETER	GRADE 1 MILD	GRADE 2 MODERATE	GRADE 3 SEVERE	GRADE 4 POTENTIALLY LIFE-THREATENING
**ESTIMATING SEVERITY GRADE**				
Clinical adverse event NOT identified elsewhere in this DAIDS grading table	Symptoms causing no or minimal interference with usual social & functional activities	Symptoms causing greater than minimal interference with usual social & functional activities	Symptoms causing inability to perform usual social & functional activities	Symptoms causing inability to perform basic self-care functions OR Medical or operative intervention indicated to prevent permanent impairment, persistent disability, or death
**SYSTEMIC**				
Acute systemic allergic reaction	Localized urticaria (wheals) with no medical intervention indicated	Localized urticaria with medical intervention indicated OR Mild angioedema with no medical intervention indicated	Generalized urticaria OR Angioedema with medical intervention indicated OR Symptomatic mild bronchospasm	Acute anaphylaxis OR Life-threatening bronchospasm OR laryngeal edema
Chills	Symptoms causing no or minimal interference with usual social & functional activities	Symptoms causing greater than minimal interference with usual social & functional activities	Symptoms causing inability to perform usual social & functional activities	NA
Fatigue<br>Malaise	Symptoms causing no or minimal interference with usual social & functional activities	Symptoms causing greater than minimal interference with usual social & functional activities	Symptoms causing inability to perform usual social & functional activities	Incapacitating fatigue/ malaise symptoms causing inability to perform basic self-care functions
Fever (nonaxillary)	37.7°C – 38.6°C	38.7°C – 39.3°C	39.4°C – 40.5°C	>40.5°C
Pain (indicate body site)<br>DO NOT use for pain due to injection<br>(See Injection site reactions: Injection site pain)<br>See also Headache, Arthralgia, and Myalgia	Pain causing no or minimal interference with usual social & functional activities	Pain causing greater than minimal interference with usual social & functional activities	Pain causing inability to perform usual social & functional activities	Disabling pain causing inability to perform basic self-care functions OR Hospitalization (other than emergency room visit) indicated
Unintentional weight loss	NA	5% – 9% loss in body weight from baseline	10% – 19% loss in body weight from baseline	≥20% loss in body weight from baseline OR Aggressive intervention indicated [eg, tube feeding or total parenteral nutrition (TPN)]

Basic Self-care Functions – Adult: Activities such as bathing, dressing, toileting, transfer/movement, continence, and feeding.

Basic Self-care Functions – Young Children: Activities that are age and culturally appropriate (eg, feeding self with culturally appropriate eating implement).

Usual Social & Functional Activities – Adult: Adaptive tasks and desirable activities, such as going to work, shopping, cooking, use of transportation, pursuing a hobby, etc.

Usual Social & Functional Activities – Young Children: Activities that are age and culturally appropriate (eg, social interactions, play activities, learning tasks, etc.).

Attachment 3: (Continued)

DAIDS Table

PARAMETER		GRADE 1 MILD	GRADE 2 MODERATE	GRADE 3 SEVERE	GRADE 4 POTENTIALLY LIFE-THREATENING
**INFECTION**					
Infection (any other than HIV infection)		Localized, no systemic antimicrobial treatment indicated AND Symptoms causing no or minimal interference with usual social & functional activities	Systemic antimicrobial treatment indicated OR Symptoms causing greater than minimal interference with usual social & functional activities	Systemic antimicrobial treatment indicated AND Symptoms causing inability to perform usual social & functional activities OR Operative intervention (other than simple incision and drainage) indicated	Life-threatening consequences (eg, septic shock)
**INJECTION SITE REACTIONS**					
Injection site pain (pain without touching) Or Tenderness (pain when area is touched)		Pain/tenderness causing no or minimal limitation of use of limb	Pain/tenderness limiting use of limb OR Pain/tenderness causing greater than minimal interference with usual social & functional activities	Pain/tenderness causing inability to perform usual social & functional activities	Pain/tenderness causing inability to perform basic self-care function OR Hospitalization (other than emergency room visit) indicated for management of pain/tenderness
Injection site reaction (localized)					
	**Adult >15 years**	Erythema OR Induration of 5x5 cm – 9x9 cm (or 25 cm² – 81 cm²)	Erythema OR Induration OR Edema >9 cm any diameter (or >81 cm²)	Ulceration OR Secondary infection OR Phlebitis OR Sterile abscess OR Drainage	Necrosis (involving dermis and deeper tissue)
	**Pediatric ≤15 years**	Erythema OR Induration OR Edema present but ≤2.5 cm diameter	Erythema OR Induration OR Edema >2.5 cm diameter but <50% surface area of the extremity seg,ment (eg, upper arm/thigh)	Erythema OR Induration OR Edema involving ≥50% surface area of the extremity seg,ment (eg, upper arm/thigh) OR Ulceration OR Secondary infection OR Phlebitis OR Sterile abscess OR Drainage	Necrosis (involving dermis and deeper tissue)
Pruritis associated with injection See also Skin: Pruritis (itching - no skin lesions)		Itching localized to injection site AND Relieved spontaneously or with <48 hours treatment	Itching beyond the injection site but not generalized OR Itching localized to injection site requiring ≥48 hours treatment	Generalized itching causing inability to perform usual social & functional activities	NA

Basic Self-care Functions – Adult: Activities such as bathing, dressing, toileting, transfer/movement, continence, and feeding.

Basic Self-care Functions – Young Children: Activities that are age and culturally appropriate (eg, feeding self with culturally appropriate eating implement).

Usual Social & Functional Activities – Adult: Adaptive tasks and desirable activities, such as going to work, shopping, cooking, use of transportation, pursuing a hobby, etc.

Usual Social & Functional Activities – Young Children: Activities that are age and culturally appropriate (eg, social interactions, play activities, learning tasks, etc.).

Attachment 3: (Continued)

DAIDS Table

PARAMETER	GRADE 1 MILD	GRADE 2 MODERATE	GRADE 3 SEVERE	GRADE 4 POTENTIALLY LIFE-THREATENING
**SKIN – DERMATOLOGICAL**				
Alopecia	Thinning detectable by study participant (or by careg,iver for young children and disabled adults)	Thinning or patchy hair loss detectable by health care provider	Complete hair loss	NA
Cutaneous reaction/rash	Localized macular rash	Diffuse macular, maculopapular, or morbilliform rash OR Target lesions	Diffuse macular, maculopapular, or morbilliform rash with vesicles or limited number of bullae OR Cutaneous reaction/rash with superficial ulcerations of mucous membrane limited to 1 site[a] OR Cutaneous reaction/rash with at least 1 of the following[a]: elevation of AST and/or ALT >2 x baseline but at least 5 x ULN[a]; fever (>38°C or 100°F)[a]; eosinophils >1000/mm^{3}[a]; serum sickness-like reaction[a]	Extensive or generalized bullous lesions OR Stevens-Johnson syndrome (SJS) OR Ulceration of mucous membrane involving 2 or more distinct mucosal sites OR Toxic epidermal necrolysis (TEN)
Hyperpigmentation	Slight or localized	Marked or generalized	NA	NA
Hypopigmentation	Slight or localized	Marked or generalized	NA	NA
Pruritis (itching – no skin lesions) (See also Injection site reactions: Pruritis associated with injection)	Itching causing no or minimal interference with usual social & functional activities	Itching causing greater than minimal interference with usual social & functional activities	Itching causing inability to perform usual social & functional activities	NA

[a] Revised by the Sponsor

Basic Self-care Functions – Adult: Activities such as bathing, dressing, toileting, transfer/movement, continence, and feeding.

Basic Self-care Functions – Young Children: Activities that are age and culturally appropriate (eg, feeding self with culturally appropriate eating implement).

Usual Social & Functional Activities – Adult: Adaptive tasks and desirable activities, such as going to work, shopping, cooking, use of transportation, pursuing a hobby, etc.

Usual Social & Functional Activities – Young Children: Activities that are age and culturally appropriate (eg, social interactions, play activities, learning tasks, etc.).

Attachment 3: (Continued)

DAIDS Table

PARAMETER	GRADE 1 MILD	GRADE 2 MODERATE	GRADE 3 SEVERE	GRADE 4 POTENTIALLY LIFE-THREATENING
**CARDIOVASCULAR**				
Cardiac arrhythmia (general) (by ECG or physical exam)	Asymptomatic AND No intervention indicated	Asymptomatic AND Nonurgent medical intervention indicated	Symptomatic, non-life threatening AND Nonurgent medical intervention indicated	Life-threatening arrhythmia OR Urgent intervention indicated
Cardiac ischemia/ infarction	NA	NA	Symptomatic ischemia (stable angina) OR Testing consistent with ischemia	Unstable angina OR Acute myocardial Infarction
Hemorrhage (significant acute blood loss)	NA	Symptomatic AND No transfusion indicated	Symptomatic AND Transfusion of ≤2 units packed RBCs (for children ≤10 cc/kg) indicated	Life-threatening hypotension OR Transfusion of >2 units packed RBCs (for children >10 cc/kg) indicated
Hypertension[a]				
**Adult >17 years** (with repeat testing at same visit)	>140 to <160 mmHg systolic OR >90 to <100 mmHg diastolic	≥160 to <180 mmHg systolic OR ≥100 to <110 mmHg diastolic	≥180 mmHg systolic OR ≥110 mmHg diastolic	Life-threatening consequences (eg, malignant hypertension) OR Hospitalization indicated (other than emergency room visit)
**Pediatric ≤17 years** (with repeat testing at same visit)	NA	91st – 94th percentile adjusted for age, height, and gender (systolic and/or diastolic)	≥95th percentile adjusted for age, height, and gender (systolic and/or diastolic)	Life-threatening consequences (eg, malignant hypertension) OR Hospitalization indicated (other than emergency room visit)
Hypotension	NA	Symptomatic, corrected with oral fluid replacement	Symptomatic, i.v. fluids indicated	Shock requiring use of vasopressors or mechanical assistance to maintain blood pressure
Pericardial effusion	Asymptomatic, small effusion requiring no intervention	Asymptomatic, moderate or larger effusion requiring no intervention	Effusion with non-life threatening physiologic consequences OR Effusion with nonurgent intervention indicated	Life-threatening consequences (eg, tamponade) OR Urgent intervention indicated

[a] Revised by the Sponsor

Basic Self-care Functions – Adult: Activities such as bathing, dressing, toileting, transfer/movement, continence, and feeding.

Basic Self-care Functions – Young Children: Activities that are age and culturally appropriate (eg, feeding self with culturally appropriate eating implement).

Usual Social & Functional Activities – Adult: Adaptive tasks and desirable activities, such as going to work, shopping, cooking, use of transportation, pursuing a hobby, etc.

Usual Social & Functional Activities – Young Children: Activities that are age and culturally appropriate (eg, social interactions, play activities, learning tasks, etc.).

Attachment 3: (Continued)

DAIDS Table

PARAMETER		GRADE 1 MILD	GRADE 2 MODERATE	GRADE 3 SEVERE	GRADE 4 POTENTIALLY LIFE-THREATENING
Prolonged PR interval					
	**Adult >16 years**	PR interval 0.21 – 0.25 s	PR interval >0.25 s	Type II 2nd degree AV block OR Ventricular pause >3.0 s	Complete AV block
	**Pediatric ≤16 years**	1st degree AV block (PR > normal for age and rate)	Type I 2nd degree AV block	Type II 2nd degree AV block	Complete AV block
Prolonged QTc					
	**Adult >16 years**	Asymptomatic, QTc interval 0.45 – 0.47 s OR Increase in interval <0.03 s above baseline	Asymptomatic, QTc interval 0.48 – 0.49 s OR Increase in interval 0.03 – 0.05 s above baseline	Asymptomatic, QTc interval ≥0.50 s OR Increase in interval ≥0.06 s above baseline	Life-threatening consequences, eg, Torsade de pointes or other associated serious ventricular dysrhythmia
	**Pediatric ≤16 years**	Asymptomatic, QTc interval 0.450 – 0.464 s	Asymptomatic, QTc interval 0.465 – 0.479 s	Asymptomatic, QTc interval ≥0.480 s	Life-threatening consequences, eg, Torsade de pointes or other associated serious ventricular dysrhythmia
Thrombosis/ embolism		NA	Deep vein thrombosis AND No intervention indicated (eg, anticoagulation, lysis filter, invasive procedure)	Deep vein thrombosis AND Intervention indicated (eg, anticoagulation, lysis filter, invasive procedure)	Embolic event (eg, pulmonary embolism, life-threatening thrombus)
Vasovagal episode (associated with a procedure of any kind)		Present without loss of consciousness	Present with transient loss of consciousness	NA	NA
Ventricular dysfunction (congestive heart failure)		NA	Asymptomatic diagnostic finding AND intervention indicated	New onset with symptoms OR Worsening symptomatic congestive heart failure	Life-threatening congestive heart failure
**GASTROINTESTINAL**					
Anorexia		Loss of appetite without decreased oral intake	Loss of appetite associated with decreased oral intake without significant weight loss	Loss of appetite associated with significant weight loss	Life-threatening consequences OR Aggressive intervention indicated (eg, tube feeding or total parenteral nutrition [TPN])
Please note that, while the grading scale provided for Unintentional Weight Loss may be used as a guideline when grading anorexia, this is not a requirement and should not be used as a substitute for clinical judgment.					

Basic Self-care Functions – Adult: Activities such as bathing, dressing, toileting, transfer/movement, continence, and feeding.

Basic Self-care Functions – Young Children: Activities that are age and culturally appropriate (eg, feeding self with culturally appropriate eating implement).

Usual Social & Functional Activities – Adult: Adaptive tasks and desirable activities, such as going to work, shopping, cooking, use of transportation, pursuing a hobby, etc.

Usual Social & Functional Activities – Young Children: Activities that are age and culturally appropriate (eg, social interactions, play activities, learning tasks, etc.)

Attachment 3: (Continued)

DAIDS Table

PARAMETER	GRADE 1 MILD	GRADE 2 MODERATE	GRADE 3 SEVERE	GRADE 4 POTENTIALLY LIFE-THREATENING
Ascites	Asymptomatic	Symptomatic AND Intervention indicated (eg, diuretics or therapeutic paracentesis)	Symptomatic despite intervention	Life-threatening consequences
Cholecystitis	NA	Symptomatic AND Medical intervention indicated	Radiologic, endoscopic, or operative intervention indicated	Life-threatening consequences (eg, sepsis or perforation)
Constipation	NA	Persistent constipation requiring reg,ular use of dietary modifications, laxatives, or enemas	Obstipation with manual evacuation indicated	Life-threatening consequences (eg, obstruction)
Diarrhea				
**Adult and Pediatric ≥1 year**	Transient or intermittent episodes of unformed stools OR Increase of ≤3 stools over baseline per 24-hour period	Persistent episodes of unformed to watery stools OR Increase of 4 – 6 stools over baseline per 24-hour period	Bloody diarrhea OR Increase of ≥7 stools per 24-hour period OR i.v. fluid replacement indicated	Life-threatening consequences (eg, hypotensive shock)
**Pediatric <1 year**	Liquid stools (more unformed than usual) but usual number of stools	Liquid stools with increased number of stools OR Mild dehydration	Liquid stools with moderate dehydration	Liquid stools resulting in severe dehydration with aggressive rehydration indicated OR Hypotensive shock
Dysphagia-Odynophagia	Symptomatic but able to eat usual diet	Symptoms causing altered dietary intake without medical intervention indicated	Symptoms causing severely altered dietary intake with medical intervention indicated	Life-threatening reduction in oral intake
Mucositis/ stomatitis (clinical exam) Indicate site (eg, larynx, oral) See Genitourinary for Vulvovaginitis See also Dysphagia-Odynophagia and Proctitis	Erythema of the Mucosa	Patchy pseudomembranes or ulcerations	Confluent pseudomembranes or ulcerations OR Mucosal bleeding with minor trauma	Tissue necrosis OR Diffuse spontaneous mucosal bleeding OR Life-threatening consequences (eg, aspiration, choking)

Basic Self-care Functions – Adult: Activities such as bathing, dressing, toileting, transfer/movement, continence, and feeding.

Basic Self-care Functions – Young Children: Activities that are age and culturally appropriate (eg, feeding self with culturally appropriate eating implement).

Usual Social & Functional Activities – Adult: Adaptive tasks and desirable activities, such as going to work, shopping, cooking, use of transportation, pursuing a hobby, etc.

Usual Social & Functional Activities – Young Children: Activities that are age and culturally appropriate (eg, social interactions, play activities, learning tasks, etc.)

Attachment 3: (Continued)
DAIDS Table

PARAMETER	GRADE 1 MILD	GRADE 2 MODERATE	GRADE 3 SEVERE	GRADE 4 POTENTIALLY LIFE-THREATENING
Nausea	Transient (<24 hours) or intermittent nausea with no or minimal interference with oral intake	Persistent nausea resulting in decreased oral intake for 24 – 48 hours	Persistent nausea resulting in minimal oral intake for >48 hours OR Aggressive rehydration indicated (eg, i.v. fluids)	Life-threatening consequences (eg, hypotensive shock)
Pancreatitis	NA	Symptomatic AND Hospitalization not indicated (other than emergency room visit)	Symptomatic AND Hospitalization indicated (other than emergency room visit)	Life-threatening consequences (eg, circulatory failure, hemorrhage, sepsis)
Proctitis (functional-symptomatic) Also see Mucositis/ stomatitis for clinical exam	Rectal discomfort AND No intervention Indicated	Symptoms causing greater than minimal interference with usual social & functional activities OR Medical intervention indicated	Symptoms causing inability to perform usual social & functional activities OR Operative intervention indicated	Life-threatening consequences (eg, perforation)
Vomiting	Transient or intermittent vomiting with no or minimal interference with oral intake	Frequent episodes of vomiting with no or mild dehydration	Persistent vomiting resulting in orthostatic hypotension OR Aggressive rehydration indicated (eg, i.v. fluids)	Life-threatening consequences (eg, hypotensive shock)
**NEUROLOGIC**				
Alteration in personality-behavior or in mood (eg, agitation, anxiety, depression, mania, psychosis)	Alteration causing no or minimal interference with usual social & functional activities	Alteration causing greater than minimal interference with usual social & functional activities	Alteration causing inability to perform usual social & functional activities	Behavior potentially harmful to self or others (eg, suicidal and homicidal ideation or attempt, acute psychosis) OR Causing inability to perform basic self-care functions
Altered Mental Status For Dementia, see Cognitive and behavioral/ attentional disturbance (including dementia and attention deficit disorder)	Changes causing no or minimal interference with usual social & functional activities	Mild lethargy or somnolence causing greater than minimal interference with usual social & functional activities	Confusion, memory impairment, lethargy, or somnolence causing inability to perform usual social & functional activities	Delirium OR obtundation, OR coma

Basic Self-care Functions – Adult: Activities such as bathing, dressing, toileting, transfer/movement, continence, and feeding.

Basic Self-care Functions – Young Children: Activities that are age and culturally appropriate (eg, feeding self with culturally appropriate eating implement).

Usual Social & Functional Activities – Adult: Adaptive tasks and desirable activities, such as going to work, shopping, cooking, use of transportation, pursuing a hobby, etc.

Usual Social & Functional Activities – Young Children: Activities that are age and culturally appropriate (eg, social interactions, play activities, learning tasks, etc.)

Attachment 3: (Continued)

DAIDS Table

PARAMETER	GRADE 1 MILD	GRADE 2 MODERATE	GRADE 3 SEVERE	GRADE 4 POTENTIALLY LIFE-THREATENING
Ataxia	Asymptomatic ataxia detectable on exam OR Minimal ataxia causing no or minimal interference with usual social & functional activities	Symptomatic ataxia causing greater than minimal interference with usual social & functional activities	Symptomatic ataxia causing inability to perform usual social & functional activities	Disabling ataxia causing inability to perform basic self-care functions
Cognitive and behavioral/ attentional disturbance (including dementia and attention deficit disorder)	Disability causing no or minimal interference with usual social & functional activities OR Specialized resources not indicated	Disability causing greater than minimal interference with usual social & functional activities OR Specialized resources on part-time basis indicated	Disability causing inability to perform usual social & functional activities OR Specialized resources on a full-time basis indicated	Disability causing inability to perform basic self-care functions OR Institutionalization Indicated
CNS ischemia (acute)	NA	NA	Transient ischemic Attack	Cerebral vascular accident (CVA, stroke) with neurological deficit
Developmental delay **Pediatric ≤16 years**	Mild developmental delay, either motor or cognitive, as determined by comparison with a developmental screening tool appropriate for the setting	Moderate developmental delay, either motor or cognitive, as determined by comparison with a developmental screening tool appropriate for the setting	Severe developmental delay, either motor or cognitive, as determined by comparison with a developmental screening tool appropriate for the setting	Developmental regression, either motor or cognitive, as determined by comparison with a developmental screening tool appropriate for the setting
Headache	Symptoms causing no or minimal interference with usual social & functional activities	Symptoms causing greater than minimal interference with usual social & functional activities	Symptoms causing inability to perform usual social & functional activities	Symptoms causing inability to perform basic self-care functions OR Hospitalization indicated (other than emergency room visit) OR Headache with significant impairment of alertness or other neurologic function
Insomnia	NA	Difficulty sleeping causing greater than minimal interference with usual social & functional activities	Difficulty sleeping causing inability to perform usual social & functional activities	Disabling insomnia causing inability to perform basic self-care functions

Basic Self-care Functions – Adult: Activities such as bathing, dressing, toileting, transfer/movement, continence, and feeding.

Basic Self-care Functions – Young Children: Activities that are age and culturally appropriate (eg, feeding self with culturally appropriate eating implement).

Usual Social & Functional Activities – Adult: Adaptive tasks and desirable activities, such as going to work, shopping, cooking, use of transportation, pursuing a hobby, etc.

Usual Social & Functional Activities – Young Children: Activities that are age and culturally appropriate (eg, social interactions, play activities, learning tasks, etc.)

Attachment 3: (Continued)

DAIDS Table

PARAMETER	GRADE 1 MILD	GRADE 2 MODERATE	GRADE 3 SEVERE	GRADE 4 POTENTIALLY LIFE-THREATENING
Neuromuscular weakness (including myopathy & neuropathy)	Asymptomatic with decreased strength on exam OR Minimal muscle weakness causing no or minimal interference with usual social & functional activities	Muscle weakness causing greater than minimal interference with usual social & functional activities	Muscle weakness causing inability to perform usual social & functional activities	Disabling muscle weakness causing inability to perform basic self-care functions OR Respiratory muscle weakness impairing ventilation
Neurosensory alteration (including paresthesia and painful neuropathy)	Asymptomatic with sensory alteration on exam or minimal paresthesia causing no or minimal interference with usual social & functional activities	Sensory alteration or paresthesia causing greater than minimal interference with usual social & functional activities	Sensory alteration or paresthesia causing inability to perform usual social & functional activities	Disabling sensory alteration or paresthesia causing inability to perform basic self-care functions
Seizure: (new onset) **Adult ≥18 years** See also Seizure: (known pre-existing seizure disorder)	NA	1 seizure	2 – 4 seizures	Seizures of any kind which are prolonged, repetitive (eg, status epilepticus), or difficult to control (eg, refractory epilepsy)
Seizure: (known pre-existing seizure disorder) **Adult ≥18 years** For worsening of existing epilepsy the grades should be based on an increase from previous level of control to any of these levels.	NA	Increased frequency of pre-existing seizures (nonrepetitive) without change in seizure character OR Infrequent breakthrough seizures while on stable medication in a previously controlled seizure disorder	Change in seizure character from baseline either in duration or quality (eg, severity or focality)	Seizures of any kind which are prolonged, repetitive (eg, status epilepticus), or difficult to control (eg, refractory epilepsy)
Seizure **Pediatric <18 years**	Seizure, generalized onset with or without secondary generalization, lasting <5 minutes with <24 hours postictal state	Seizure, generalized onset with or without secondary generalization, lasting 5 – 20 minutes with <24 hours postictal state	Seizure, generalized onset with or without secondary generalization, lasting >20 minutes	Seizure, generalized onset with or without secondary generalization, requiring intubation and sedation
Syncope (not associated with a procedure)	NA	Present	NA	NA

Basic Self-care Functions – Adult: Activities such as bathing, dressing, toileting, transfer/movement, continence, and feeding.

Basic Self-care Functions – Young Children: Activities that are age and culturally appropriate (eg, feeding self with culturally appropriate eating implement).

Usual Social & Functional Activities – Adult: Adaptive tasks and desirable activities, such as going to work, shopping, cooking, use of transportation, pursuing a hobby, etc.

Usual Social & Functional Activities – Young Children: Activities that are age and culturally appropriate (eg, social interactions, play activities, learning tasks, etc.)

Attachment 3: (Continued)

DAIDS Table

PARAMETER		GRADE 1<br>MILD	GRADE 2<br>MODERATE	GRADE 3<br>SEVERE	GRADE 4<br>POTENTIALLY<br>LIFE-THREATENING
Vertigo		Vertigo causing no or minimal interference with usual social & functional activities	Vertigo causing greater than minimal interference with usual social & functional activities	Vertigo causing inability to perform usual social & functional activities	Disabling vertigo causing inability to perform basic self-care functions
**RESPIRATORY**					
Bronchospasm (acute)		FEV1 or peak flow reduced to 70% – 80%	FEV1 or peak flow 50% – 69%	FEV1 or peak flow 25% – 49%	Cyanosis OR FEV1 or peak flow <25% OR Intubation
Dyspnea or respiratory distress					
	**Adult ≥14 years**	Dyspnea on exertion with no or minimal interference with usual social & functional activities	Dyspnea on exertion causing greater than minimal interference with usual social & functional activities	Dyspnea at rest causing inability to perform usual social & functional activities	Respiratory failure with ventilatory support indicated
	**Pediatric <14 years**	Wheezing OR minimal increase in respiratory rate for age	Nasal flaring OR Intercostal retractions OR Pulse oximetry 90% – 95%	Dyspnea at rest causing inability to perform usual social & functional activities OR Pulse oximetry <90%	Respiratory failure with ventilatory support indicated
**MUSCULOSKELETAL**					
Arthralgia<br>See also Arthritis		Joint pain causing no or minimal interference with usual social & functional activities	Joint pain causing greater than minimal interference with usual social & functional activities	Joint pain causing inability to perform usual social & functional activities	Disabling joint pain causing inability to perform basic self-care functions
Arthritis<br>See also Arthralgia		Stiffness or joint swelling causing no or minimal interference with usual social & functional activities	Stiffness or joint swelling causing greater than minimal interference with usual social & functional activities	Stiffness or joint swelling causing inability to perform usual social & functional activities	Disabling joint stiffness or swelling causing inability to perform basic self-care functions
Bone Mineral Loss					
	**Adult ≥21 years**	BMD t-score -2.5 to -1.0	BMD t-score <-2.5	Pathological fracture (including loss of vertebral height)	Pathologic fracture causing life-threatening consequences
	**Pediatric <21 years**	BMD z-score -2.5 to -1.0	BMD z-score <-2.5	Pathological fracture (including loss of vertebral height)	Pathologic fracture causing life-threatening consequences
Myalgia (noninjection site)		Muscle pain causing no or minimal interference with usual social & functional activities	Muscle pain causing greater than minimal interference with usual social & functional activities	Muscle pain causing inability to perform usual social & functional activities	Disabling muscle pain causing inability to perform basic self-care functions

Basic Self-care Functions – Adult: Activities such as bathing, dressing, toileting, transfer/movement, continence, and feeding.

Basic Self-care Functions – Young Children: Activities that are age and culturally appropriate (eg, feeding self with culturally appropriate eating implement).

Usual Social & Functional Activities – Adult: Adaptive tasks and desirable activities, such as going to work, shopping, cooking, use of transportation, pursuing a hobby, etc.

Usual Social & Functional Activities – Young Children: Activities that are age and culturally appropriate (eg, social interactions, play activities, learning tasks, etc.)

Attachment 3: (Continued)

DAIDS Table

PARAMETER	GRADE 1 MILD	GRADE 2 MODERATE	GRADE 3 SEVERE	GRADE 4 POTENTIALLY LIFE-THREATENING
Osteonecrosis	NA	Asymptomatic with radiographic findings AND No operative intervention indicated	Symptomatic bone pain with radiographic findings OR Operative intervention indicated	Disabling bone pain with radiographic findings causing inability to perform basic self-care functions
**GENITOURINARY**				
Cervicitis (symptoms) (For use in studies evaluating topical study agents) For other cervicitis see Infection: Infection (any other than HIV infection)	Symptoms causing no or minimal interference with usual social & functional activities	Symptoms causing greater than minimal interference with usual social & functional activities	Symptoms causing inability to perform usual social & functional activities	Symptoms causing inability to perform basic self-care functions
Cervicitis (clinical exam) (For use in studies evaluating topical study agents) For other cervicitis, see Infection: Infection (any other than HIV infection)	Minimal cervical abnormalities on examination (erythema, mucopurulent discharge, or friability) OR Epithelial disruption <25% of total surface	Moderate cervical abnormalities on examination (erythema, mucopurulent discharge, or friability) OR Epithelial disruption of 25% – 49% total surface	Severe cervical abnormalities on examination (erythema, mucopurulent discharge, or friability) OR Epithelial disruption 50% – 75% total surface	Epithelial disruption >75% total surface
Intermenstrual bleeding (IMB)	Spotting observed by participant OR Minimal blood observed during clinical or colposcopic examination	Inter-menstrual bleeding not greater in duration or amount than usual menstrual cycle	Inter-menstrual bleeding greater in duration or amount than usual menstrual cycle	Hemorrhage with lifethreatening hypotension OR Operative intervention indicated
Urinary tract obstruction (eg, stone)	NA	Signs or symptoms of urinary tract obstruction without hydronephrosis or renal dysfunction	Signs or symptoms of urinary tract obstruction with hydronephrosis or renal dysfunction	Obstruction causing lifethreatening Consequences

Basic Self-care Functions – Adult: Activities such as bathing, dressing, toileting, transfer/movement, continence, and feeding.

Basic Self-care Functions – Young Children: Activities that are age and culturally appropriate (eg, feeding self with culturally appropriate eating implement).

Usual Social & Functional Activities – Adult: Adaptive tasks and desirable activities, such as going to work, shopping, cooking, use of transportation, pursuing a hobby, etc.

Usual Social & Functional Activities – Young Children: Activities that are age and culturally appropriate (eg, social interactions, play activities, learning tasks, etc.)

Attachment 3: (Continued)

DAIDS Table

PARAMETER	GRADE 1 MILD	GRADE 2 MODERATE	GRADE 3 SEVERE	GRADE 4 POTENTIALLY LIFE-THREATENING
Vulvovaginitis (symptoms) (Use in studies evaluating topical study agents) For other vulvovaginitis see Infection: Infection (any other than HIV infection)	Symptoms causing no or minimal interference with usual social & functional activities	Symptoms causing greater than minimal interference with usual social & functional activities	Symptoms causing inability to perform usual social & functional activities	Symptoms causing inability to perform basic self-care functions
Vulvovaginitis (clinical exam) (Use in studies evaluating topical study agents) For other vulvovaginitis see Infection: Infection (any other than HIV infection)	Minimal vaginal abnormalities on examination OR Epithelial disruption <25% of total surface	Moderate vaginal abnormalities on examination OR Epithelial disruption of 25% – 49% total surface	Severe vaginal abnormalities on examination OR Epithelial disruption 50% – 75% total surface	Vaginal perforation OR Epithelial disruption >75% total surface
**OCULAR/VISUAL**				
Uveitis	Asymptomatic but detectable on exam	Symptomatic anterior uveitis OR Medical intervention indicated	Posterior or pan-uveitis OR Operative intervention indicated	Disabling visual loss in affected eye(s)
Visual changes (from baseline)	Visual changes causing no or minimal interference with usual social & functional activities	Visual changes causing greater than minimal interference with usual social & functional activities	Visual changes causing inability to perform usual social & functional activities	Disabling visual loss in affected eye(s)
**ENDOCRINE/METABOLIC**				
Abnormal fat accumulation (eg, back of neck, breasts, abdomen)	Detectable by study participant (or by caregiver for young children and disabled adults)	Detectable on physical exam by health care provider	Disfiguring OR Obvious changes on casual visual inspection	NA
Diabetes mellitus	NA	New onset without need to initiate medication OR Modification of current medications to reg,ain glucose control	New onset with initiation of medication indicated OR Diabetes uncontrolled despite treatment modification	Life-threatening consequences (eg, ketoacidosis, hyperosmolar nonketotic coma)

Basic Self-care Functions – Adult: Activities such as bathing, dressing, toileting, transfer/movement, continence, and feeding.

Basic Self-care Functions – Young Children: Activities that are age and culturally appropriate (eg, feeding self with culturally appropriate eating implement).

Usual Social & Functional Activities – Adult: Adaptive tasks and desirable activities, such as going to work, shopping, cooking, use of transportation, pursuing a hobby, etc.

Usual Social & Functional Activities – Young Children: Activities that are age and culturally appropriate (eg, social interactions, play activities, learning tasks, etc.)

Attachment 3: (Continued)
DAIDS Table

PARAMETER	GRADE 1 MILD	GRADE 2 MODERATE	GRADE 3 SEVERE	GRADE 4 POTENTIALLY LIFE-THREATENING
Gynecomastia	Detectable by study participant or careg,iver (for young children and disabled adults)	Detectable on physical exam by health care provider	Disfiguring OR Obvious on casual visual inspection	NA
Hyperthyroidism	Asymptomatic	Symptomatic causing greater than minimal interference with usual social & functional activities OR Thyroid suppression therapy indicated	Symptoms causing inability to perform usual social & functional activities OR Uncontrolled despite treatment modification	Life-threatening consequences (eg, thyroid storm)
Hypothyroidism	Asymptomatic	Symptomatic causing greater than minimal interference with usual social & functional activities OR Thyroid replacement therapy indicated	Symptoms causing inability to perform usual social & functional activities OR Uncontrolled despite treatment modification	Life-threatening consequences (eg, myxedema coma)
Lipoatrophy (eg, fat loss from the face, extremities, buttocks)	Detectable by study participant (or by caregiver for young children and disabled adults)	Detectable on physical exam by health care provider	Disfiguring OR Obvious on casual visual inspection	NA

Basic Self-care Functions – Adult: Activities such as bathing, dressing, toileting, transfer/movement, continence, and feeding.

Basic Self-care Functions – Young Children: Activities that are age and culturally appropriate (eg, feeding self with culturally appropriate eating implement).

Usual Social & Functional Activities – Adult: Adaptive tasks and desirable activities, such as going to work, shopping, cooking, use of transportation, pursuing a hobby, etc.

Usual Social & Functional Activities – Young Children: Activities that are age and culturally appropriate (eg, social interactions, play activities, learning tasks, etc.)

Attachment 3: (Continued)
DAIDS Table

LABORATORY					
**PARAMETER**		**GRADE 1 MILD**	**GRADE 2 MODERATE**	**GRADE 3 SEVERE**	**GRADE 4 POTENTIALLY LIFE-THREATENING**
**HEMATOLOGY** ***Standard International Units are listed in italics***					
Absolute CD4+ count **Adult and Pediatric >13 years** (HIV negative only)		300 – 400/mm³ *300 – 400/µL*	200 – 299/mm³ *200 – 299/µL*	100 – 199/mm³ *100 – 199/µL*	<100/mm³ *<100/µL*
Absolute lymphocyte count **Adult and Pediatric >13 years** (HIV negative only)		600 – 650/mm³ *0.600×10^9 – 0.650×10^9/L*	500 – 599/mm³ *0.500×10^9 – 0.599×10^9/L*	350 – 499/mm³ *0.350×10^9 – 0.499×10^9/L*	<350/mm³ *<0.350×10^9/L*
Values in children ≤13 years are not given for the two parameters above because the absolute counts are variable.					
Absolute neutrophil count (ANC)					
	**Adult and Pediatric >7 days**	1,000 – 1,300/mm³ *1.000×10^9 – 1.300×10^9/L*	750 – 999/mm³ *0.750×10^9 – 0.999×10^9 /L*	500 – 749/mm³ *0.500×10^9 – 0.749×10^9/L*	<500/mm³ *<0.500×10^9/L*
	**Infant[a,b] 2 – ≤ 7 days**	1,250 – 1,500/mm³ *1.250×10^9 – 1.500×10^9/L*	1,000 – 1,249/mm³ *1.000×10^9 – 1.249×10^9/L*	750 – 999/mm³ *0.750×10^9 – 0.999×10^9/L*	<750/mm³ *<0.750×10^9/L*
	**Infant[a,b] ≤ 1 day**	4,000 – 5,000/mm³ *4.000×10^9 – 5.000×10^9/L*	3,000 – 3,999/mm³ *3.000×10^9 – $3.999 x10^9$/L*	1,500 – 2,999/mm³ *1.500×10^9 – 2.999×10^9/L*	<1,500/mm³ *<1.500×10^9/L*
Fibrinogen, decreased[c]		100 – 200 mg/dL *1.00 – 2.00 g/L* OR ≥0.75 to <1.00 x LLN	75 – 99 mg/dL *0.75 – 0.99 g/L* OR ≥0.50 to <0.75 x LLN	50 – 74 mg/dL *0.50 – 0.74 g/L* OR ≥0.25 to <0.50 x LLN	<50 mg/dL *<0.50 g/L* OR <0.25 x LLN OR Associated with gross bleeding
Hemoglobin (Hgb)[d]					
	**Adult and Pediatric ≥57 days** (HIV positive only)	8.5 – 10.0 g/dL *5.2 – 6.1 mmol/L*	7.5 – 8.4 g/dL *4.6 – 5.1 mmol/L*	6.5 – 7.4 g/dL *3.9 – 4.5 mmol/L*	<6.5 g/dL *<3.9 mmol/L*
	**Adult and Pediatric ≥57 days** (HIV negative only)	10.0 – 10.9 g/dL *6.1 – 6.6 mmol/L* OR Any decrease 2.5 – 3.4 g/dL *1.5 – 2.0 mmol/L*	9.0 – 9.9 g/dL *5.5 – 6.0 mmol/L* OR Any decrease 3.5 – 4.4 g/dL *2.1 – 2.6 mmol/L*	7.0 – 8.9 g/dL *4.2 – 5.4 mmol/L* OR Any decrease ≥ 4.5 g/dL *≥ 2.7 mmol/L*	<7.0 g/dL *<4.2 mmol/L*
	The decrease is a decrease from baseline.				
	**Infant[a,b] 36 – 56 days** (HIV positive or negative)	8.5 – 9.4 g/dL *5.2 – 5.7 mmol/L*	7.0 – 8.4 g/dL *4.2 – 5.1 mmol/L*	6.0 – 6.9 g/dL *3.6 – 4.1 mmol/L*	<6.0 g/dL *<3.6 mmol/L*
	**Infant[a,b] 22 – 35 days** (HIV positive or negative)	9.5 – 10.5 g/dL *5.8 – 6.4 mmol/L*	8.0 – 9.4 g/dL *4.8 – 5.7 mmol/L*	7.0 – 7.9 g/dL *4.2 – 4.7 mmol/L*	<7.00 g/dL *<4.2 mmol/L*

[a] Values are for term infants; preterm infants should be assessed using local normal ranges.
[b] Use age and sex appropriate values (eg, bilirubin).
[c] Revised by the Sponsor.
[d] Revised by the Sponsor; monomer conversion factor used for conversion from g/dL to mmol/L

Attachment 3: (Continued)

DAIDS Table

**LABORATORY**				
**PARAMETER**	**GRADE 1 MILD**	**GRADE 2 MODERATE**	**GRADE 3 SEVERE**	**GRADE 4 POTENTIALLY LIFE-THREATENING**
**Infant[a,b]** **1 – 21 days** (HIV positive or negative)	12.0 – 13.0 g/dL<br>*7.3 – 7.9 mmol/L*	10.0 – 11.9 g/dL<br>*6.1 – 7.2 mmol/L*	9.0 – 9.9 g/dL<br>*5.5 – 6.0 mmol/L*	<9.0 g/dL<br>*<5.5 mmol/L*
International normalized ratio of prothrombin time (INR)[c]	≥1.1 to ≤1.5 x ULN	>1.5 to ≤2.0 x ULN	>2.0 to ≤3.0 x ULN	>3.0 x ULN
Methemoglobin	5.0% – 10.0%	10.1% – 15.0%	15.1% – 20.0%	>20.0%
Prothrombin time (PT)[c,d]	≥1.1 to ≤1.25 x ULN	>1.25 to ≤1.50 x ULN	>1.50 to ≤3.00 x ULN	>3.00 x ULN
Partial thromboplastin time (PTT)[c]	≥1.1 to ≤1.66 x ULN	>1.66 to ≤2.33 x ULN	>2.33 to ≤3.00 x ULN	>3.00 x ULN
Platelets, decreased	100,000 – 124,999/mm^3<br>*100.000 x 10^9 – 124.999 x 10^9/L*	50,000 – 99,999/mm^3<br>*50.000 x 10^9– 99.999 x 10^9/L*	25,000 – 49,999/mm^3<br>*25.000 x 10^9– 49.999 x 10^9/L*	<25,000/mm^3<br>*<25.000 x 10^9/L*
WBC, decreased	2,000 – 2,500/mm^3<br>*2.000 x 10^9 – 2.500 x 10^9/L*	1,500 – 1,999/mm^3<br>*1.500 x 10^9– 1.999 x 10^9/L*	1,000 – 1,499/mm^3<br>*1.000 x 10^9– 1.499 x 10^9/L*	<1,000/mm^3<br>*<1.000 x 10^9/L*
**CHEMISTRIES** ***Standard International Units are listed in italics***				
Acidosis	NA	pH <normal, but ≥7.3	pH <7.3 without life-threatening consequences	pH <7.3 with life-threatening consequences
Albumin, serum, low	3.0 g/dL – <LLN<br>*30 g/L – <LLN*	2.0 – 2.9 g/dL<br>*20 – 29 g/L*	<2.0 g/dL<br>*<20 g/L*	NA
Alkaline phosphatase[c]	≥1.25 to ≤2.5 x ULN[c]	>2.5 to ≤5.0 x ULN[c]	>5.0 to ≤10.0 x ULN[c]	>10.0 x ULN[c]
Alkalosis	NA	pH >normal, but ≤7.5	pH >7.5 without life-threatening consequences	pH >7.5 with life-threatening consequences
ALT (SGPT)[c]	≥1.25 to ≤2.5 x ULN	>2.5 to ≤5.0 x ULN	>5.0 to ≤10.0 x ULN	>10.0 x ULN
AST (SGOT)[c]	≥1.25 to ≤2.5 x ULN	>2.5 to ≤5.0 x ULN	>5.0 to ≤10.0 x ULN	>10.0 x ULN
Bicarbonate, serum, low[e]	16.0 mEq/L – <LLN<br>*16.0 mmol/L – <LLN*	11.0 – 15.9 mEq/L<br>*11.0 – 15.9 mmol/L*	8.0 – 10.9 mEq/L<br>*8.0 – 10.9 mmol/L*	<8.0 mEq/L<br>*<8.0 mmol/L*

[a] Values are for term infants; preterm infants should be assessed using local normal ranges.
[b] Use age- and sex-appropriate values (eg, bilirubin).
[c] Revised by the Sponsor.
[d] If the local laboratory is reporting PT as percentage, only INR value will be considered for reporting PT related abnormalities and adverse events.
[e] Some laboratories will report this value as Bicarbonate (HCO_3) and others as Total Carbon Dioxide (CO_2). These are the same tests; values should be graded according to the ranges for Bicarbonate as listed above.

Attachment 3: (Continued)

DAIDS Table

LABORATORY					
**PARAMETER**		**GRADE 1 MILD**	**GRADE 2 MODERATE**	**GRADE 3 SEVERE**	**GRADE 4 POTENTIALLY LIFE-THREATENING**
Bilirubin (Total)[a]					
	**Adult and Pediatric >14 days**	≥1.1 to ≤1.5 x ULN	>1.5 to ≤2.5 x ULN	>2.5 to ≤5.0 x ULN	>5.0 x ULN
	**Infant[b,c] ≤14 days** (non-hemolytic)	NA	20.0 – 25.0 mg/dL *342 – 428 µmol/L*	25.1 – 30.0 mg/dL *429 – 513 µmol/L*	>30.0 mg/dL *>513.0 µmol/L*
	**Infant[b,c] ≤14 days** (hemolytic)	NA	NA	20.0 – 25.0 mg/dL *342 – 428 µmol/L*	>25.0 mg/dL *>428 µmol/L*
Calcium, serum, high (corrected for albumin)					
	**Adult and Pediatric ≥ 7 days**	10.6 – 11.5 mg/dL *2.65 – 2.88 mmol/L*	11.6 – 12.5 mg/dL *2.89 – 3.13 mmol/L*	12.6 – 13.5 mg/dL *3.14 – 3.38 mmol/L*	>13.5 mg/dL *>3.38 mmol/L*
	**Infant[b,c] <7 days**	11.5 – 12.4 mg/dL *2.88 – 3.10 mmol/L*	12.5 – 12.9 mg/dL *3.11 – 3.23 mmol/L*	13.0 – 13.5 mg/dL *3.24 – 3.38 mmol/L*	>13.5 mg/dL *>3.38 mmol/L*
Calcium, serum, low (corrected for albumin)					
	**Adult and Pediatric ≥ 7 days**	7.8 – 8.4 mg/dL *1.95 – 2.10 mmol/L*	7.0 – 7.7 mg/dL *1.75 – 1.94 mmol/L*	6.1 – 6.9 mg/dL *1.53 – 1.74 mmol/L*	<6.1 mg/dL *<1.53 mmol/L*
	**Infant[a,b] <7 days**	6.5 – 7.5 mg/dL *1.63 – 1.88 mmol/L*	6.0 – 6.4 mg/dL *1.50 – 1.62 mmol/L*	5.50 – 5.90 mg/dL *1.38 – 1.49 mmol/L*	<5.50 mg/dL *<1.38 mmol/L*
Do not adjust Calcium, serum, low or Calcium, serum, high for albumin.					
Cardiac troponin I (cTnI)		NA	NA	NA	Levels consistent with myocardial infarction or unstable angina as defined by the manufacturer
Cardiac troponin T (cTnT)		NA	NA	NA	≥0.20 ng/mL OR Levels consistent with myocardial infarction or unstable angina as defined by the manufacturer
Cholesterol (fasting)					
	**Adult ≥ 18 years**	200 – 239 mg/dL *5.18 – 6.19 mmol/L*	240 – 300 mg/dL *6.20 – 7.77 mmol/L*	>300 mg/dL *>7.77 mmol/L*	NA
	**Pediatric <18 years**	170 – 199 mg/dL *4.40 – 5.15 mmol/L*	200 – 300 mg/dL *5.16 – 7.77 mmol/L*	>300 mg/dL *>7.77 mmol/L*	NA
Creatine kinase[a]		≥3.0 to ≤5.9 x ULN[a]	>5.9 to ≤9.9 x ULN[a]	>9.9 to ≤19.9 x ULN[a]	>19.9 x ULN[a]
Creatinine[a]		≥1.1 to ≤1.3 x ULN[a]	>1.3 to ≤1.8 x ULN[a]	>1.8 to ≤3.4 x ULN[a]	>3.4 x ULN[a]
Glucose, serum, high					
	Nonfasting	116 – 160 mg/dL *6.44 – 8.88 mmol/L*	161 – 250 mg/dL *8.89 – 13.88 mmol/L*	251 – 500 mg/dL *13.89 – 27.75 mmol/L*	>500 mg/dL *>27.75 mmol/L*
	Fasting	110 – 125 mg/dL *6.11 – 6.94 mmol/L*	126 – 250 mg/dL *6.95 – 13.88 mmol/L*	251 – 500 mg/dL *13.89 – 27.75 mmol/L*	>500 mg/dL *>27.75 mmol/L*

[a] Revised by the Sponsor.
[b] Values are for term infants; preterm infants should be assessed using local normal ranges.
[c] Use age- and sex-appropriate values (eg, bilirubin).

Attachment 3: (Continued)
DAIDS Table

LABORATORY					
**PARAMETER**		**GRADE 1 MILD**	**GRADE 2 MODERATE**	**GRADE 3 SEVERE**	**GRADE 4 POTENTIALLY LIFE-THREATENING**
Glucose, serum, low					
	**Adult and Pediatric ≥1 month**	55 – 64 mg/dL<br>*3.05 – 3.55 mmol/L*	40 – 54 mg/dL<br>*2.22 – 3.00 mmol/L*	30 – 39 mg/dL<br>*1.67 – 2.21 mmol/L*	<30 mg/dL<br>*<1.67 mmol/L*
	**Infant[a,b] <1 month**	50 – 54 mg/dL<br>*2.78 – 3.00 mmol/L*	40 – 49 mg/dL<br>*2.22 – 2.77 mmol/L*	30 – 39 mg/dL<br>*1.67 – 2.21 mmol/L*	<30 mg/dL<br>*<1.67 mmol/L*
Lactate		ULN - <2.0 x ULN without acidosis	≥2.0 x ULN without acidosis	Increased lactate with pH <7.3 without life-threatening consequences	Increased lactate with pH <7.3 with life-threatening consequences
LDL cholesterol (fasting)					
	**Adult ≥18 years**	130 – 159 mg/dL<br>*3.37 – 4.12 mmol/L*	160 – 190 mg/dL<br>*4.13 – 4.90 mmol/L*	≥ 191 mg/dL<br>*≥ 4.91 mmol/L*	NA
	**Pediatric >2– <18 Years**	110 – 129 mg/dL<br>*2.85 – 3.34 mmol/L*	130 – 189 mg/dL<br>*3.35 – 4.90 mmol/L*	≥ 190 mg/dL<br>*≥ 4.91 mmol/L*	NA
Lipase[c]		≥1.1 to ≤1.5 x ULN	>1.5 to ≤3.0 x ULN	>3.0 to ≤5.0 x ULN	>5.0 x ULN
Magnesium, serum, low		1.2 – 1.4 mEq/L<br>*0.60 – 0.70 mmol/L*	0.9 – 1.1 mEq/L<br>*0.45 – 0.59 mmol/L*	0.6 – 0.8 mEq/L<br>*0.30 – 0.44 mmol/L*	<0.60 mEq/L<br>*<0.30 mmol/L*
Pancreatic amylase[c]		≥1.1 to ≤1.5 x ULN	>1.5 to ≤2.0 x ULN	>2.0 to ≤5.0 x ULN	>5.0 x ULN
Phosphate, serum, low					
	**Adult and Pediatric >14 years**	2.5 mg/dL – <LLN<br>*0.81 mmol/L – <LLN*	2.0 – 2.4 mg/dL<br>*0.65 – 0.80 mmol/L*	1.0 – 1.9 mg/dL<br>*0.32 – 0.64 mmol/L*	<1.00 mg/dL<br>*<0.32 mmol/L*
	**Pediatric 1 – 14 years**	3.0 – 3.5 mg/dL<br>*0.97 – 1.13 mmol/L*	2.5 – 2.9 mg/dL<br>*0.81 – 0.96 mmol/L*	1.5 – 2.4 mg/dL<br>*0.48 – 0.80 mmol/L*	<1.50 mg/dL<br>*<0.48 mmol/L*
	**Pediatric <1 year**	3.5 – 4.5 mg/dL<br>*1.13 – 1.45 mmol/L*	2.5 – 3.4 mg/dL<br>*0.81 – 1.12 mmol/L*	1.5 – 2.4 mg/dL<br>*0.48 – 0.80 mmol/L*	<1.50 mg/dL<br>*<0.48 mmol/L*
Potassium, serum, high		5.6 – 6.0 mEq/L<br>*5.6 – 6.0 mmol/L*	6.1 – 6.5 mEq/L<br>*6.1 – 6.5 mmol/L*	6.6 – 7.0 mEq/L<br>*6.6 – 7.0 mmol/L*	>7.0 mEq/L<br>*>7.0 mmol/L*
Potassium, serum, low		3.0 – 3.4 mEq/L<br>*3.0 – 3.4 mmol/L*	2.5 – 2.9 mEq/L<br>*2.5 – 2.9 mmol/L*	2.0 – 2.4 mEq/L<br>*2.0 – 2.4 mmol/L*	<2.0 mEq/L<br>*<2.0 mmol/L*
Sodium, serum, high		146 – 150 mEq/L<br>*146 – 150 mmol/L*	151 – 154 mEq/L<br>*151 – 154 mmol/L*	155 – 159 mEq/L<br>*155 – 159 mmol/L*	≥160 mEq/L<br>*≥160 mmol/L*
Sodium, serum, low		130 – 135 mEq/L<br>*130 – 135 mmol/L*	125 – 129 mEq/L<br>*125 – 129 mmol/L*	121 – 124 mEq/L<br>*121 – 124 mmol/L*	≤120 mEq/L<br>*≤120 mmol/L*
Triglycerides (fasting)		NA	500 – 750 mg/dL<br>*5.65 – 8.48 mmol/L*	751 – 1,200 mg/dL<br>*8.49 – 13.56 mmol/L*	>1,200 mg/dL<br>*>13.56 mmol/L*
Uric acid		7.5 – 10.0 mg/dL<br>*0.45 – 0.59 mmol/L*	10.1 – 12.0 mg/dL<br>*0.60 – 0.71 mmol/L*	12.1 – 15.0 mg/dL<br>*0.72 – 0.89 mmol/L*	>15.0 mg/dL<br>*>0.89 mmol/L*

[a] Values are for term infants; preterm infants should be assessed using local normal ranges.
[b] Use age- and sex-appropriate values (eg, bilirubin).
[c] Revised by the Sponsor.

Attachment 3: (Continued)

DAIDS Table

LABORATORY					
**PARAMETER**		**GRADE 1 MILD**	**GRADE 2 MODERATE**	**GRADE 3 SEVERE**	**GRADE 4 POTENTIALLY LIFE-THREATENING**
**URINALYSIS** ***Standard International Units are listed in italics***					
Hematuria (microscopic)		6 – 10 RBC/HPF	>10 RBC/HPF	Gross, with or without clots OR with RBC casts	Transfusion indicated
Proteinuria, random collection		1 +	2 – 3 +	4 +	NA
Proteinuria, 24 hour collection					
	**Adult and Pediatric ≥10 years**	200 – 999 mg/24 h<br>*0.200 – 0.999 g/d*	1,000 – 1,999 mg/24 h<br>*1.000 – 1.999 g/d*	2,000 – 3,500 mg/24 h<br>*2.000 – 3.500 g/d*	>3,500 mg/24 h<br>*>3.500 g/d*
	**Pediatric >3 months – <10 years**	201 – 499 mg/m^2/24 h<br>*0.201 – 0.499 g/d*	500 – 799 mg/m^2/24 h<br>*0.500 – 0.799 g/d*	800 – 1,000 mg/m^2/24 h<br>*0.800 – 1.000 g/d*	>1,000 mg/ m^2/24 h<br>*>1.000 g/d*

LAST PAGE
