# Supplementary material for: Final Results of the Telaprevir Access Program: FibroScan Values Predict Safety and Efficacy in Hepatitis C Patients with Advanced Fibrosis or Cirrhosis
Source: PLoS One. 2015 Sep 23;10(9):e0138503. doi: 10.1371/journal.pone.0138503 (PMC4580464; doi:10.1371/journal.pone.0138503)
Supplement: S1 Table — (DOCX) [file pone.0138503.s002.docx]

**S1 Table. Efficacy outcome for each subgroup of patients with respect to previous PR treatment and to extended rapid virologic response (eRVR) in the intent-to-treat population.**

| **n (%)** | **Treatment naive (N = 355)** | **Prior relapser (N = 586)** | **Prior null responder (N = 495)** | **Prior partial responder (N = 234)** | **Overall (N = 1772)** |
| --- | --- | --- | --- | --- | --- |
| SVR | 262 (74) | 458 (78) | 218 (44) | 141 (60) | 1139 (64) |
| Virologic failure |  |  |  |  |  |
| Relapse | 28 (8) | 44 (8) | 69 (14) | 40 (17) | 194 (11) |
| Viral breakthrough | 28 (8) | 28 (5) | 122 (25) | 27 (12) | 217 (12) |
| Met a stopping rule | 5 (1) | 8 (1) | 27 (5) | 7 (3) | 54 (3) |
| Other | 4 (1) | 11 (2) | 26 (5) | 5 (2) | 49 (3) |
| n/N (%) |  |  |  |  |  |
| SVR in all patients who achieved eRVR, | 173/210 (82) | 328/383 (86) | 128/203 (63) | 90/124 (73) | 760/973 (78) |
| SVR in patients with bridging fibrosis who achieved eRVR | 93/111 (84) | 174/196 (89) | 60/77 (78) | 43/54 (80) | 394/468 (84) |
| SVR in patients with cirrhosis who achieved eRVR | 80/99 (81) | 154/187 (82) | 68/126 (54) | 47/70 (67) | 366/505 (72) |
| SVR in patients who did not achieve eRVR | 89/145 (61) | 130/203 (64) | 90/292 (31) | 51/110 (46) | 379/799 (47) |
| SVR in patients with bridging fibrosis who did not achieve eRVR | 43/65 (66) | 58/92 (63) | 47/121 (39) | 24/99 (49) | 179/345 (52) |
| SVR in patients with cirrhosis who did not achieve eRVR | 46/80 (58) | 72/111 (65) | 43/171 (25) | 27/61 (44) | 200/454 (44) |
